# Four New Perforane-Type Sesquiterpenes from *Laurencia obtusa* (Hudson) J.V. Lamouroux as Potent Lung Cancer Inhibitors: Isolation, Structure Elucidation, Cytotoxicity, Molecular Docking, Dynamics, and ADME Studies

**DOI:** 10.1021/acsomega.5c08806

**Published:** 2026-01-12

**Authors:** Özlem Demirkıran, Halil Şenol, Yağmur Elçi, Elif Coşkun, Gülbahar Özge Alim Toraman, Ebru Erol, Emine Şükran Okudan, Gülaçtı Topçu

**Affiliations:** † Department of Pharmacognosy, Faculty of Pharmacy, 37521 Trakya University, 22030 Edirne, Türkiye; ‡ Department of Pharmaceutical Chemistry, Faculty of Pharmacy, 221265 Bezmialem Vakif University, 34093 Fatih, Istanbul, Türkiye; § Department of Pharmacognosy, Faculty of Pharmacy, 221265 Bezmialem Vakif University, 34093 Fatih, Istanbul, Türkiye; ∥ Department of Analytical Chemistry, Faculty of Pharmacy, 221265 Bezmialem Vakif University, 34093 Fatih, Istanbul, Türkiye; ⊥ Faculty of Aquatic Sciences and Fisheries, 37502 Akdeniz University, 07058 Antalya, Türkiye; # Drug Application and Research Center, 221265 Bezmialem Vakif University, 34093 Fatih, Istanbul, Türkiye

## Abstract

The red alga genus *Laurencia* (Rhodomelaceae) is a prolific source of halogenated secondary metabolites, particularly sesquiterpenes with diverse carbon skeletons and significant biological activities. In this study, four new perforane-type sesquiterpenes (**1**–**4**), including two halogenated and two nonhalogenated compounds, were isolated from *Laurencia obtusa* (Hudson) J. V. Lamouroux. Their chemical structures were elucidated using comprehensive spectroscopic techniques, including 1D- and 2D-NMR, and LC-HRMS. The cytotoxic potential of the isolated compounds was evaluated against human lung adenocarcinoma (A549) cells, revealing notable antiproliferative effects. To explore the molecular basis of their activity, molecular docking and molecular dynamics simulations were performed targeting key oncogenic receptors VEGFR1, VEGFR2, and EGFR. The results demonstrated strong binding affinities and stable interactions within the active sites of these targets. Furthermore, ADMET analyses predicted favorable pharmacokinetic profiles and acceptable toxicity parameters for the isolated compounds. These findings suggest that the newly identified perforane-type sesquiterpenes from *L. obtusa* hold promise as potential candidates for the development of novel anticancer agents targeting lung cancer.

## Graphical abstract

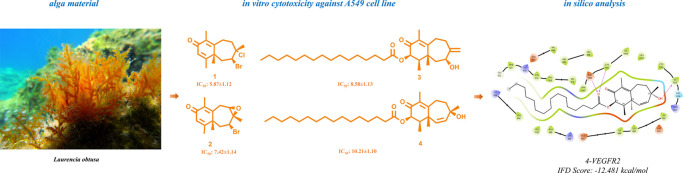

## 1 Introduction

Red algae belonging to the genus *Laurencia* (Rhodophyta, Ceramiales, and Rhodomelaceae) include about 140 species distributed in warm seas, especially on temperate and tropical coasts.^1^ They have been recognized as a rich source of uniquely specific metabolites with a high degree of halogenation, particularly bromination, and a wide variety of skeleton types.^2^ Genus *Laurencia* (Rhodomelaceae) is considered to be one of the most studied genera in the marine environment,^3^ and >700 compounds with unique structures have been isolated from more than 60 species studied.^4^ A comprehensive study by Harizani et al. showed that *Laurencia* species contain an endless source of secondary metabolites, including acetogenins, diterpenoids, triterpenoids, meroterpenoids, indole alkaloids, steroids, several aromatic compounds, and predominantly sesquiterpenes.^5^

More than 500 sesquiterpenes isolated from *Laurencia* species, which have a remarkable capacity to biosynthesize sesquiterpenes, have been reported to have predominantly chamigrane, aristolane, cuprane, bisabolane, laurane, snyderane and brasilane skeletons.^6,7^ Sesquiterpenes from *Laurencia* species have shown different activities: cytotoxic,^8–13^ anti-inflammatory,^14–16^ antibacterial,^16–22^ antifungal,^21,23,24^ antiparasitic,^25,26^ antidiabetic,^27–29^ antimalarial,^30^ and anthelmintic activities,^31^ as well as different ecological roles.^32–35^ Perforanes, a relatively rare group of sesquiterpenes isolated from *Laurencia* spp., contain only 22 members^5^ and were obtained from *Laurencia obtusa*,^10,36^ *L. snyderae*,^37^ *L. perforate*,^38–40^ and mollusks that feed on them.^41^

Cancer is a group of diseases characterized by uncontrolled cell growth that can spread to other parts of the body. Lung cancer, one of the most common and deadly types of cancer, is often linked to smoking and environmental factors. It is primarily classified into small-cell lung cancer (SCLC) and non-small-cell lung cancer (NSCLC), with NSCLC being more common. Due to its aggressive nature and late diagnosis, lung cancer has a low survival rate, highlighting the need for early detection and targeted therapies. VEGFR1, VEGFR2, and EGFR are key receptors involved in tumor growth and metastasis. VEGFRs promote angiogenesis, supporting tumor growth, and EGFR regulates cell proliferation and survival. Inhibiting VEGFR1, VEGFR2, and EGFR is vital in lung cancer treatment, as these receptors drive tumor growth and metastasis. VEGFRs promote angiogenesis, supplying tumors with nutrients and oxygen, while EGFR regulates cell proliferation and survival.^42^ Targeting these receptors can reduce tumor vascularization, slow cell growth, and enhance the effectiveness of other therapies, ultimately improving treatment outcomes.^43^

Molecular docking, molecular dynamics (MD) simulations, and ADMET (Absorption, Distribution, Metabolism, Excretion, and Toxicity) prediction are pivotal tools in modern drug design. Molecular docking helps identify the binding affinity and interactions between a drug and its target, while MD simulations provide insights into the stability and dynamics of ligand–receptor complexes over time. ADMET prediction models the pharmacokinetic properties and potential toxicity of compounds, allowing for the early identification of promising drug candidates with favorable safety and efficacy profiles.

In this study, we isolated and characterized four new perforane-type sesquiterpenes comprising two halogenated and two nonhalogenated structures from *Laurencia obtusa* (Hudson) J. V. Lamouroux. The study focused on elucidation of the chemical structures of the isolates using comprehensive spectroscopic techniques and tested for potential anticancer activity. Cytotoxicity assays were performed to assess their effects on human lung adenocarcinoma cells, and molecular docking and MD simulations were employed to predict their binding affinity and stability in interactions with cancer-related targets. Additionally, ADMET analysis was conducted to predict the pharmacokinetic properties and toxicity profiles of these compounds, providing a comprehensive evaluation of their therapeutic potential.

## 2 Results and Discussion

### 2.1 Isolation and Structure Elucidation

The red alga *L. obtusa* was collected from Çanakkale, Yapıldakaltı region in the Aegean Sea, dried, and then extracted with CH_2_Cl_2_:MeOH (1:1) for its secondary metabolites. The extract was fractionated using normal-phase vacuum liquid chromatography (VLC) on silica gel, and the relatively less polar fraction (50% EtOAc/hexane) was subjected to column chromatography on silica gel to yield compounds **1–4** (Figure 1).

**Figure 1:**
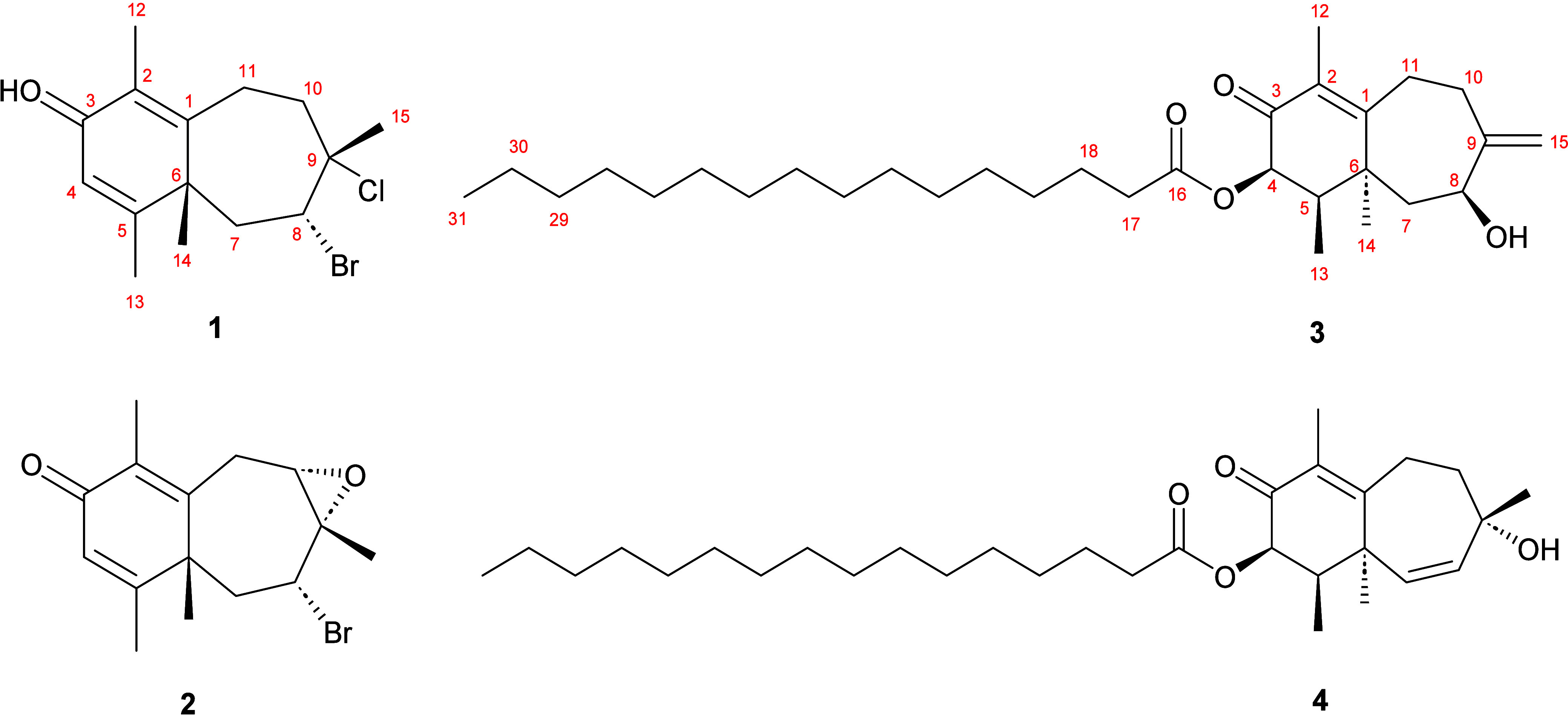
Structures of the new compounds are isolated from *L. obtusa*.

HR-ESI-MS of compound **1** provided clear molecular ion signals at *m*/*z* 331.04602 ([M + H]^+^ for C_1__5_H_2__1_O^79^Br^35^Cl), *m*/*z* 333.04346 ([M + H]^+^ for C_1__5_H_2__1_O^81^Br^35^Cl), and a minor isotopomer at *m*/*z* 335.04031 ([M + H]^+^ for C_1__5_H_2__1_O^81^Br^37^Cl). The observed 1:1:0.3 isotopic distribution pattern is in excellent agreement with the presence of one bromine atom and one chlorine atom in the molecule. The measured *m*/*z* values showed less than 2 ppm deviation from the calculated masses (*m*/*z* 331.04643, 333.04420, and 335.0420), confirming the molecular formula C_1__5_H_2__0_BrClO (Figures S7 and S8). These three molecular ion peaks indicated a molecular formula of C_15_H_20_^79^Br^35^ClO requiring five degrees of unsaturation. In the ^1^H NMR spectrum of compound **1**, two signals observed at δ_H_ 6.28 and 3.78 were distinguishable from other signals observed in the upfield region between δ_H_ 1.00–2.80 (Figure S1). The carbon chemical shift value of the proton signal observed at δ_H_ 6.28 in ^1^H NMR was assigned to δ_C_ 129.0 in the HSQC spectrum, and this was indicative of an olefinic structure. The signal observed at δ_H_ 3.78 in the ^1^H NMR spectrum was deduced as either an oxy- or halo-methine proton. Its carbon chemical shift observed at δ_C_ 58.9, which is characteristic of a bromine-bearing carbon, together with the detailed analysis of HR-MS, indicated the presence of a bromine-containing structure. In addition, three methylene (−CH_2_) signals [δ_C_ 44.7, 41.7, and 25.8] and four methyl (CH_3_) signals [δ_C_ 29.7, 24.9, 20.3, and 10.9] were observed in the HSQC spectrum (Figure S4). The presence of six quaternary carbons among a total of 15 carbon atoms suggests that the compound likely possesses a sesquiterpene structure. It was observed that the olefinic hydrogen at δ_H_ 6.28 (H-4) showed three-bond correlations to δ_C_ 132.4 (C-2), 46.6 (C-6), and 20.3 (C-13) in HMBC (Figure S5). In particular, the correlations of multiple protons H-7, H-10, H-11, H-12, and H-14 with C-1 (δ_C_ 160.5) and of H-4, H-7, H-8, H-13, and H-14 with C-6 (δ_C_ 46.7) clearly indicate that these carbon atoms are located at the fusion points of the bicyclic framework. Due to the bicyclic ring structure with six- and seven-membered rings, many cross-correlations were observed in the HMBC spectrum and are shown in Figure 2 and Table S1. The dienone structure of the six-membered ring was deduced based on chemical shifts at δ_C_ 132.4 (C-2), 129.0 (C-4), and in the more downfield region at δ_C_ 160.5 (C-1), 161.6 (C-5) in the six-membered ring.^39^ Four singlet methyl groups were observed at δ_H_ 1.21, 1.86, 1.94, and 2.11, which were determined as C-1, C-4, C-5, and C-8 based on HMBC correlations (Figure 2). The presence of a Cl atom was deduced based on the carbon chemical shift at δ_C_ 72.8.^40^ The locations of chlorination and bromination were deduced based on HMBC correlations, as shown in Figure 2. All correlations are presented in Table S1. Using the data obtained, it was concluded that the structure of the molecule is a sesquiterpene with a perforane skeleton.

**Figure 2:**
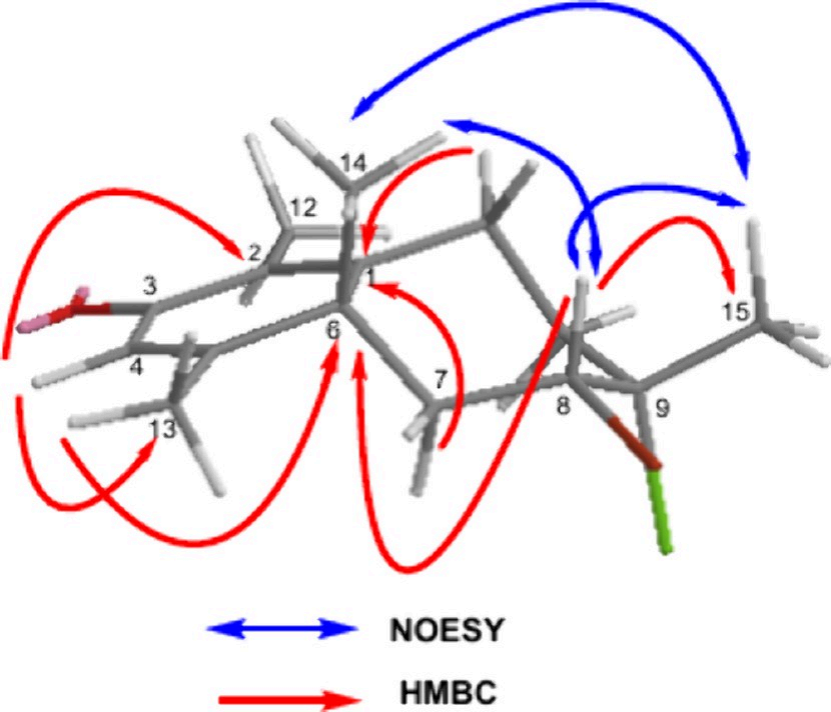
Key NOESY and HMBC correlations for compound **1**.

Relative configurations for chiral centers were determined by the NOESY spectrum (Figure S6). The correlations among H-14/H-15, H-8/H-15, and H-13/H-8 suggested that these protons share the same space. The key NOESY correlations used to determine the relative configuration, as shown in Figure 2. Moreover, the coupling constant of H-8 (d, *J* = 10.2 Hz) suggested that H-8 is in the axial configuration. If H-8 were in an equatorial orientation, its multiplicity would be expected as a doublet of doublets (dd) due to the nearly equal dihedral angles it shares with the CH_2_-7 protons. However, since H-8 has a perpendicular dihedral angle with the CH_2_-7α proton (δ2.84, d, *J* = 16.3 Hz), it is only coupled with anti-CH_2_-7β (δ_H_ 2.35, dd, *J* = 16.4, 10.3 Hz) and gave a doublet. Consequently, compound **1**, named obtusadienone A, has an IUPAC formula 8-bromo-9-chloro-2,5,6,9-tetramethylbicyclo-[5.4.0^1,6^]-1,4-undecadiene-3-one.

The detailed analysis of ^1^H NMR and ^13^C NMR spectra of compound **2** exhibited notable similarity to compound **1** (Figures S11 and S12). The most significant difference in the ^13^C NMR spectrum was the appearance of two signals resonating at δ_C_ = 61.1 and 72.9. It was concluded from the APT spectrum that one of these signals was observed at δ_C_ 72.9 as a quaternary carbon, while the other one was observed at δ_C_ 61.1 as an oxymethine carbon, and its proton was observed at δ_H_ 2.82 based on the HSQC spectrum, indicating the presence of a substituted epoxy ring. The HMBC correlations from the proton resonated at δ_H_ 2.82 to the carbons at δ_C_ 160.4 (C-1), 46.7 (C-6), 58.9 (C-8), and 72.9 (C-9) indicated that the epoxy ring was positioned at carbons C-9 and C-10. The location of one of the methyl groups resonated at δ_H_ 1.85 showed two- and three-bond away HMBC correlations with C-9 and C-8, respectively. The location of the other methyl groups was determined to be at C-2, C-5, and C-6 by HMBC correlations (Figure S15). All correlations are presented in Table S2.

Using the data obtained, it was concluded that the structure of the molecule is a sesquiterpene with a perforane skeleton. Relative configurations of the asymmetric centers of the structure were proposed based on NOESY correlations (Figure S16). The correlations between H-8/H-15 and H-13/H-8 confirmed the *axial* configurations of the methyl groups. Similar to compound **1**, the coupling constant of H-8 (d, *J* = 10.2 Hz) suggested that H-8 is in the *axial* configuration. Moreover, a NOESY correlation between H-8 and H-10 suggested that the epoxy ring was *equatorial*. The important NOESY correlations used to determine the relative configuration are listed in Figure 3.

**Figure 3:**
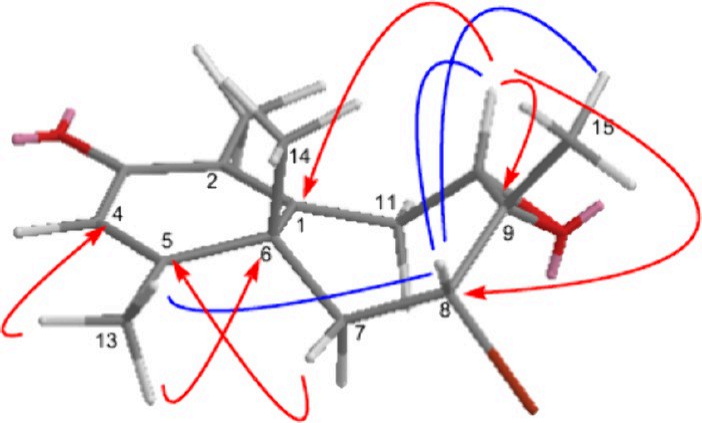
Key NOESY and HMBC correlations for compound **2**.

In the HR-ESI-MS spectrum (positive-ion mode) of compound **2**, no detectable [M + H]^+^ ion was observed, while the sodium adduct signals predominated. This behavior is consistent with the well-known ionization preference of halogenated oxygenated sesquiterpenes toward sodium adduct formation under ESI­(+) conditions.^41^ Two distinct and nearly equi-intense peaks were detected at *m*/*z* 317.05066 and 319.04852, displaying the expected 1:1 bromine isotopic pattern. These ions are tentatively assigned to a [M–O + Na]^+^ adduct, most likely generated *in source* from the epoxide-containing precursor (C_1__5_H_1__9_BrO_2_ → C_1__5_H_1__9_BrO). The absence of any ion near *m*/*z* 315 further supports this interpretation, indicating that the 317/319 pair originates from an *in-source* oxygen-loss process rather than dehydration (see Figures S17 and S18). Consequently, compound **2**, named obtusadienone B, has an IUPAC formula 8-bromo-9,10-epoxy-2,5,6,9-tetramethylbicyclo-[5.4.0^1,6^]-1,4-undecadiene-3-one.

The similarity of the ^1^H- and ^13^C NMR spectra of compound **3** with those of **1** and **2** indicates that the structure has a perforane sesquiterpene skeleton (Figures S21 and S22). The NMR data are given in Table S3 for compound **3**. The most important difference observed was the presence of signals at δ_H_ values of 4.86 (d, *J* = 1.5 Hz) and 5.06 (d, *J* = 1.5 Hz). The coupling constant and HSQC correlations, indicating that the protons are attached to the same carbon atom, confirm the presence of an exocyclic double bond. The chemical shift of the quaternary olefinic carbon was determined as δ_C_ 151.0 (C-9) via HMBC correlations from exocyclic double-bond protons.

In the ^1^H NMR, the signals observed at δ_H_ 5.58 and 4.10 were determined as oxymethine protons that were also supported by carbon chemical shift values at δ_C_ 73.5 and 72.4, respectively. The location of the proton observed at δ_H_ 5.58 was determined to be C-4, considering its HMBC correlations with carbons C-3, C-5, and C-16. The location of the second oxymethine proton (δ_H_ 4.10) was determined as C-8 thanks to the HMBC correlations that were shown with carbons C-5 and C-6. Another remarkable group of the peak in the spectrum was a signal belonging to a hydrocarbon chain with a high integral value observed at 1.25–130 ppm in the high magnetic field. Two key HMBC correlations were observed from δ_H_ 5.58 (H-4) and 2.37 (H-17) to the same ester carbon signal at δ_C_ 173.1, suggesting that the placement of the long chain was at δ_C_ 73.5 (C-4). The presence of the terminal methyl group of the chain was observed resonating at δ 0.88 (H-31) as a triplet, which was verified by HMBC and COSY correlations at δ 1.29 (H-30) and 1.25 (H-29) (Figures S23–S25). The integral of the signal observed at 1.28 ppm indicates the presence of 20 protons; hence, 10 methylene protons revealed an overlapping chemical shift. The information obtained from the NMR data that the side chain has 16 carbons, including the ester carbonyl, was supported by the molecular ion peak observed in HRMS. Accordingly, in HRMS, the sodium adduct ion [M + Na]^+^ observed at *m*/*z* 511.37500 agreed with the molecular formula C_31_H_52_O_4_Na (calcd. for 511.37633).

Exocyclic double bond protons were determined by NOESY correlations (Figure S26). H-8 showed a correlation with exocyclic double-bond protons H-15a (δ_H_ 5.06) and H-15b (δ_H_ 4.86). However, H-10a (2.69 ppm) showed a NOESY correlation with H-15b, only. Relative configurations of the chiral centers were determined by the NOESY spectrum. The correlations among H-4/H-13, H-4/H-8, and H-5/H-14 suggested that these protons share the same space. Key NOESY correlations used to determine the relative configuration are shown in Figure 4. Consequently, compound **3**, named obtusaenone A, has an IUPAC formula 4-hexadecanoyloxy-8-hydroxy-9-exomethylene-2,5,6-trimethylbicyclo-[5.4.0^1,6^]-undeca-1-ene-3-one.

**Figure 4:**
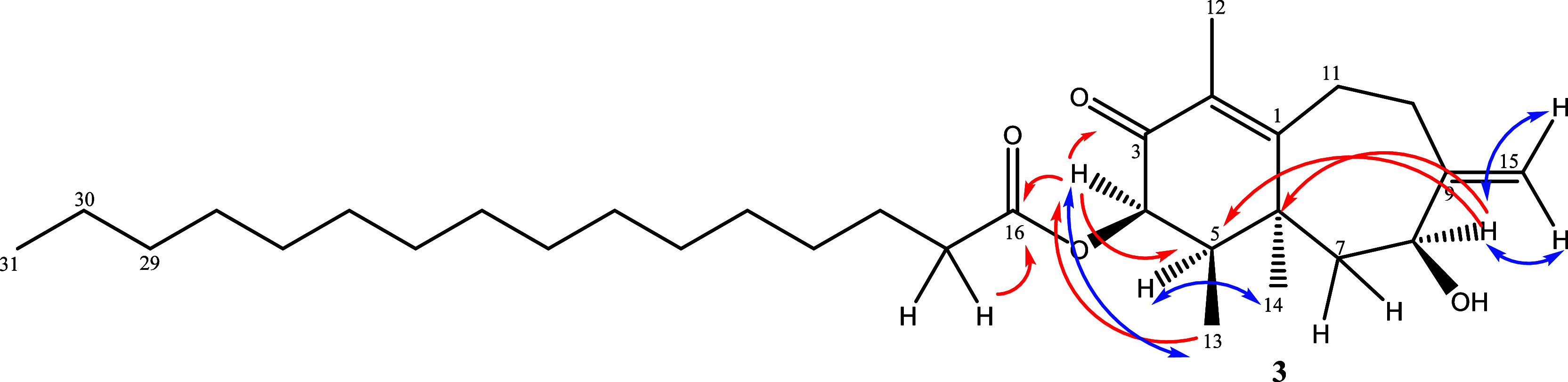
Key NOESY and HMBC correlations for compound **3**.

The ^1^H NMR spectrum of **4** was similar to that of compound **3**, and two doublets at δ_H_ 5.79 and 5.63 with *J* = 12.0 Hz coupling constant indicated the presence of a double bond in the ring (Figure S30). The HMBC correlations from δ_H_ 5.79 to C-1, C-5, and C-9 and from δ_H_ 5.63 to C-6, C-10, and C-15 suggested the location of the double bond between C-7 and C-8. It was understood that one of the methyl signals observed at δ_H_ 1.27 was attached to an oxygen-bearing quaternary carbon, which is observed at δ_C_ 72.0, as confirmed by the HMBC correlation (Figure S34). Other HMBC correlations from this methyl at δ_H_ 1.27 to C-10 (δ_C_ 39.1) and C-8 (δ_C_ 138.3) suggested its location at C-9 (Table S4).

The significant HMBC correlations from δ_H_ 5.59 (H-4) to the ester carbon signal at δ_C_ 173.2 confirm the attachment of the long chain to the bicyclic ring system. Similar to compound **3**, a long hydrocarbon chain was established by an intense signal at δ_H_ 1.28 in the ^1^H NMR. Relative configurations of chiral centers were determined by the NOESY experiment (Figure S35). The correlations among H-4/H-5, H-8/H-15, H-7/H-14, H-7/H-5, and H-7/H8 suggested that these protons share the same space. Key NOESY correlations used to determine the relative configuration are shown in Figure 5. The molecular ion peak observed in the HRESI-MS analysis suggested that the side chain has a total length of 14 methylene units. Its molecular formula was established as C_31_H_52_O_4_ by positive-ion HRESI-MS of the sodiated molecular ion [M + Na]^+^ at *m*/*z* 511.37479 (calcd. for 511.37633; Figure S36). Consequently, compound **4**, named obtusaenone B, has an IUPAC formula 4-hexadecanoyloxy-9-hydroxy-2,5,9-trimethylbicyclo-[5.4.0 ^1,6^ ]-undeca-1,7-diene-3-one.

**Figure 5:**
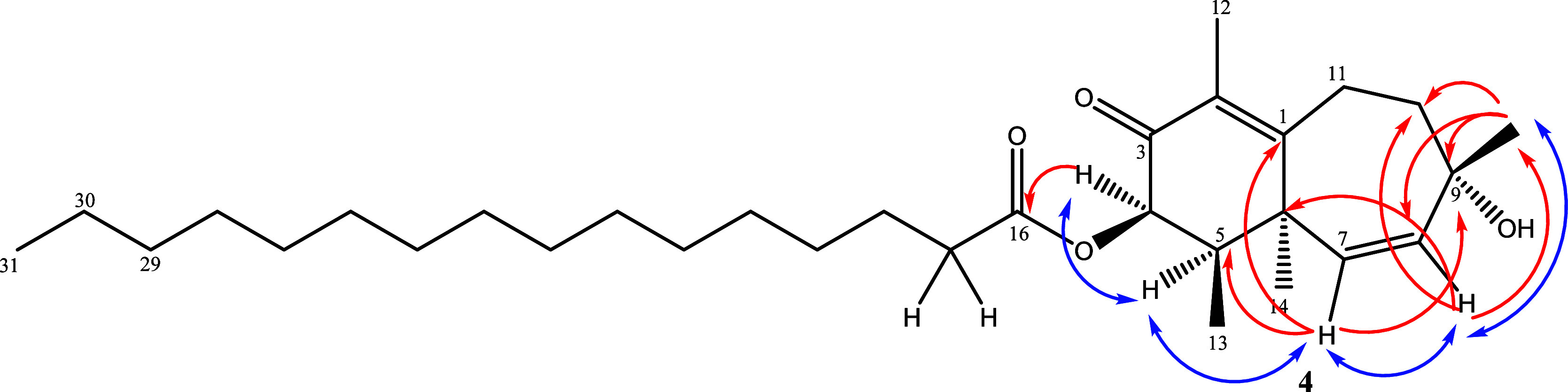
Key NOESY and HMBC correlations for compound **4**.

### 2.2 *In Vitro* Cytotoxic Activity

To evaluate the cytotoxic effects of the isolated new compounds, their activities were tested against both A549 (human lung adenocarcinoma) and BEAS-2B (normal bronchial epithelial) cell lines. The cell viability results of the compounds isolated from *L. obtusa* are presented in Figure 6. The selectivity index (SI) was calculated to determine the compounds’ specificity toward cancer cells relative to normal cells. This assessment aimed to identify compounds with potent anticancer activity while ensuring minimal toxicity to healthy cells. The cytotoxic activity and selectivity results are summarized in Table 1. According to the results, all compounds demonstrated significant cytotoxicity against A549 cells, with IC_50_ values ranging from 5.87 to 10.21 μM. Compound **1** exhibited the highest potency (IC_50_ = 5.87 μM), followed by compound **2** (IC_50_ = 7.42 μM).

**Table 1:** Cytotoxic Activity Results of the Isolated Compounds from *L. obtusa* (48 h)^a^

	IC_50_ [μM]		selectivity index (SI)
compounds	A549	BEAS-2B	BEAS-2B/A549
**1**	5.87 ± 1.12	127.60 ± 1.27	21.7
**2**	7.42 ± 1.14	159.90 ± 1.31	21.5
**3**	8.58 ± 1.13	98.97 ± 1.16	11.5
**4**	10.21 ± 1.10	121.30 ± 1.29	11.8
doxorubicin	11.50 ± 0.20	93.80 ± 2.41	8.16

a Doxorubicin was used as a reference drug. Data were presented as mean ± standard deviation of individual experiments performed in three parallel measurements (*p* < 0.05).

**Figure 6:**
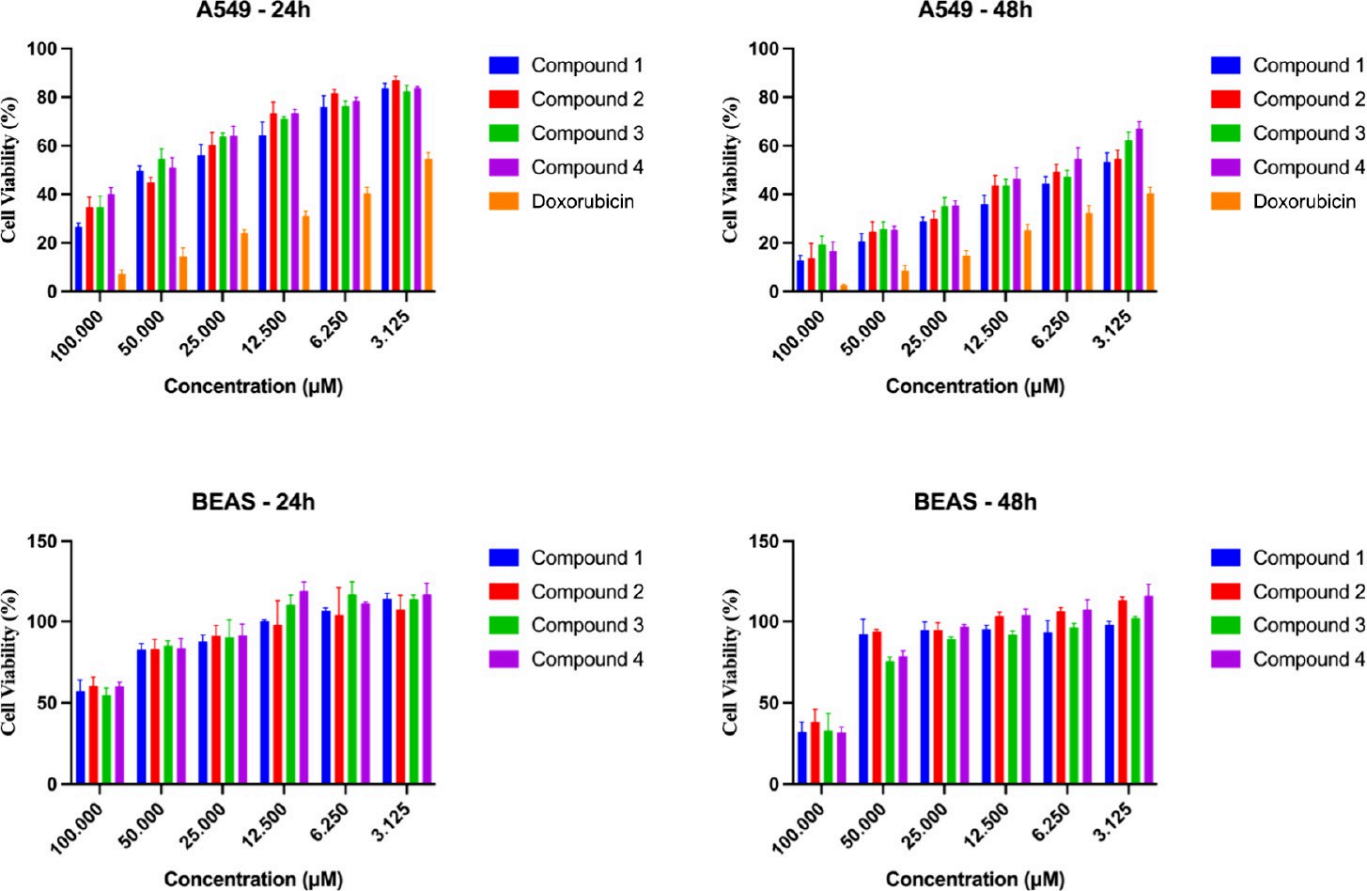
Cell viability results of the isolated compounds from *L. obtusa* (24 and 48 h).

The selectivity index (SI), used to assess specificity toward cancer cells, was the highest for compound **1** (21.7) and compound **2** (21.5), indicating strong selectivity. Compounds **3** and **4** showed moderate selectivity, with SI values of 11.5 and 11.8, respectively.

In comparison, doxorubicin displayed a lower SI (8.16), suggesting greater toxicity to normal cells. In summary, the isolated compounds from *L. obtusa*, particularly compounds **1** and **2**, demonstrated strong cytotoxic effects against A549 cancer cells and high selectivity indices, outperforming the reference drug doxorubicin in terms of safety and efficacy.

### 2.3 Molecular Docking Studies

In this study, molecular docking studies were performed to evaluate the binding affinity and potential inhibitory effects of the isolated compounds against key cancer-related targets, including VEGFR1, VEGFR2, and EGFR. The docking analysis aimed to identify the interactions between the compounds and these receptors, which are critical for angiogenesis and tumor growth. The MM-GBSA (molecular mechanics generalized Born surface area) and IFD (induced fit docking) scores were calculated to further assess the binding strength and stability of the ligand–receptor complexes. These docking results, along with the associated binding energies, are summarized in Table 2, providing valuable insights into the compounds’ potential as targeted cancer therapies. Docking scores and MM-GBSA ΔG binding free energies of isolated compounds are listed in Table 2.

**Table 2:** Docking Scores and MM-GBSA ΔG Binding Free Energies of Isolated Compounds

	docking scores (kcal/mol)			MM-GBSA Δ*G*_Bind._ (kcal/mol)		
compounds	VEGFR1	VEGFR2	EGFR	VEGFR1	VEGFR2	EGFR
**1**	–10.102	–8.476	–5.352	–54.93	–40.19	–32.56
**2**	–8.832	–10.180	–7.390	–51.76	–45.88	–43.68
**3**	–10.045	–9.352	–8.315	–92.97	–78.77	–61.17
**4**	–9.869	–12.481	–9.069	–83.63	–88.34	–72.94

*In vitro* cytotoxicity studies revealed that compounds **1** and **2** were the most active against the A549 lung cancer cell line and showed a high selectivity index. Molecular docking results showed that compound **1** had the strongest affinity for VEGFR1 (−10.102 kcal/mol) and VEGFR2 (−8.476 kcal/mol), while compound **2** exhibited excellent binding to VEGFR2 (−10.180 kcal/mol) and moderate interactions with VEGFR1 (−8.832 kcal/mol) and EGFR (−7.390 kcal/mol). MM-GBSA binding free energy analysis supported these findings, with compound **1** showing stable interactions with VEGFR1 (−54.93 kcal/mol) and VEGFR2 (−40.19 kcal/mol), and compound **2** demonstrating slightly stronger stability with VEGFR2 (−45.88 kcal/mol) and EGFR (−43.68 kcal/mol).

However, compounds **3** and **4** showed superior molecular interactions overall. Compound **3** exhibited the best binding energy for VEGFR1 (−92.97 kcal/mol), along with strong interactions with VEGFR2 (−9.352 and −78.77 kcal/mol) and EGFR (−8.315 and −61.17 kcal/mol). Compound **4** demonstrated the strongest docking score for VEGFR2 (−12.481 kcal/mol) and a high binding stability with VEGFR1 (−83.63 kcal/mol) and EGFR (−72.94 kcal/mol). These results highlight compound **3** as the most promising for VEGFR1 inhibition, while compounds **3** and **4** exhibit strong potential as multitarget inhibitors, complementing the potent *in vitro* activity of compounds **1** and **2.**

#### 2.3.1 Molecular Docking Analysis on VEGFR1

Based on *in vitro* and *in silico* results, compounds **1** and **2** demonstrated notable anticancer potential *in vitro* and showed comparable scores and ligand–protein affinities *in silico*. Molecular docking ligand–protein interaction (LPI) analyses were conducted for all compounds, focusing on the most promising targets, VEGFR1 and VEGFR2. The 2D and 3D LPI profiles of these compounds with VEGFR1 are presented in Figures 7 and 8, respectively.

**Figure 7:**
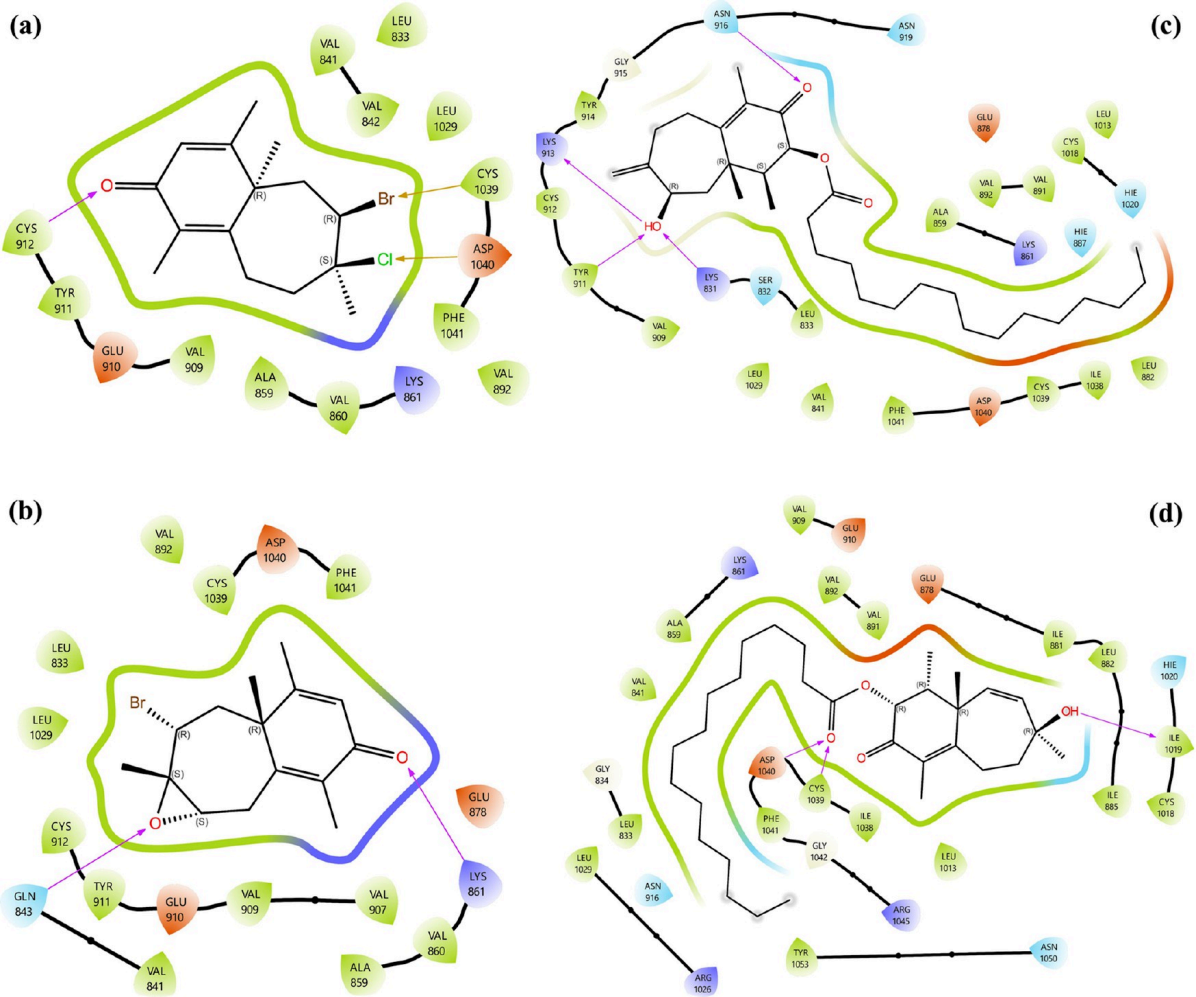
Molecular docking 2D LPI between isolated compounds and VEGFR1 (a) compound **1**, (b) compound **2**, (c) compound **3**, and (d) compound **4**.

**Figure 8:**
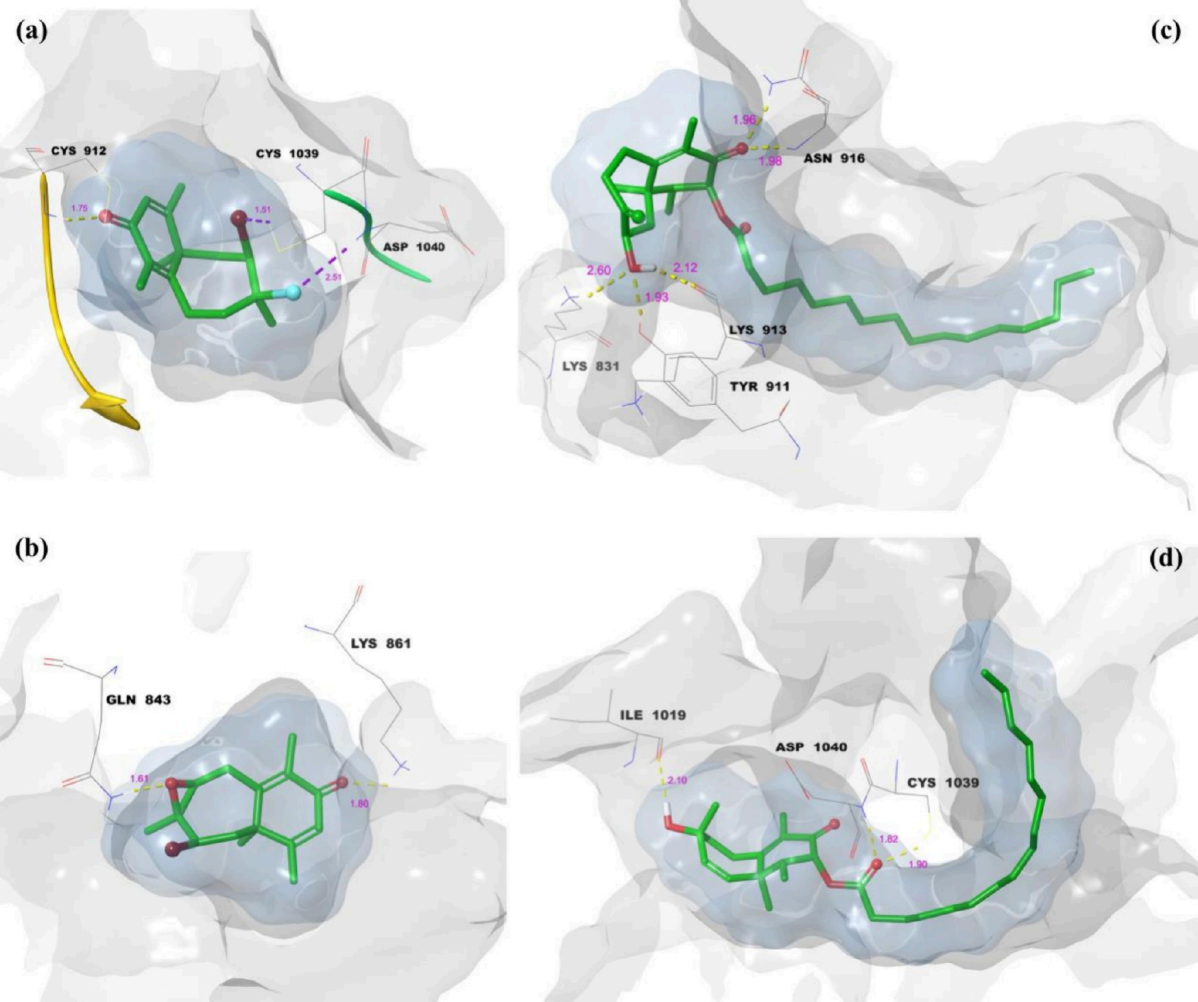
Molecular docking 3D LPI between isolate compounds and VEGFR1: (a) compound **1**, (b) compound **2**, (c) compound **3**, and (d) compound **4**.

Hydrogen bonding was the dominant interaction type across all compounds, with Asp-1040 consistently playing a central role. For compound **1**, the oxygen atom of the ketone carbonyl formed a hydrogen bond with Cys-912, while its bromine and chlorine atoms established halogen bonds with Cys-1039 and Asp-1040, respectively, highlighting the importance of halogen interactions in stabilizing the complex (Figure 7a). Compound **2** displayed a different interaction pattern, with the oxygen of the ketone carbonyl forming a hydrogen bond with Lys-861, and the epoxide oxygen contributing another hydrogen bond with Gln-843, providing a diverse interaction network (Figure 7b).

Compound **3** showed strong binding to VEGFR1, with the oxygen atom of its α, β-unsaturated carbonyl group forming two hydrogen bonds with Asn-916, while the hydroxyl group simultaneously established hydrogen bonds with Lys-913, Tyr-911, and Lys-831 (Figure 7c). Compound **4** exhibited similar strong interactions, with its ester carbonyl oxygen forming two distinct hydrogen bonds with Cys-1039 and Asp-1040, while its hydroxyl group established an additional hydrogen bond with Ile-1019. These results indicate that Asp-1040 and Cys-1039 are key residues across all complexes, consistently involved in stabilizing ligand binding (Figure 7d).

Figure 8 illustrates the 3D LPI between isolated compounds **(1–4)** and VEGFR1. The hydrogen bonds are represented by yellow dashes, while purple dashes depict halogen bonds. The gray surface (protein surface binding area) and blue surface (ligand surface binding area) demonstrate the complementarity between the ligand and the receptor. In Figure 8a, the 3D LPI of the 1-VEGFR1 complex shows hydrogen bond and halogen bond lengths of 1.75, 1.51, and 1.52 Å, respectively. The ligand and protein surface binding areas completely overlap, indicating that the ligand is perfectly embedded within the active site of VEGFR1, forming a stable complex. Figure 8b depicts the 2-VEGFR1 complex, where hydrogen bond lengths of 1.61 and 1.80 Å are observed. Similar to compound **1**, the surface binding areas of the ligand and protein are fully aligned, demonstrating a strong and stable interaction within the active site.

The 3D LPI of the 3-VEGFR1 complex is shown in Figure 8c, with hydrogen bond lengths of 1.93 and 2.60 Å. The ligand’s and protein’s surface areas are entirely overlapping, ensuring tight binding. Additionally, the 16-carbon side chain of compound 3 exhibits free movement within the binding region, facilitating complete ligand accommodation and contributing to a highly stable complex. Finally, in Figure 8d, the 3D LPI of the 4-VEGFR1 complex features hydrogen bond lengths of 1.82, 1.90, and 2.10 Å. Like compound **3**, the 16-carbon side chain in compound **4** moves freely within the binding region, allowing the ligand to fully settle into the active site. This flexibility results in the formation of a highly stable complex. Overall, Figure 8 demonstrates that all compounds are well-integrated into the VEGFR1 binding site, with complete overlap of ligand and protein surface areas and critical interactions such as hydrogen and halogen bonding. Compounds **3** and **4** stand out due to the stabilizing effect of their flexible 16-carbon side chains, enhancing their binding efficiency and complex stability.

#### 2.3.2 Molecular Docking Analysis on VEGFR2

The 2D and 3D LPI profiles of these compounds with VEGFR2 are presented in Figures 9 and 10, respectively.

**Figure 9:**
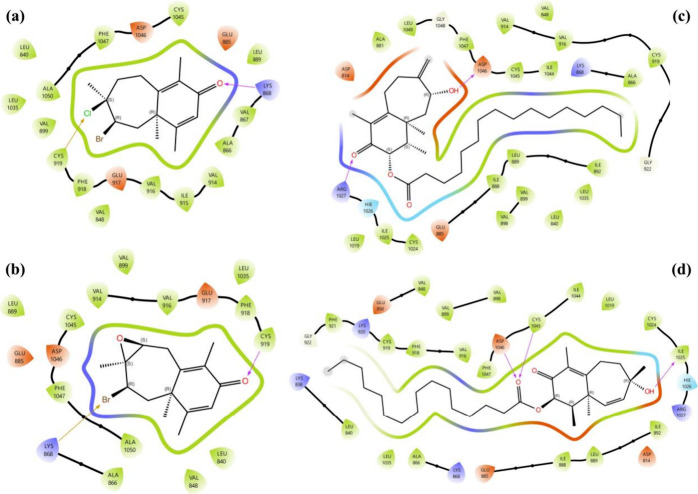
Molecular docking 2D LPI between isolated compounds and VEGFR2: (a) compound **1**, (b) compound **2**, (c) compound **3**, and (d) compound **4**.

**Figure 10:**
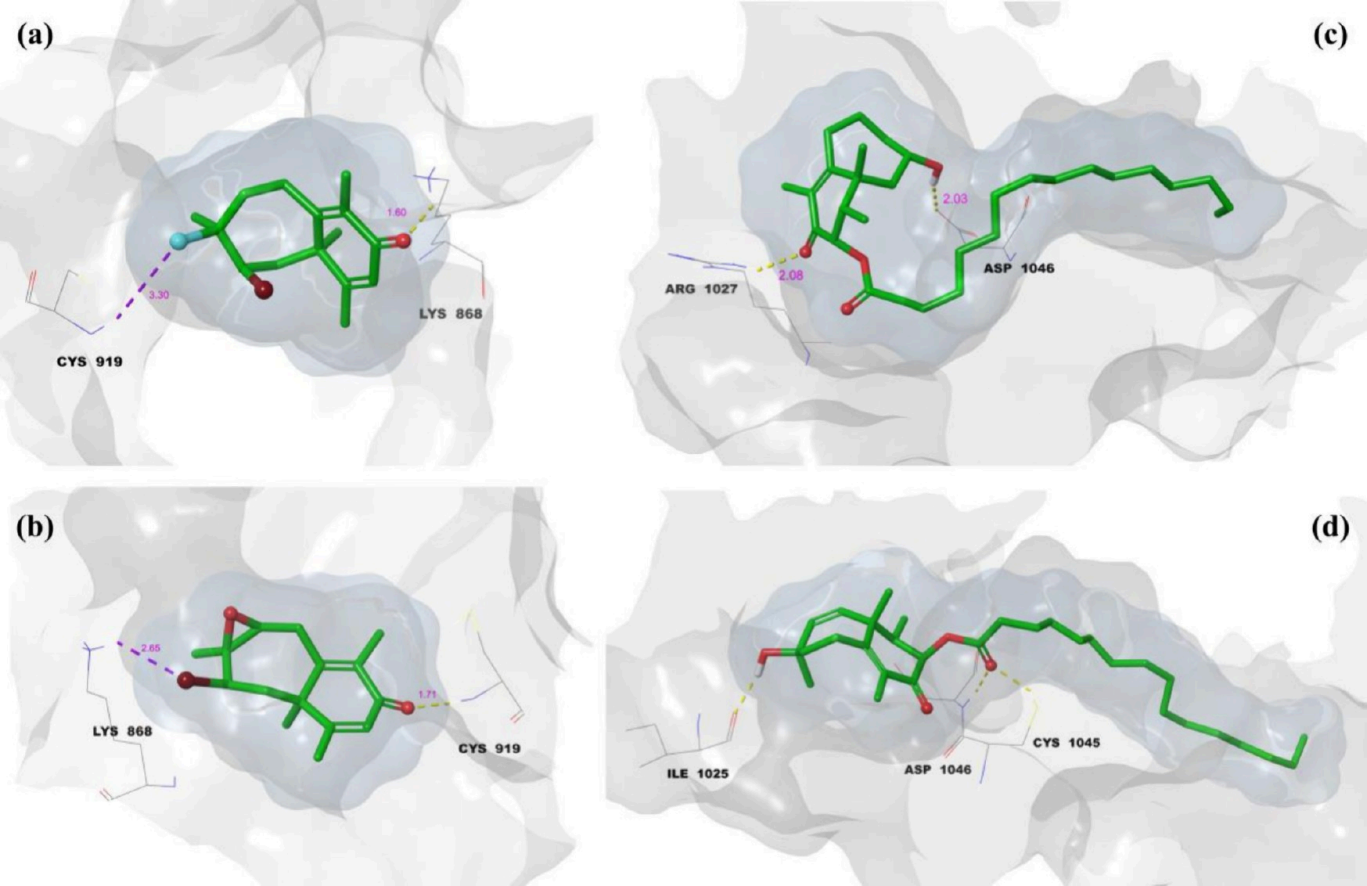
Molecular docking 3D LPI between isolate compounds and VEGFR2: (a) compound **1**, (b) compound **2**, (c) compound **3**, and (d) compound **4**.

In Figure 9a, the 1-VEGFR2 complex exhibits a hydrogen bond between the oxygen atom of the ketone carbonyl and Lys-868, along with a halogen bond formed by the chlorine atom with Cys-919. Similarly, Figure 9b shows that the 2-VEGFR2 complex features a hydrogen bond between the ketone carbonyl oxygen and Cys-919, while the bromine atom forms a halogen bond with Lys-868, mirroring a complementary interaction pattern with compound **1**. For compound **3**, depicted in Figure 9c, the oxygen atom of its α, β-unsaturated carbonyl group forms a hydrogen bond with Arg-1027, while the hydroxyl group establishes an additional hydrogen bond with Asp-1046. Finally, for compound **4**, shown in Figure 9d, the ester carbonyl oxygen atom engages in two hydrogen bonds with Asp-1046 and Cys-1047, whereas its hydroxyl group forms another hydrogen bond with Ile-1025.

In Figure 10a, compound **1** forms hydrogen and halogen bonds with lengths of 1.60 and 3.30 Å, respectively, with the ligand fully occupying the protein’s active site, as their surface binding areas perfectly overlap. Figure 10b shows compound **2** with hydrogen and halogen bond lengths of 1.71 and 2.65 Å, respectively, again demonstrating complete overlap of the ligand and protein surface binding areas. Figure 10c presents compound **3**, where two hydrogen bonds of 2.03 and 2.08 Å stabilize the complex. The 16-carbon side chain of the ligand facilitates free movement, allowing the ligand to fit completely into the binding site and form a more stable complex. Similarly, Figure 10d, for compound **4**, with hydrogen bond lengths of 1.83, 1.90, and 1.97 Å, shows the ligand achieving a complete fit in the protein’s binding site. Like compound **3**, the 16-carbon side chain aids in stabilizing the complex by ensuring proper accommodation of the ligand.

Molecular docking studies demonstrated that compound **3** exhibits the strongest binding affinity for VEGFR1, with the best docking score of −10.045 kcal/mol and an MM-GBSA binding energy of −92.97 kcal/mol, indicating potent inhibition. Compound **4** showed the highest docking score for VEGFR2 (−12.481 kcal/mol) and strong stability with MM-GBSA values of −88.34 kcal/mol for VEGFR2 and −72.94 kcal/mol for EGFR, positioning it as a promising multitarget inhibitor. Key amino acids, such as Asp-1040, Cys-1039, Lys-868, and Cys-919, were integral to stabilizing the ligand–protein complexes, with Asp-1040 and Cys-1039 playing central roles in VEGFR1 binding and Cys-919 and Lys-868 being critical in VEGFR2 interactions. The flexibility of the 16-carbon side chains in compounds **3** and **4** further enhanced binding stability, ensuring optimal accommodation within the receptors’ active sites. Overall, these results underscore the potential of these compounds as both effective single-target and multitarget inhibitors, with compound **3** emerging as a particularly promising candidate for VEGFR1 inhibition and compound **4** showing potential as a multitarget inhibitor.

#### 2.3.3 Molecular Docking Analysis on EGFR

The 2D and 3D LPI profiles of these compounds with EGFR are presented in Figures 11 and 12, respectively. Figure 11a illustrates the 2D LPI diagram of the 1-EGFR complex, where the ketone carbonyl group of compound 1 forms a single hydrogen bond with Cys-797. Similarly, in Figure 11b, the ketone carbonyl of compound 2 establishes a hydrogen bond with Met-793. Figure 11c represents the 2D LPI diagram of the 3-EGFR complex, which involves a total of three hydrogen bonds: one between the hydroxyl group and Asp-855, a second between the ester carbonyl and Phe-722, and a third between the ketone carbonyl and Ala-222. Likewise, compound 4 forms three hydrogen bonds within the active site of EGFR: the hydroxyl group interacts with Met-793, the ester carbonyl interacts with Ser-720, and the ketone carbonyl interacts with Cys-797 (Figure 11d). Overall, these observations reveal that Cys-797 and Met-793 are key conserved residues across multiple EGFR–ligand complexes, frequently engaging with the ketone and hydroxyl functional groups of the ligands.

**Figure 11:**
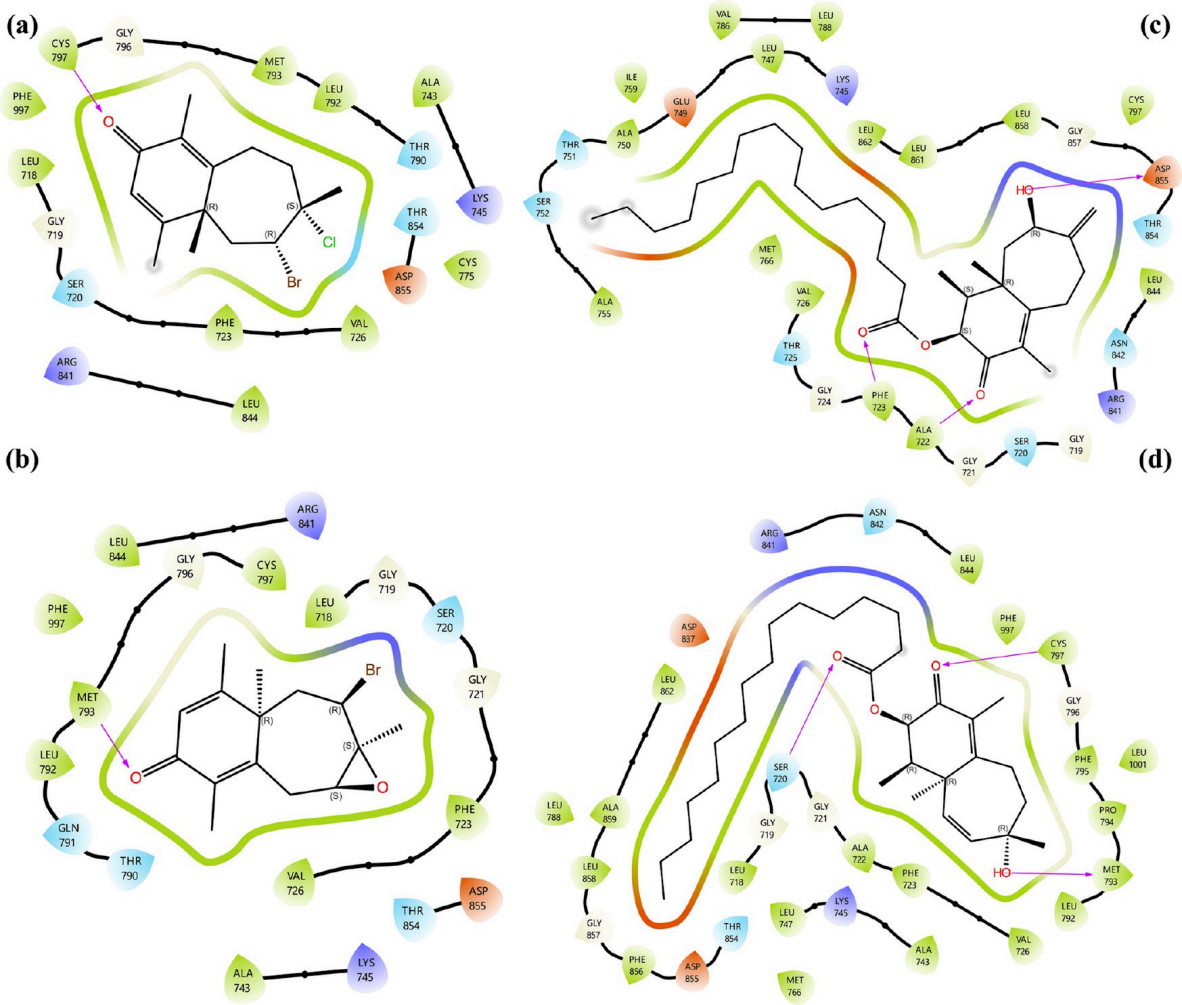
Molecular docking 2D LPI between isolated compounds and EGFR: (a) compound **1**, (b) compound **2**, (c) compound **3**, and (d) compound **4**.

**Figure 12:**
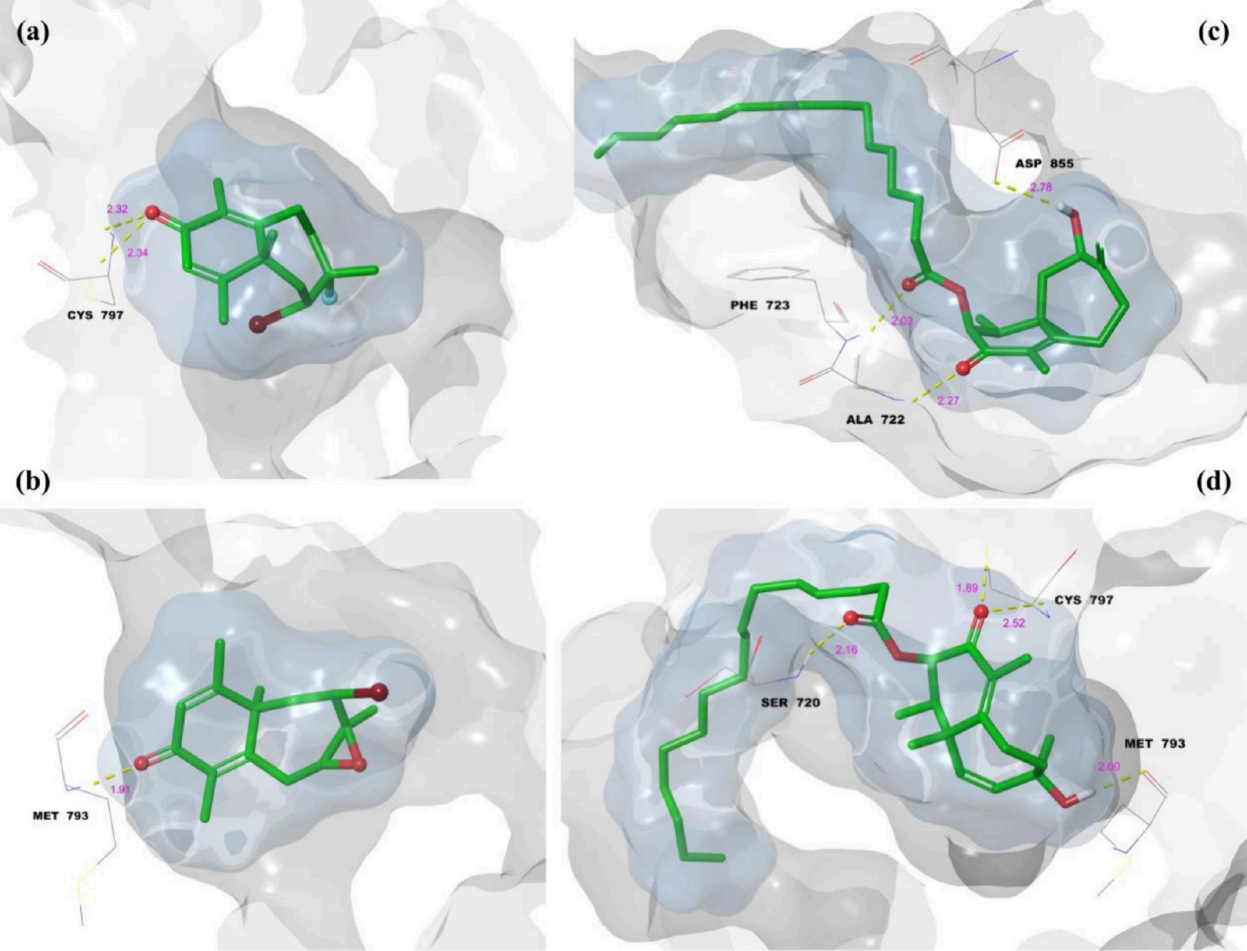
Molecular docking 3D LPI between isolate compounds and EGFR: (a) compound **1**, (b) compound **2**, (c) compound **3**, and (d) compound **4**.

Figure 12 presents the 3D LPI diagrams and binding modes of the EGFR complexes. In all four complexes, the ligand binding surface areas completely overlap with the corresponding protein binding surfaces, indicating that the molecules are well accommodated within the EGFR active site. The yellow dashes represent hydrogen bonds. In the 1-EGFR complex, the hydrogen bond lengths are 2.32 and 2.34 Å (Figure 12a). In the 2-EGFR complex, a single hydrogen bond with a length of 1.91 Å is observed (Figure 12b). For the 3-EGFR complex, the hydrogen bond lengths are 2.03, 2.27, and 2.78 Å (Figure 12c). Finally, in the 4–EGFR complex, four hydrogen bonds are observed with lengths of 1.89, 2.00, 2.16, and 2.52 Å (Figure 12d). In the 3D visualizations, it is noteworthy that in both the 1-EGFR and 4-EGFR complexes, Cys-797 forms two distinct hydrogen bonds, although these interactions are observed as single bonds in the corresponding 2D diagrams. This suggests that Cys-797 plays a consistent and critical role in stabilizing the ligand–EGFR interactions across multiple complexes.

### 2.4 Molecular Dynamics Simulations

In this study, MD simulations were performed for all four compounds against VEGFR1 and VEGFR2 proteins over a 100 ns time scale. Key LPIs, as well as RMSD, RMSF, and fractional interaction histograms, were analyzed in the MD simulation. Table 3 summarizes the RMSD (root mean square deviation) and RMSF (root mean square fluctuation) values for the protein Cα atoms and ligand atoms in all complexes. Among the tested compounds, compound **3** exhibited the most stable and compact binding with both VEGFR1 and VEGFR2, as reflected by its lower RMSD and RMSF values compared to those of other compounds. This indicates reduced structural deviation and flexibility during the simulation, consistent with a strong and stable inhibitory interaction. Additionally, the inclusion of EGFR in the MD analysis revealed that compound **2** formed the most stable EGFR complex, showing the lowest ligand RMSD (1.2 Å) and minimal protein fluctuations. This suggests that compound **2** maintains a highly stable conformation within the EGFR binding site, further supporting its potential as an EGFR inhibitor. Overall, these MD simulation results highlight that while compound **3** displays superior binding stability toward VEGFR1 and VEGFR2, compound **2** demonstrates the most favorable dynamic behavior in the EGFR complex.

**Table 3:** RMSD and RMSF Values of the VEGFR1-2 Complexes of the Title Compounds

	average RMSD (Å)			average RMSF (Å)	
complexes	protein Cα	ligand fit Protein	ligand fit Ligand	protein Cα	ligand
1-VEGFR1	2.4	11.5	0.5	1.6	3.5
2-VEGFR1	2.4	5.0	0.9	1.6	1.7
3-VEGFR1	2.4	**2.5**	1.5	0.8	2.0
4-VEGFR1	2.4	2.8	1.6	1.6	2.1
1-VEGFR2	2.2	4.5	0.4	1.5	0.9
2-VEGFR2	2.2	4.0	0.8	1.5	2.0
3-VEGFR2	2.2	**2.5**	2.0	0.8	1.0
4-VEGFR2	1.8	3.5	2.5	1.5	1.1
1-EGFR	1.8	3.0	0.2	0.9	1.2
2-EGFR	1.5	**1.2**	0.4	1.0	1.0
3-EGFR	1.8	2.0	1.1	1.2	1.2
4-EGFR	1.8	2.1	1.0	1.0	1.2

The 100 ns MD simulation analysis of the 3-VEGFR1 complex is given in Figure 13.

**Figure 13:**
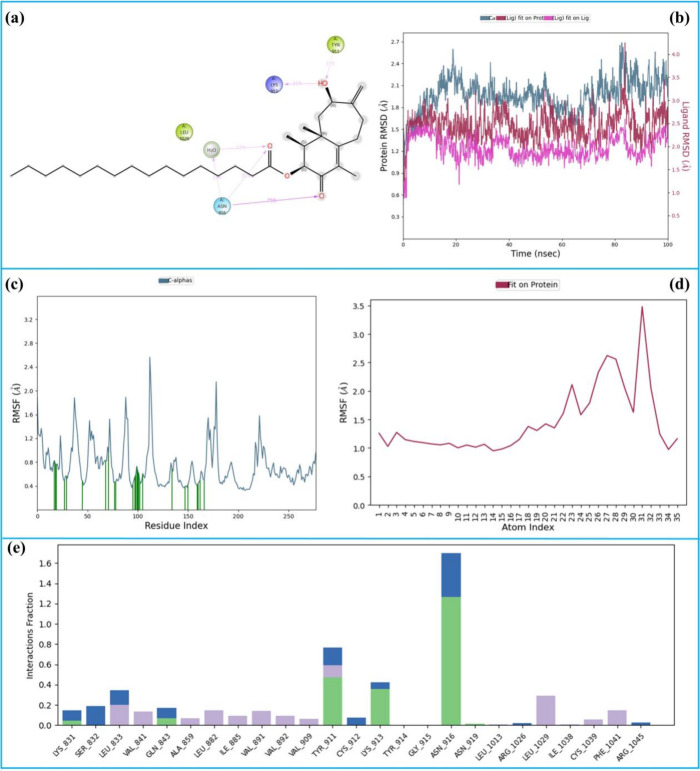
100 ns MD simulation analysis of the 3-VEGFR1 complex. (a) 2D key LPIs, (b) RMSD graphics, (c) RMSF of protein Cα, (d) RMSF of ligand, and (e) fractional interaction histogram.

Figure 13a illustrates the 2D key LPI observed during the simulation of the 3-VEGFR1 complex. The hydroxyl group formed hydrogen bonds with Lys-913 (35%) and Tyr-911 (31%), while the ester carbonyl established both a water-bridged hydrogen bond with Asn-916 (22%) and a direct hydrogen bond with the same residue (35%). Additionally, the ketone carbonyl formed a hydrogen bond with Asn-916 that persisted for 75% of the simulation time. These interactions emerged as the key stabilizing contacts within the complex. Figure 13b represents the RMSD of the protein and ligand atoms. The average RMSD of the protein Cα atoms was 2.4 Å (pale blue), while the ligand exhibited an average RMSD of 2.5 Å (red). The ligand’s deviation from its initial position was minimal, with an average of 1.5 Å, indicating stable binding throughout the simulation.

Figure 13c,d depicts the RMSF values for protein Cα atoms and ligand atoms, respectively. The protein’s average RMSF was 0.8 Å, reflecting very limited residue flexibility. The ligand’s RMSF averaged 2.0 Å, suggesting minor fluctuations in specific ligand regions. In Figure 13c, vertical green bars represent amino acid contacts with 26 total contacts identified, emphasizing the consistent interactions between the ligand and key residues.

Finally, Figure 13e shows the fractional interaction histogram, where each interaction type is color-coded: green for hydrogen bonds, blue for water-bridged hydrogen bonds, and purple for hydrophobic interactions. In a simulation, a single amino acid can interact with multiple functional groups of the ligand, and similarly, a single functional group can interact with multiple amino acids. These interactions are cumulatively represented in a fractal interaction histogram. The most significant interactions were observed with Asn-916, Tyr-911, and Lys-923, which played central roles in ligand binding and contributed to the stability and specificity of the complex. Overall, the simulation results underscore the importance of these residues in stabilizing the 3-VEGFR1 complex, supported by the stable binding profile of the ligand.

The 100 ns MD simulation analysis of the 3-VEGFR2 complex is given in Figure 14. Figure 14a illustrates the two-dimensional key LPI observed during the simulation of the 3-VEGFR2 complex. The hydroxyl group of compound **3** formed hydrogen bonds with Lys-868 (76%) and Asp-1046 (98%) while also engaging in a water-bridged hydrogen bond with Leu-1049 (22%). On the other hand, the ketone carbonyl formed a hydrogen bond with Arg-1027 (78%) and a water-bridged hydrogen bond with His-1026 (22%). These persistent interactions emerged as the key stabilizing contacts within the complex. Figure 14b presents the RMSD values of the protein and ligand atoms. The average RMSD of the protein Cα atoms was 2.2 Å (pale blue), indicating a stable protein structure during the simulation. The ligand exhibited an average RMSD of 2.5 Å (red) with a minimal deviation of 2.0 Å from its initial position, demonstrating consistent binding within the active site.

**Figure 14:**
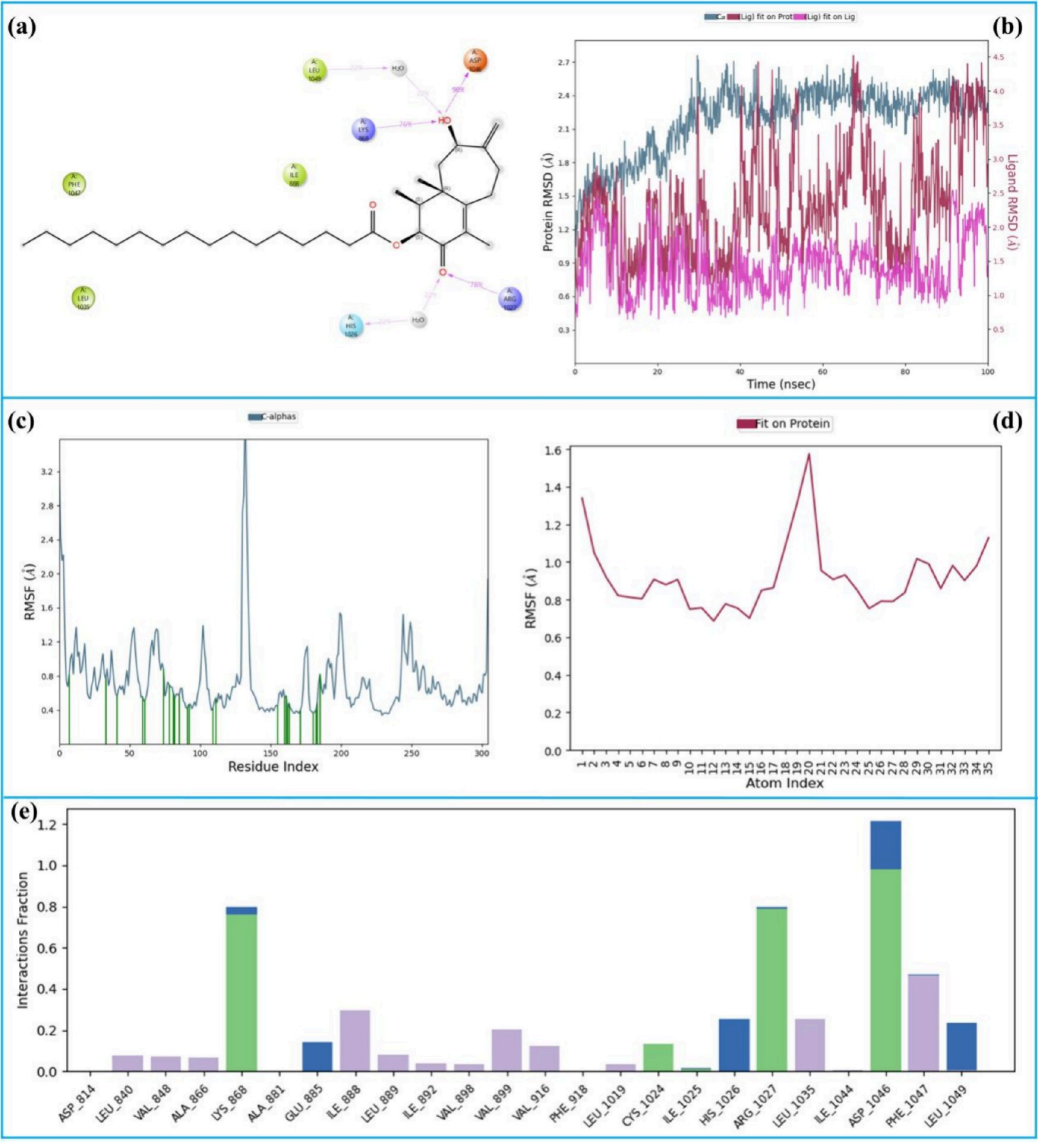
100 ns MD simulation analysis of the 3-VEGFR2 complex. (a) 2D key LPI, (b) RMSD graphics, (c) RMSF of protein Cα, (d) RMSF of ligand, and (e) fractional interaction histogram.

Figure 14c,d shows the RMSF values for the protein Cα atoms and ligand atoms, respectively. The average RMSF of the protein Cα atoms was 0.8 Å, indicating very limited flexibility in the protein’s structure. The ligand’s RMSF was 1.8 Å, suggesting only minor fluctuations and stable binding behavior. In Figure 14c, vertical green bars mark the amino acid contacts with a total of 23 contacts identified, reflecting robust interactions between the ligand and the protein. Figure 14e represents the fractional interaction histogram, which consolidates all LPIs. The histogram reveals that Asp-1046, Arg-1027, Lys-868, and Phe-1047 exhibited the highest interaction frequencies, reaffirming their critical roles in ligand stabilization. Overall, the simulation results demonstrate that the 3-VEGFR2 complex is highly stable, with these key residues maintaining strong hydrogen bonding and interaction networks. The ligand’s stable binding and minimal fluctuations further confirm its potential as a VEGFR2 inhibitor.

The 100 ns MD simulation analysis of the 2-EGFR complex is given in Figure 15. Figure 15a shows the 2D LPI diagram of the 2-EGFR complex throughout the MD simulation. The ketone carbonyl group of the ligand maintained a hydrogen bond with Met-793 for approximately 99% of the simulation time, indicating a highly stable and strong interaction. Additionally, the epoxide oxygen formed two distinct water-bridged hydrogen bond interactions with Asp-855 and Arg-841, observed for 58 and 44% of the simulation time, respectively.

**Figure 15:**
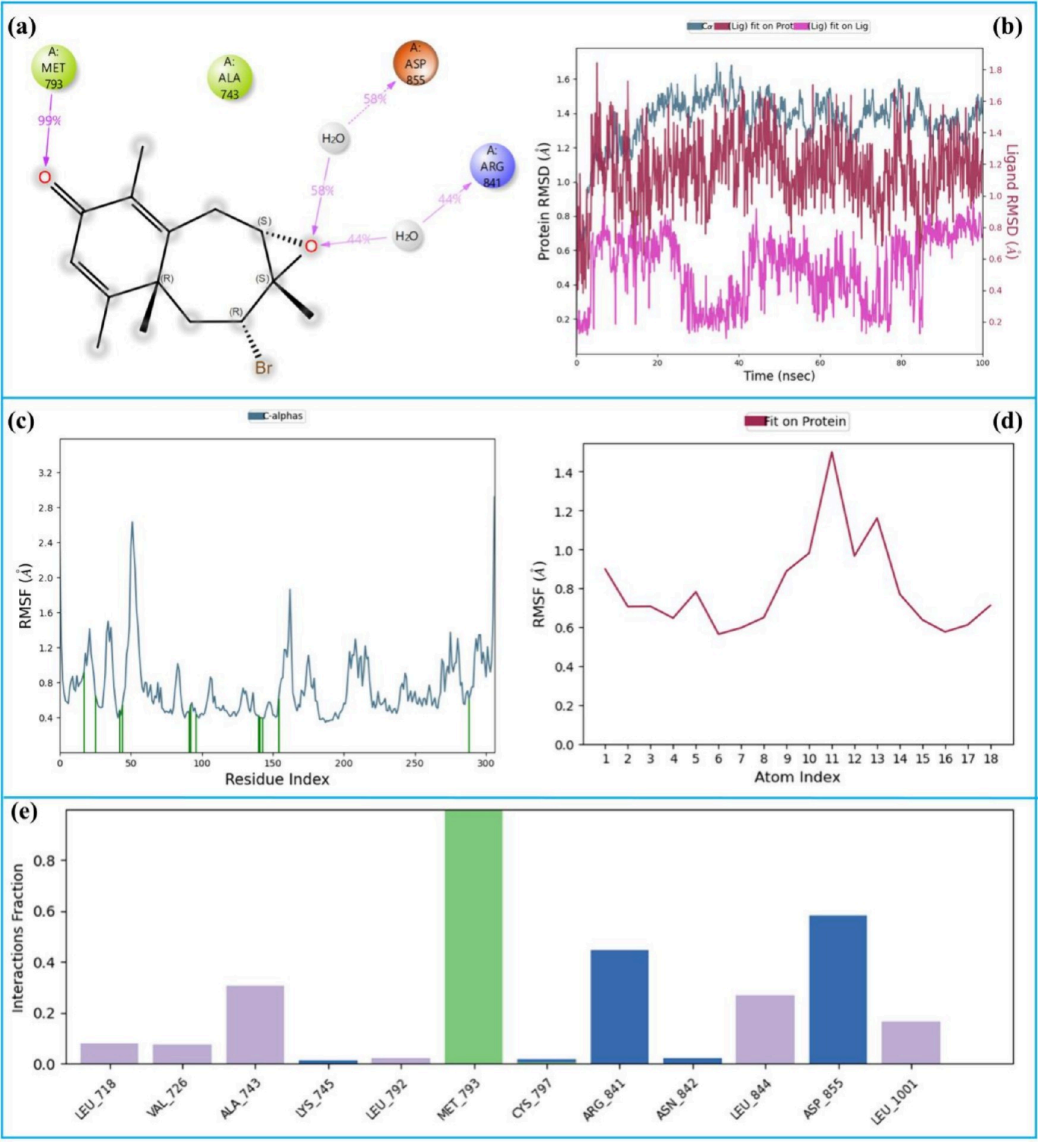
100 ns MD simulation analysis of the 2-EGFR complex. (a) 2D key LPI, (b) RMSD graphics, (c) RMSF of Protein Cα, (d) RMSF of ligand, and (e) fractional interaction histogram.

The ligand’s RMSD relative to its initial binding position remained around 0.4 Å (pink), confirming its stable orientation within the EGFR active site throughout the 100 ns simulation. Figure 15c,d displays the RMSF profiles for the protein and ligand atoms, respectively, with average RMSF values of 1.0 Å for both, suggesting minimal structural fluctuations and the overall stability of the complex. Finally, the fractional interaction histogram (Figure 15e) indicates that Met-793 is the most dominant interacting residue, followed by Arg-841 and Asp-855. These results highlight the critical role of Met-793 as a key anchoring residue in stabilizing the ligand–EGFR complex, supported by additional interactions with Arg-841 and Asp-855.

MD simulations demonstrated that compound **3** is a highly stable dual VEGFR1/VEGFR2 inhibitor with strong and persistent binding. In VEGFR1, key contacts included Lys-913, Tyr-911, and Asn-916, while in VEGFR2, Asp-1046, Arg-1027, Lys-868, and Phe-1047 dominated the interaction network. RMSD and RMSF analyses confirmed minimal ligand deviation and limited protein flexibility, and fractional interaction histograms highlighted these residues as being critical for stabilization. In addition, the inclusion of EGFR in the MD study revealed that compound **2** formed the most stable complex among the tested ligands, maintaining a strong and consistent hydrogen bond with Met-793 for 99% of the simulation time. Water-mediated interactions with Asp-855 and Arg-841 further contributed to the overall binding stability. RMSD and RMSF values for the 2-EGFR complex (1.4 and 1.0 Å, respectively) indicated exceptional conformational stability throughout the simulation. Overall, these findings suggest that compound **3** exhibits potent dual inhibition against VEGFR1 and VEGFR2, while compound **2** shows strong and selective affinity toward EGFR. Together, they highlight the potential of these molecules as complementary anticancer agents targeting angiogenesis and EGFR-driven signaling pathways. The 100 ns MD simulation analysis of other complexes is given in Figures S39–S47.

The dynamic behavior and stability of the selected protein–ligand complexes during the 100 ns MD simulations were further analyzed in terms of hydrogen bond timelines and structural parameters, as illustrated in Figure 16.

**Figure 16:**
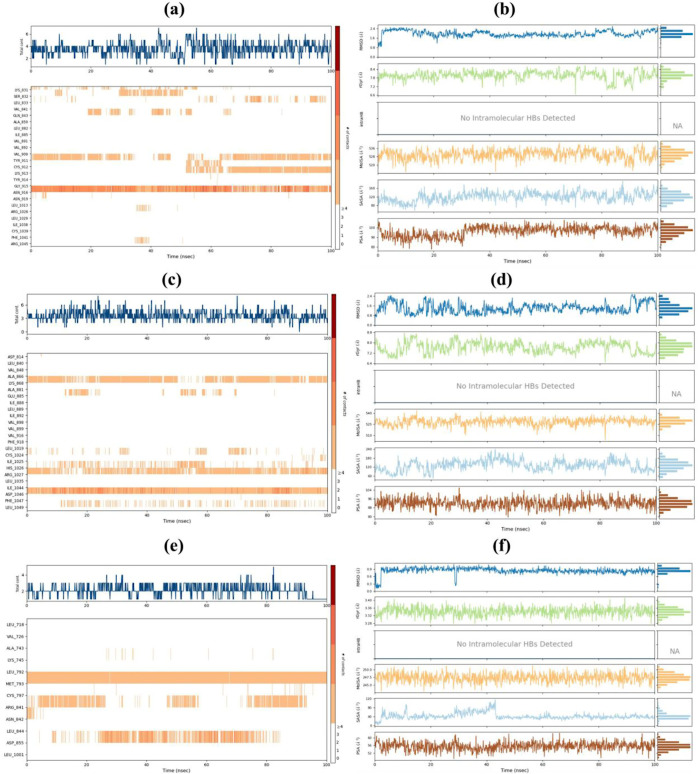
MD simulation, hydrogen bond timeline depictions of 3-VEGFR1 (a), 3-VEGFR2 (c), and 2-EGFR (e). RMSD, rGyr, MoISA, SASA, and PSA values of 3-VEGFR1 (b), 3-VEGFR2 (d), and 2-EGFR (f).

Figure 16a shows that in the 3-VEGFR1 complex, Asn-916 maintained a stable hydrogen bond interaction throughout the entire simulation period. Tyr-911 also preserved its hydrogen bond interaction except between 40–60 ns, while Lys-913 formed hydrogen bonds mainly during the second half of the simulation, which contributed to the overall stability of the complex. The radius of gyration (rGyr) values ranged between 6.6 and 8.4 Å, indicating compact structural behavior. The molecular surface area (MolSA) values remained between 520 and 536 Å^2^, while the solvent-accessible surface area (SASA) and polar surface area (PSA) fluctuated between 80–160 Å^2^ and 80–100 Å^2^, respectively. These parameters collectively reflect the compactness, solvent exposure, and polarity balance of the protein–ligand complex during the simulation. In Figure 16c, the 3-VEGFR2 complex shows persistent hydrogen bond interactions with Asp-1046, Arg-1027, and Lys-868 throughout the entire simulation, identifying these residues as key contributors to the complex stability. As shown in Figure 16d, the rGyr values ranged from 6.4 to 8.8 Å, while MolSA, SASA, and PSA varied between 510–540 Å^2^, 60–240 Å^2^, and 80–104 Å^2^, respectively. These stable surface and compactness profiles confirm the strong and consistent binding of compound **3** within the VEGFR2 active site. Figure 16e demonstrates that in the EGFR complex, Met-793 maintained a continuous hydrogen bond interaction for the entire simulation period. Arg-841 showed intermittent hydrogen bonding during approximately two-thirds of the simulation, whereas Asp-855 engaged in hydrogen bond interactions primarily between 20–80 ns. As illustrated in Figure 16f, the rGyr values of the complex remained within 3.28–3.40 Å, indicating a highly compact and stable structure. MolSA, SASA, and PSA values were measured in the ranges of 245–250 Å^2^, 0–120 Å^2^, and 52–60 Å^2^, respectively, further supporting the overall conformational stability and effective ligand accommodation within the EGFR binding pocket.

### 2.5 ADME Prediction

Assessment of the absorption, distribution, metabolism, and excretion (ADME) properties is a crucial step in evaluating the drug-likeness and pharmacokinetic potential of novel compounds. In this study, in silico ADME predictions were performed for the isolated perforane-type sesquiterpenes, and the results are summarized in Table 4. These analyses provide valuable insights into the pharmacological suitability of the compounds, complementing experimental cytotoxicity and computational docking studies, and help identify promising candidates for further preclinical development.

**Table 4:** ADME Prediction of Isolated Compounds

descriptors^a^	1	2	3	4	reference values
mol MW	331.67	311.21	488.74	488.74	130 to 725
donor HB	0	0	1	1	0 to 6
accept HB	2	4	6	5	2 to 20
QPlogPo/w	4.104	2.792	7.591	7.953	–2 to 6.5
QPlogS	–4.742	–3.273	–9.383	–9.438	–6.5 to 0.5
QPPCaco	3646	3432	971	1095	<25 poor, >500 great
QPlogBB	0.418	0.340	–1.720	–1.615	–3 to 1.2
QPPMDCK	6162	4213	479	545	<25 poor, >500 great
% human oral abs.	100	100	100	100	>80 high, <25 poor
Lipinski rule of five	0	0	1	1	Max 4
Jorgensen rule of three	0	0	1	1	Max 3

a The results of in silico ADME predictions.

The *in silico* ADME analysis of the isolated perforane-type sesquiterpenes (Table 4) revealed favorable pharmacokinetic profiles for all compounds. Molecular weights of the compounds ranged from 311 to 488 Da, within the acceptable range for drug-likeness. Hydrogen bond donors were low (0–1), and acceptors ranged from 2 to 6, indicating the potential for good membrane permeability. Compounds **1** and **2** showed moderate lipophilicity (QPlog*P*_o/w_ 4.104 and 2.792), while compounds **3** and **4** were more lipophilic (7.591 and 7.953), slightly exceeding the recommended upper limit. Solubility (QPlog*S*) was adequate for compounds **1** and **2** (−4.742 and −3.273), whereas compounds **3** and **4** displayed low aqueous solubility (−9.383 and −9.438), which may require formulation strategies. Both Caco-2 and MDCK permeability predictions were high for compounds **1** and **2**, suggesting excellent intestinal absorption, whereas compounds **3** and **4** showed moderate permeability.

Predicted human oral absorption was 100% for all compounds, and blood–brain barrier penetration (QPlogBB) values indicated limited CNS exposure for compounds **3** and **4**. Overall, Lipinski and Jorgensen rules were satisfied for compounds **1** and **2**, while compounds **3** and **4** violated one rule each, reflecting their higher molecular weight and lipophilicity. These results indicate that all four sesquiterpenes have favorable ADME properties, with compounds **1** and **2**, exhibiting the best overall drug-likeness and compounds **3** and **4** remaining promising candidates with minor considerations for solubility and permeability.

## 3 Conclusions

In conclusion, four new perforane-type sesquiterpenes **(1–4)** were successfully isolated and structurally elucidated from *Laurencia obtusa*, and all demonstrated significant cytotoxicity against A549 lung adenocarcinoma cells, with compounds **1** and **2** showing the highest potency and selectivity indices, surpassing the reference drug doxorubicin. Molecular docking and MM-GBSA analyses revealed that compounds **3** and **4** possess the strongest binding affinities, with compound **3** emerging as the most promising VEGFR1 inhibitor and a highly stable dual VEGFR1/VEGFR2 binder. In contrast, compound **4** exhibited potential as a multitarget inhibitor, particularly for VEGFR2 and EGFR. Key residues, including Asp-1040, Cys-1039, Lys-868, Asp-1046, Arg-1027, Tyr-911, and Asn-916, were critical in stabilizing the ligand–protein complexes, as confirmed by MD simulations, RMSD/RMSF analyses, and fractional interaction histograms. ADMET predictions further indicated favorable pharmacokinetic profiles and acceptable toxicity for all of the compounds. Overall, these findings highlight the therapeutic potential of these perforane-type sesquiterpenes as selective, potent, and multitarget anticancer agents targeting angiogenesis and lung cancer pathways.

## 4 Experimental Section

### 4.1 General Experimental Procedures

Optical rotations were measured on an AA-65 Series Automatic Polarimeter. The absorbance and IR spectra were obtained on a HITACHI U-2900 UV–Vis spectrophotometer and a Bruker ALPHA II FTIR spectrometer, respectively. NMR spectra were recorded with chloroform as an internal standard (δ_C_ 77.0, δ_H_ 7.26) on a Bruker 500 MHz spectrometer. High-resolution electrospray ionization mass spectra (HR-ESIMS) of the isolated compounds were obtained using a Thermo Q Exactive mass spectrometer (Thermo Scientific).

### 4.2 Alga Material

The red alga *Laurencia obtusa* (Hudson) J. V. Lamouroux was collected from the Aegean Sea, coast of Çanakkale (Yapıldakaltı), Türkiye, in July 2018 at a depth of 0.5–1 m by SCUBA diving. A voucher specimen (No. E15) is deposited at the Faculty of Aquatic Sciences and Fisheries, Akdeniz University, Antalya, Türkiye, where its taxonomic identification was confirmed by Emine ükran Okudan (Figure 17).

**Figure 17:**
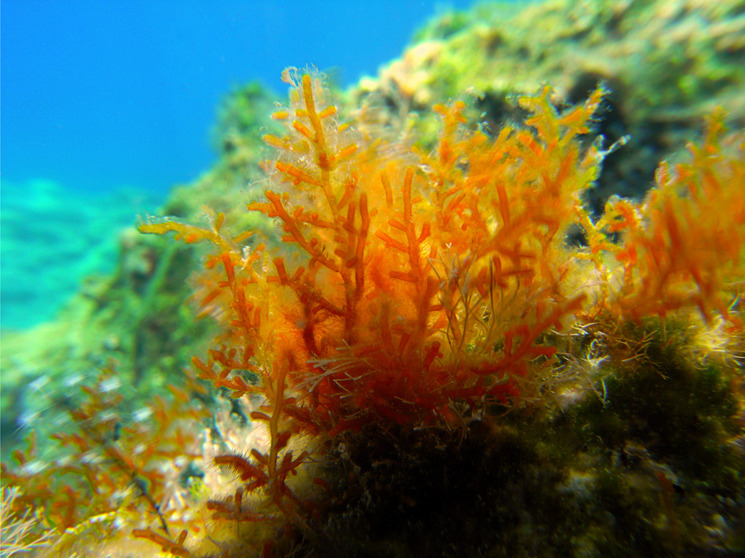
*Laurencia obtusa* (photograph courtesy of Dr.Emine Şükran Okudan).

### 4.3 Extraction and Isolation

The dried and powdered alga (281.7 g) was exhaustively extracted at room temperature with dichloromethane (DCM)–methanol (MeOH) (1:1, v/v; 1 L) for 48 h. The extraction was repeated four times, and the combined extracts were filtered and concentrated under reduced pressure to afford a dark-green crude extract (15.5 g, 5.5% yield).

The crude extract was subjected to vacuum liquid chromatography (VLC) on silica gel (type H, 10–40 μm) and eluted successively with solvent mixtures of increasing polarity. The following solvent systems were used: 100% hexane (A), 25% EtOAc/hexane (B); 50% EtOAc/hexane (C); 75% EtOAc/hexane (D); 100% EtOAc (E); 75% EtOAc/MeOH (F); 50% EtOAc/MeOH (G); 25% EtOAc/MeOH (H); 100% MeOH (I). Nine fractions (A–I) were thus obtained. Each fraction was monitored by TLC under UV light (254 and 365 nm) and visualized by spraying with 10% aqueous ceric sulfate reagent, followed by heating at 100 °C.

Fraction **C** (3.1 g), eluted with 50% EtOAc/hexane in VLC, was further separated by silica gel column chromatography under the same gradient system (100% hexane to 100% EtOAc, followed by 5% MeOH/EtOAc). A total of 637 subfractions were obtained and combined into 25 fractions according to identical *R*_f_ values.

The combined fractions of F11–F14 eluted with 5% EtOAc/hexane were rechromatographed on a smaller silica column (1 × 40 cm) using fine polarity steps (1–5% EtOAc/hexane), yielding two pure compounds, **1** (8.4 mg) and **2** (6.8 mg). A subsequent portion, eluted with 6% EtOAc/hexane, contained two coeluting compounds and was further purified by preparative TLC using 10% EtOAc/hexane as the developing solvent to afford compounds **3** (6.3 mg) and **4** (19.6 mg).

#### 4.3.1 Obtusadienone A (**1**)

White amorphous; [α]_D_^25^ −13.48 (*c* 0.002, MeOH); IR *v*_max_/cm^–1^: 3434, 2921, 2851, 1729, 1450,1382, 830, 589; for ^1^H NMR (CDCl_3_, 500 MHz) and ^13^C NMR (CDCl_3_, 125 MHz) spectral data, see Tables S1; HR-ESI-MS *m*/*z* 331.04529 (C_15_H_20_^79^Br^35^ClO) and 333.04294 (C_15_H_20_^81^Br^35^ClO) (calcd. for 331.603861 and 332.03656). qHNMR_purity_: >95% (Table S5).

#### 4.3.2 Obtusadienone B (**2**)

White amorphous; [α]_D_^25^ −18.57 (*c* 0.003, MeOH); IR *v*_max_/cm^–1^: 3696, 2922, 2850, 1658, 1612, 1278, 713; for ^1^H NMR (CDCl_3_, 500 MHz) and ^13^C NMR (CDCl_3_, 125 MHz) spectral data, see Tables S2; HR-ESI-MS *m*/*z* 311.16873 (C_15_H_19_BrO_2_) (calcd for 310.05684). qHNMR_purity_: >95% (Table S6).

#### 4.3.3 Obtusaenone A (**3**)

White amorphous; [α]_D_^25^ +20.63 (*c* 0.001, MeOH); IR *v*_max_/cm^–1^: 3570, 2918, 2849, 1665, 1736, 1462, 1032; for ^1^H NMR (CDCl_3_, 500 MHz) and ^13^C NMR (CDCl_3_, 125 MHz) spectral data, see Tables S3; HR-ESI-MS [M + Na]^+^ observed at *m*/*z* 511.37500 agreed with the molecular formula C_31_H_52_O_4_Na. qHNMR_purity_: >96% (Table S7).

#### 4.3.4 Obtusaenone B (**4**)

White amorphous; [α]_D_^25^ +42.89 (c 0.009, MeOH); IR *v*_max_/cm^–1^: 3467, 2925, 2853, 1740, 1688, 1460, 1380, 747; for ^1^H NMR (CDCl_3_, 500 MHz) and ^13^C NMR (CDCl_3_, 125 MHz) spectral data, see Tables S4; HR-ESI-MS [M + Na]^+^ observed at *m*/*z* 511.37500 agreed with the molecular formula C_31_H_52_O_4_Na. qHNMR_purity_: >95% (Table S8).

### 4.4 Cell Culture

A549 and BEAS cells were cultured in DMEM/F12 and DMEM, respectively, both supplemented with 10% FBS and 100 units/mL of penicillin–streptomycin. The cultures were maintained at 37 °C in a humidified incubator with 5% CO_2_. When the cells reached 80% confluence, they were detached using 0.25% trypsin-EDTA. Subsequently, for further experiments, the cells were resuspended in the growth medium after being collected and centrifuged.^42,41^

### 4.5 MTT Assay

The MTT (3-(4,5-dimethylthiazol-2-yl) 2,5-diphenyltetrazolium bromide) assay was employed to assess the cytotoxicity of the test compounds. Six different concentrations (100, 50, 25, 12.5, 6.25, and 3.12 μM) of each compound were tested on each cell line. Specifically, 5 × 10 cells were seeded in a flat-bottomed 96-well plate with the growth medium. After a 24 h incubation period, the cells were exposed to increasing concentrations of the compounds for 24 h before conducting the assay. For termination of the assay, 10 μL of MTT solution (5 mg/mL in PBS) was added to each well. After 3 h of incubation at 37 °C, 100 μL of DMSO was added to each well and absorbance was read at 540 nm using an ELISA microplate reader. All experiments were performed in triplicate, and the results were presented as the mean ± standard deviation. A concentration-dependent graph was generated by comparing data collected from at least three measurements of each substance, and the relative % cell viability was determined. To assess the cytotoxic impact of the compounds on the cell viability, untreated cells were considered 100% viable. Cell viability was calculated using the following formula: % Cell viability = (Sample/Control) × 100.^41,44^

### 4.6 Computational Studies

Molecular docking and dynamics simulations were conducted using Schrödinger Molecular Modeling Software (2025-1) with the Maestro interface (version 14.3) and Desmond (D. E. Shaw Research 2024-4). Protein and ligand preparation followed previously published methods.^45^ For ligand preparation, the LigPrep module of Schrödinger was used to generate all possible ionization and tautomeric states at pH 7.4, ensuring the correct geometry and ionization states for docking simulations. Ligands were then minimized using the OPLS4 force field before docking. For protein preparation, the Protein Preparation Wizard in Schrödinger was employed to prepare the target enzymes VEGFR1 (PDB ID: 3HNG), VEGFR2 (PDB ID: 4ASE), and EGFR (PDB ID: 3W2S), which were retrieved from the Protein Data Bank. The protein structures were optimized by adding missing hydrogens, assigning correct protonation states, and performing energy minimization to remove steric clashes.^46^

Glide docking and induced fit docking (IFD) were performed to dock ligands into the target receptors. In the IFD procedure, 20 docking poses were generated for each ligand using Glide XP (extra precision) with a box size of 20 × 20 × 20 Å to encompass the entire binding site. The best docking poses were selected based on their IFD docking scores, and the optimal interactions were further refined. For the IFD protocol, the receptor was flexibly allowed to adjust to ligand binding, and the ligand was docked iteratively. This approach ensures that both the protein and ligand conformations are well-optimized to reflect the dynamic nature of the binding process.^42^ Prime MM-GBSA analysis was used to estimate binding free energies for the protein–ligand complexes using the VSGB solvation model. This calculation provides insights into the strength and nature of the binding interactions, incorporating both electrostatic and van der Waals contributions.^47^

MD simulations were performed using Desmond, where the protein–ligand complex was solvated using the TIP4P water model with Na^+^ and Cl^–^ ions added to neutralize the system. The simulation box size was set to 10 × 10 × 10 Å, providing sufficient space for protein–ligand interactions and solvation. The system was first minimized to remove any bad contacts, followed by an equilibration phase in the NPT ensemble (constant pressure and temperature). The production run was conducted for 100 ns at 300 K and 1 atm. RMSD values for both the protein backbone and ligand atoms were monitored to assess the stability of the complex throughout the simulation. Additionally, interactions such as hydrogen bonds, hydrophobic contacts, and salt bridges were analyzed to evaluate the stability and strength of the protein–ligand complex during the simulation. These steps ensured accurate assessment of ligand binding and complex stability in a dynamic environment.^48–50^

## Supplementary Material



## Notes

NMR, UV, IR, and HRMS spectra of the isolated compounds; additional data of molecular dynamic simulations and validation images of molecular docking studies (PDF)

## References

[1] Guiry, M. D. ; Guiry, G. M. AlgaeBase; World-wide electronic publication, National University of Ireland: Galway, 2022. https://www.algaebase.org.

[2] Suzuki M., Vairappan C. (2005). Halogenated
secondary metabolites from Japanese species
of the red algal genus Laurencia (Rhodomelaceae, Ceramiales). Curr. Top. Phytochem..

[3] Suzuki M., Takahashi Y., Nakano S., Abe T., Masuda M., Ohnishi T., Noya Y., Seki K. (2009). An experimental approach
to study the biosynthesis of brominated metabolites by the red algal
genus Laurencia. Phytochemistry.

[4] Wang B. G., Gloer J. B., Ji N. Y., Zhao J. C. (2013). Halogenated organic
molecules of Rhodomelaceae origin: chemistry and biology. Chem. Rev..

[5] Harizani, M. ; Ioannou, E. ; Roussis, V. , The Laurencia Paradox: An Endless Source of Chemodiversity, in: Kinghorn, A. D. ; Falk, H. ; Gibbons, S. ; Kobayashi, J. I. (Eds.), Progress in the Chemistry of Organic Natural Products; Springer International Publishing: Cham, 2016, 102, 91–252. 10.1007/978-3-319-33172-0_2 27380407

[6] Al-Massarani S. M. (2014). Phytochemical
and biological properties of sesquiterpene constituents from the marine
red seaweed Laurencia: A review. Nat. Prod.
Chem. Res..

[7] Cikoš A.-M., Jurin M., Čož-Rakovac R., Gašo-Sokač D., Jokić S., Jerković I. (2021). Update on sesquiterpenes from red
macroalgae of the Laurencia genus and their biological activities
(2015–2020). Algal Research.

[8] Kamada T., Phan C.-S., Sien V.S.-T., Vairappan C. S. (2018). Halogenated
chamigrane sesquiterpenes from Bornean Laurencia majuscula. Journal of Applied Phycology.

[9] Kamada T., Phan C.-S., Okino T., Vairappan C. S. (2019). Cytotoxicity
and Antibacterial Potential of Halogenated Chamigrenes from Malaysian
Red Alga, Laurencia majuscula. Planta Medica
International Open.

[10] Kladi M., Xenaki H., Vagias C., Papazafiri P., Roussis V. (2006). New cytotoxic sesquiterpenes from
the red algae Laurencia
obtusa and Laurencia microcladia. Tetrahedron.

[11] Kamada T., Vairappan C. S. (2013). New bioactive secondary metabolites from Bornean red
alga, Laurencia similis (Ceramiales). Nat. Prod.
Commun..

[12] Zaleta-Pinet D. A., Holland I. P., Muñoz-Ochoa M., Murillo-Alvarez J. I., Sakoff J. A., van Altena I. A., McCluskey A. (2014). Cytotoxic
compounds from Laurencia pacifica. Org. Med.
Chem. Lett..

[13] Alorfi H. S., Ghandourah M. A., Turki A. J. (2020). Cytotoxic effect of acetogenins and
sesquiterpenes obtained from the Red alga Laurencia majuscula. Tropical Journal of Pharmaceutical Research.

[14] Wijesinghe W., Kim E.-A., Kang M.-C., Lee W.-W., Lee H.-S., Vairappan C. S., Jeon Y.-J. (2014). Assessment of anti-inflammatory effect
of 5β-hydroxypalisadin B isolated from red seaweed Laurencia
snackeyi in zebrafish embryo in vivo model. Environmental toxicology and pharmacology.

[15] Vairappan C. S., Kamada T., Lee W.-W., Jeon Y.-J. (2013). Anti-inflammatory
activity of halogenated secondary metabolites of Laurencia snackeyi
(Weber-van Bosse) Masuda in LPS-stimulated RAW 264.7 macrophages. Journal of applied phycology.

[16] Chen J.-Y., Huang C.-Y., Lin Y.-S., Hwang T.-L., Wang W.-L., Chiou S.-F., Sheu J.-H. (2016). halogenated
sesquiterpenoids from
the red alga Laurencia tristicha collected in Taiwan. J. Nat. Prod..

[17] Kamada T., Phan C.-S., Vairappan C. S. (2019). New anti-bacterial
halogenated tricyclic
sesquiterpenes from Bornean Laurencia majuscula (Harvey) Lucas. Natural product research.

[18] Kamada T., Vairappan C. S. (2015). New laurene-type
sesquiterpene from Bornean Laurencia
nangii. Nat. Prod. Commun..

[19] Bawakid N. O., Alarif W. M., Alorfi H. S., Al-Footy K. O., Alburae N. A., Ghandourah M. A., Al-Lihaibi S. S., Abdul-Hameed Z. H. (2017). Antimicrobial
sesquiterpenoids from Laurencia obtusa Lamouroux. Open Chemistry.

[20] Kamada T., Vairappan C. S. (2017). Non-halogenated new sesquiterpenes from Bornean Laurencia
snackeyi. Natural Product Research.

[21] Hu Z.-B., Yu X.-Q., Wang B., Liu A.-H., Zhao T.-S., Guo Y.-W., Huang H.-L., Mao S.-C. (2020). Structurally diverse
halosesquiterpenoids from the red alga Laurencia composita Yamada. Fitoterapia.

[22] Ventura T. L. B., da Silva Machado F. L., de Araujo M. H., de Souza Gestinari L. M., Kaiser C. R., de Assis
Esteves F., Lasunskaia E. B., Soares A. R., Muzitano M. F. (2015). Nitric
oxide production inhibition and anti-mycobacterial activity of extracts
and halogenated sesquiterpenes from the Brazilian red alga laurencia
dendroidea J. Agardh. Pharmacogn. Mag..

[23] Alarif W. M., Al-Footy K. O., Zubair M. S., Halid PH M., Ghandourah M. A., Basaif S. A., Al-Lihaibi S. S., Ayyad S.-E. N., Badria F. A. (2016). The role
of new eudesmane-type sesquiterpenoid and known eudesmane derivatives
from the red alga Laurencia obtusa as potential antifungal–antitumour
agents. Natural product research.

[24] Yu X.-Q., Jiang C.-S., Zhang Y., Sun P., Kurtan T., Mandi A., Li X.-L., Yao L.-G., Liu A.-H., Wang B. (2017). Compositacins
A–K: Bioactive chamigrane-type halosesquiterpenoids
from the red alga Laurencia composita Yamada. Phytochemistry.

[25] García-Davis S., Sifaoui I., Reyes-Batlle M., Viveros-Valdez E., Piñero J. E., Lorenzo-Morales J., Fernández J. J., Díaz-Marrero A.
R. (2018). Anti-Acanthamoeba activity
of brominated
sesquiterpenes from Laurencia johnstonii. Mar
Drugs.

[26] Arberas-Jiménez I., García-Davis S., Rizo-Liendo A., Sifaoui I., Reyes-Batlle M., Chiboub O., Rodríguez-Expósito R. L., Díaz-Marrero A. R., Piñero J. E., Fernández J. J. (2020). Laurinterol from Laurencia johnstonii eliminates Naegleria
fowleri triggering PCD by inhibition of ATPases. Sci. Rep..

[27] Li X.-L., He W.-F., Li J., Lan L.-F., Li X.-W., Guo Y.-W. (2015). New laurane-type
sesquiterpenoids from the Chinese
red alga Laurencia okamurai Yamada. Journal
of Asian natural products research.

[28] Li X.-L., Kurtan T., Hu J.-C., Mandi A., Li J., Li X.-W., Guo Y.-W. (2017). Structural
and Stereochemical Studies
of Laurokamurols A–C, uncommon bis-sesquiterpenoids from the
Chinese red alga Laurencia okamurai Yamada. J. Agric. Food Chem..

[29] Rengasamy K. R., Slavětínská L. P., Kulkarni M. G., Stirk W. A., Van Staden J. (2017). Cuparane sesquiterpenes
from Laurencia natalensis Kylin
as inhibitors of alpha-glucosidase, dipeptidyl peptidase IV and xanthine
oxidase. Algal research.

[30] Topcu G., Aydogmus Z., Imre S., Gören A. C., Pezzuto J. M., Clement J. A., Kingston D. G. (2003). Brominated
Sesquiterpenes
from the Red Alga Laurencia o btusa. J. Nat.
Prod..

[31] Davyt D., Fernandez R., Suescun L., Mombrú A. W., Saldana J., Domínguez L., Coll J., Fujii M. T., Manta E. (2001). New Sesquiterpene Derivatives from the Red Alga Laurencia s coparia.
Isolation, Structure Determination, and Anthelmintic Activity. J. Nat. Prod..

[32] Paul, V. ; Cruz-Rivera, E. ; Thacker, R. , Chemical mediation of macroalgal-herbivore interactions. Marine Chemical Ecology; CRC Press: Boca Raton, Florida, EEUU, 2001, 227–265

[33] Pereira, R. C. ; Da Gama, B. , Macroalgal chemical defenses and their roles in structuring tropical marine communities. Algal chemical ecology; Springer: Berlin, Heidelberg, 2008, 25–55.

[34] Pereira R., Da Gama B., Teixeira V., Yoneshigue-Valentin Y. (2003). Ecological
roles of natural products of the Brazilian red seaweed Laurencia obtusa. Brazilian Journal of Biology.

[35] Nocchi N., Soares A., Souto M., Fernández J., Martin M., Pereira R. (2017). Detection of a chemical
cue from
the host seaweed Laurencia dendroidea by the associated mollusc Aplysia
brasiliana. PLoS One.

[36] Iliopoulou D., Roussis V., Pannecouque C., De Clercq E., Vagias C. (2002). Halogenated sesquiterpenes from the
red alga Laurencia
obtusa. Tetrahedron.

[37] Howard B. M., Fenical W. (1979). Guadalupol and epiguadalupol,
rearranged sesquiterpene
alcohols from Laurencia snyderiae var. guadalupensis. Phytochemistry.

[38] Wright A. D., Goclik E., Konig G. M. (2003). Three new
sesquiterpenes from the
red alga Laurencia perforata. J. Nat. Prod..

[39] González A. G., Aguiar J. M., Martín J. D., Norte M. (1975). Three new sesquiterpenoids
from the marine alga Laurencia perforata. Tetrahedron
Lett..

[40] Gonzalez A. G., Aguiar J. M., Darias J., Gonzalez E., Martin J. D., Martin V. S., Perez C., Fayos J., Martinezripoll M. (1978). Marine Natural-Products
From Atlantic Zone.20. Perforenol, A New Polyhalogenated Sesquiterpene
from Laurencia-Perforata. Tetrahedron Lett..

[41] Findlay J. A., Li G. Q. (2002). Novel terpenoids
from the Sea Hare Aplysia punctata. Canadian
Journal of Chemistry-Revue Canadienne De Chimie.

[42] Tokalı F. S., Şenol H., Ateşoğlu Ş., Akbaş F. (2024). A series of
quinazolin-4­(3H)-one-morpholine hybrids as anti-lung-cancer agents:
Synthesis, molecular docking, molecular dynamics, ADME prediction
and biological activity studies. Chem. Biol.
Drug Des..

[43] Tahirli S., Aliyeva F., Şenol H., Demukhamedova S., Akverdieva G., Aliyeva I., Veysova S., Sadeghian N., Gunay S., Erden Y., Taslimi P., Sujayev A., Chiragov F. (2024). Novel complex compounds of nickel
with 3-(1-phenyl-2,3-dimethyl-pyrazolone-5)­azopentadione-2,4:
synthesis, NBO analysis, reactivity descriptors and in silico and *in vitro* anti-cancer and bioactivity studies. J. Biomol. Struct. Dyn..

[44] Halil Ş., Berre M., Rabia Büşra Ş., Halil
Burak K., Ebru H. (2022). Synthesis of oleanolic acid hydrazide-hydrazone
hybrid derivatives and investigation of their cytotoxic effects on
A549 human lung cancer cells. Res. Chem..

[45] Toraman G., Senol H., Tütünis S. Y., Tan N. R., Topçu G. (2023). Phytochemical analysis and molecular
docking studies
of two endemic varieties of Salvia sericeotomentosa. Turk. J. Chem..

[46] Şenol H., Çokuludağ K., Aktaş A. S., Atasoy S., Dağ A., Topçu G. (2020). Synthesis
of new fatty acid derivatives of oleanane and ursane triterpenoids
and investigation of their*in vitro* cytotoxic effects
on 3T3 fibroblast and PC3 prostate cancer cell lines. Org. Commun..

[47] Ateşoğlu Ş., Çakır F., Şenol A. M., Tokalı P., Şenol H., Tokalı F. S., Akbaş F., Kalay E. (2025). Synthesis, Characterization
and *In Vitro* & *In Silico* Biological
Evaluation
of New Mannich-Based Rhodanine and Thiazolidine-2,4-Dione Derivatives
as Potential Anti-Lung Cancer Agents. Synlett.

[48] Kılınç N. (2021). Inhibition
profiles and molecular docking studies of antiproliferative agents
against aldose reductase enzyme. International
Journal of Chemistry and Technology.

[49] Köksal Z., Şenol H. (2025). Anticholinesterase
and carbonic anhydrase inhibitory
activities of natural carnosic acid derivatives: A comprehensive *in vitro* and *in silico* study. Arch. Pharm..

[50] Zengin
Kurt B., Öztürk Civelek D., Çakmak E. B., Kolcuoğlu Y., Şenol H., Sağlık Özkan B. N., Dag A., Benkli K. (2024). Synthesis of Sorafenib–Ruthenium Complexes,
Investigation of Biological Activities and Applications in Drug Delivery
Systems as an Anticancer Agent. J. Med. Chem..

