# Supplementary material for: Four New Perforane-Type Sesquiterpenes from Laurencia obtusa (Hudson) J.V. Lamouroux as Potent Lung Cancer Inhibitors: Isolation, Structure Elucidation, Cytotoxicity, Molecular Docking, Dynamics, and ADME Studies
Source: ACS Omega. 2026 Jan 12;11(3):4135–54. doi: 10.1021/acsomega.5c08806 (PMC12854617; doi:10.1021/acsomega.5c08806)
Supplement: Supplementary file 1 [file ao5c08806_si_001.pdf]

Four New Perforane-Type Sesquiterpenes from *Laurencia obtusa* (Hudson) J.V. Lamouroux as Potent Lung Cancer Inhibitors: Isolation, Structure Elucidation, Cytotoxicity, Molecular Docking, Dynamics and ADME Studies

Özlem Demirkıran[1,*], Halil Şenol[2], Yağmur Elci[1], Elif Coşkun[1], Gülbahar Özge Alim Toraman[3], Ebru Erol[4], Emine Şükran Okudan[5], Gülaçtı Topçu[3,6,*]

[1]Department of Pharmacognosy, Faculty of Pharmacy, Trakya University, 22030, Edirne, Türkiye

[2]Department of Pharmaceutical Chemistry, Faculty of Pharmacy, Bezmialem Vakif University, 34093, Fatih, Istanbul, Türkiye

[3]Department of Pharmacognosy, Faculty of Pharmacy, Bezmialem Vakif University, 34093, Fatih, Istanbul, Türkiye

[4]Department of Analytical Chemistry, Faculty of Pharmacy, Bezmialem Vakif University, 34093, Fatih, Istanbul, Türkiye

[5]Faculty of Aquatic Sciences and Fisheries, Akdeniz University, 07058, Antalya, Türkiye

[6] Drug Application and Research Center, Bezmialem Vakif University, 34093, Fatih, Istanbul, Türkiye

*Corresponding authors: Gülaçtı Topçu (gtopcu@bezmialem.edu.tr) and Ozlem Demirkiran (ozlemdemirkiran@trakya.edu.tr)

Content

	Supporting data	Page
**Fig. S1.**	^{1}H-NMR of Compound **1** (500 MHz, $CDCl_3$)	S5
**Fig. S2.**	APT of Compound **1** (125 MHz, $CDCl_3$)	S6
**Fig. S3.**	COSY of Compound **1**	S7
**Fig. S4.**	HSQC of Compound **1**	S8
**Fig. S5.**	HMBC of Compound **1**	S9
**Fig. S6.**	NOESY of Compound **1**	S10
**Fig. S7.**	HRESIMS of Compound **1**	S11
**Fig. S8.**	Expanded HRESIMS of Compound **1**, 310–342 *m/z*	S12
**Fig. S9.**	IR spectrum of Compound **1**	S13
**Fig. S10.**	UV spectrum of Compound **1**	S14
**Fig. S11.**	^{1}H-NMR of Compound **2** (500 MHz, $CDCl_3$)	S15
**Fig. S12.**	APT of Compound **2** (125 MHz, $CDCl_3$)	S16
**Fig. S13.**	COSY of Compound **2**	S17
**Fig. S14.**	HSQC of Compound **2**	S18
**Fig. S15.**	HMBC of Compound **2**	S19
**Fig. S16.**	NOESY of Compound **2**	S20
**Fig. S17.**	HRESIMS of Compound **2**	S21
**Fig. S18.**	HRESIMS of Compound **2**, 312–324 m/z	S22
**Fig. S19.**	IR spectrum of Compound **2**	S23
**Fig. S20.**	UV spectrum of Compound **2**	S24

**Fig. S21.**	^{1}H-NMR of Compound **3** (500 MHz, $CDCl_3$)	S25
**Fig. S22.**	APT of Compound **3** (125 MHz, $CDCl_3$)	S26
**Fig. S23.**	COSY of Compound **3**	S27
**Fig. S24.**	HSQC of Compound **3**	S28
**Fig. S25.**	HMBC of Compound **3**	S29
**Fig. S26.**	NOESY of Compound **3**	S30
**Fig. S27.**	HRESIMS of Compound **3**	S31
**Fig. S28.**	IR spectrum of Compound **3**	S32
**Fig. S29.**	UV spectrum of Compound **3**	S33
**Fig. S30.**	^{1}H-NMR of Compound **4** (500 MHz, $CDCl_3$)	S34
**Fig. S31.**	APT of Compound **4** (125 MHz, $CDCl_3$)	S35
**Fig. S32.**	COSY of Compound **4**	S36
**Fig. S33.**	HSQC of Compound **4**	S37
**Fig. S34.**	HMBC of Compound **4**	S38
**Fig. S35.**	NOESY of Compound **4**	S39
**Fig. S36.**	HRESIMS of Compound **4**	S40
**Fig. S37.**	IR spectrum of Compound **4**	S41
**Fig. S38.**	UV spectrum of Compound **4**	S42
**Fig. S39.**	The 100 ns MD simulation analysis of **1-VEGFR1** complex.	S43
**Fig. S40.**	The 100 ns MD simulation analysis of **2-VEGFR1** complex.	S44
**Fig. S41.**	The 100 ns MD simulation analysis of **4-VEGFR1** complex.	S45
**Fig. S42.**	The 100 ns MD simulation analysis of **1-VEGFR2** complex.	S46

**Fig. S43.**	The 100 ns MD simulation analysis of **2-VEGFR2** complex.	S47
**Fig. S44.**	The 100 ns MD simulation analysis of **4-VEGFR2** complex.	S48
**Fig. S45.**	The 100 ns MD simulation analysis of **1-EGFR2** complex.	S49
**Fig. S46.**	The 100 ns MD simulation analysis of **3-EGFR2** complex.	S50
**Fig. S47.**	The 100 ns MD simulation analysis of **4-EGFR2** complex.	S51
**Table S1.**	NMR data of compound **1**	S52
**Table S2.**	NMR data of compound **2**	S53
**Table S3.**	NMR data of compound **3**	S54
**Table S4.**	NMR data of compound **4**	S55
**Table S5.**	Impurity of the compound **1** (LC(11-14)-19A) by qHNMR	S56
**Table S6.**	Impurity of the compound **2** (LC(11-14)-19A) by qHNMR	S58
**Table S7.**	Impurity of the compound **3** (LC(11-14)-19A) by qHNMR	S60
**Table S8.**	Impurity of the compound **4** (LC(11-14)-19A) by qHNMR	S62

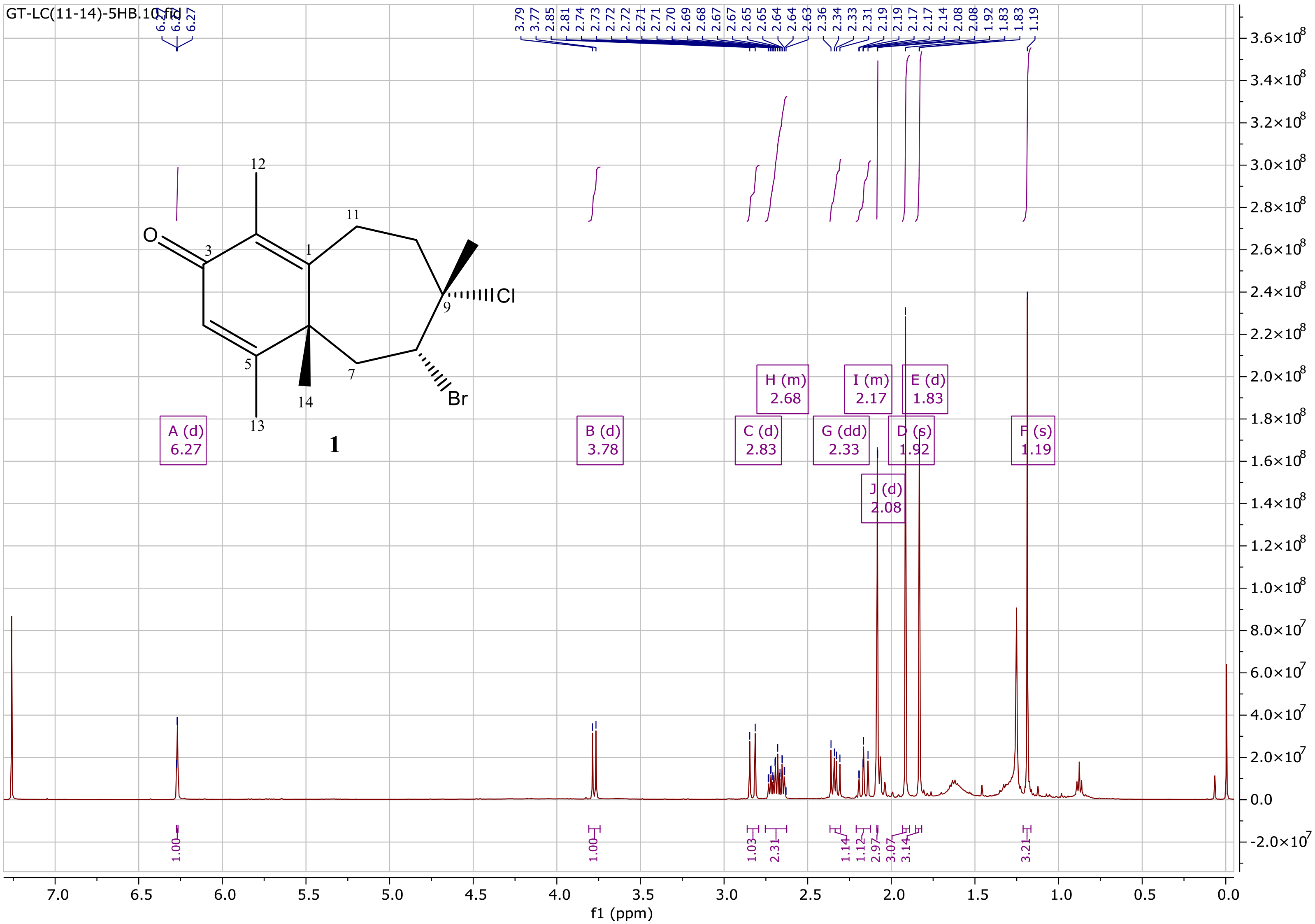

Figure S1. ^{1}H-NMR of Compound **1** (500 MHz, $CDCl_3$)

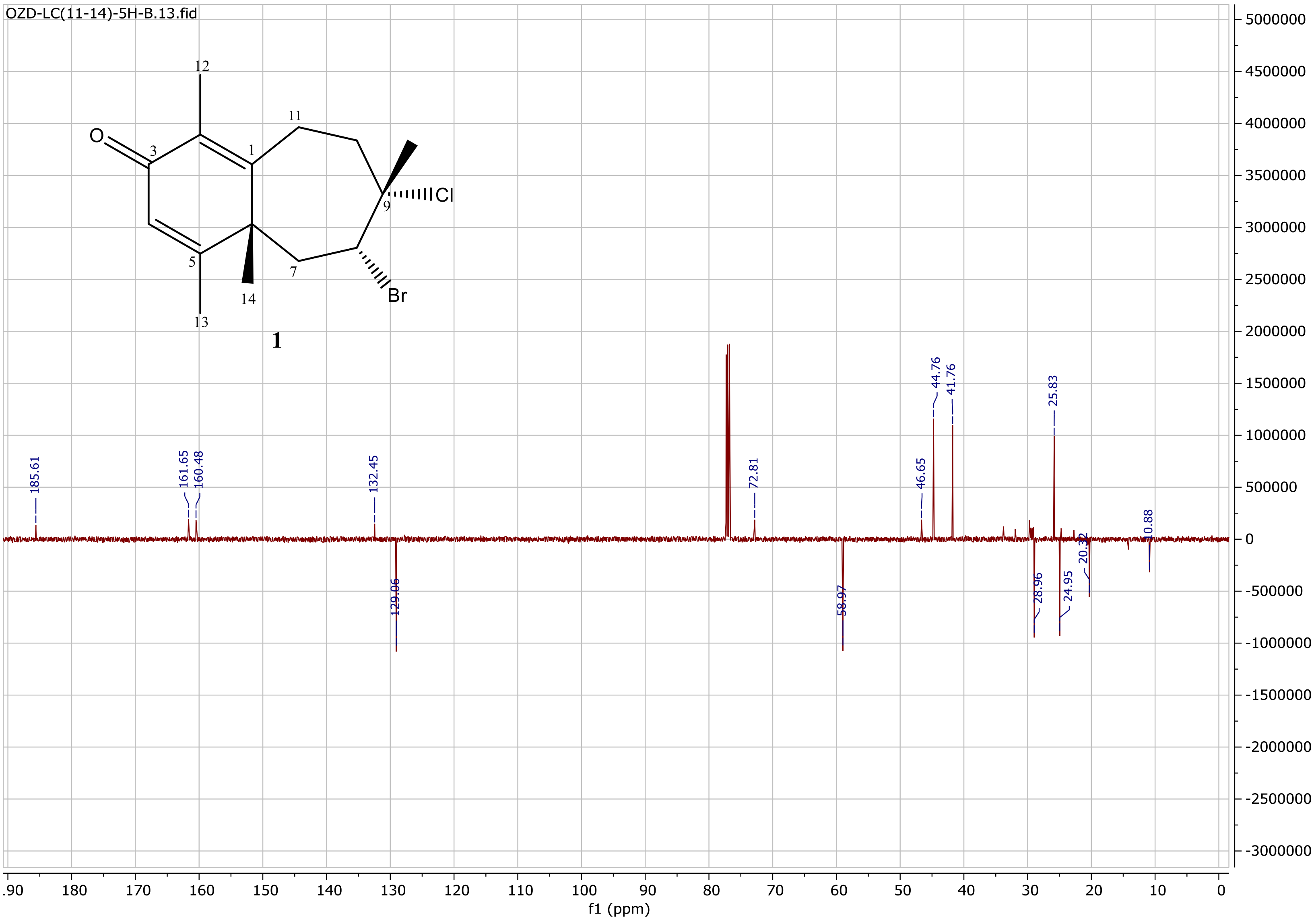

Figure S2. APT of Compound **1** (125 MHz, $CDCl_3$)

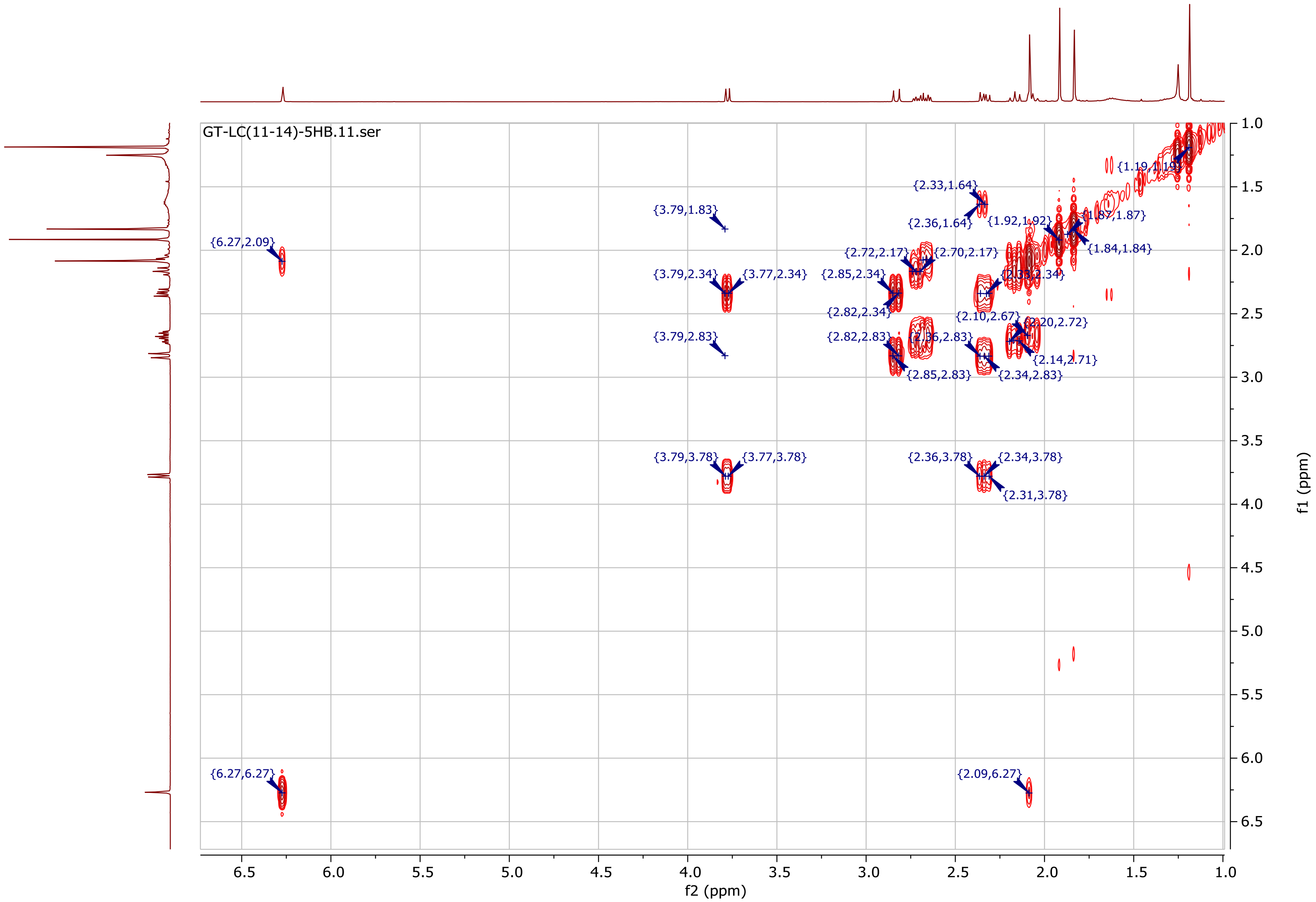

Figure S3. COSY of Compound **1**

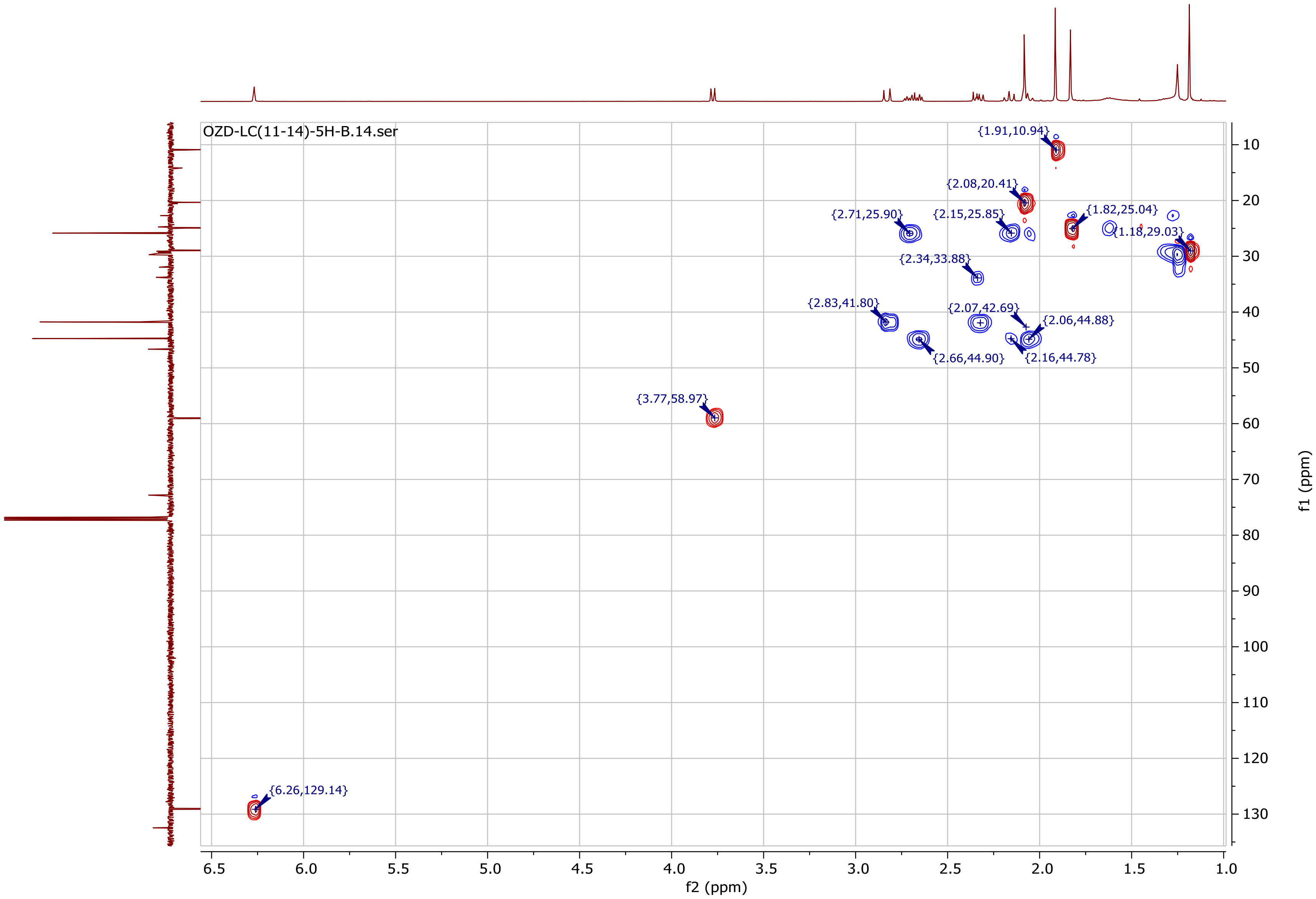

Figure S4. HSQC of Compound **1**

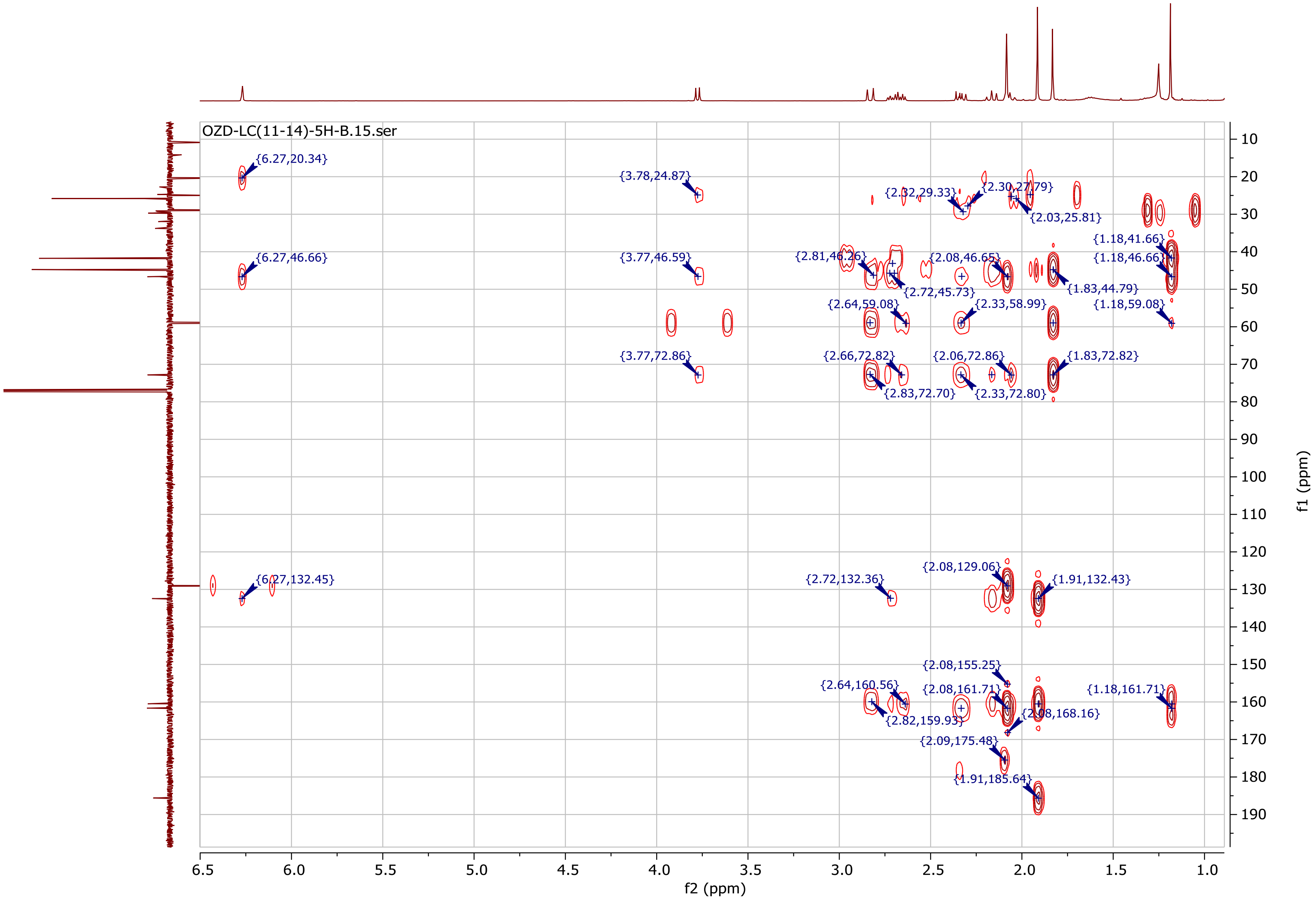

Figure S5. HMBC of Compound **1**

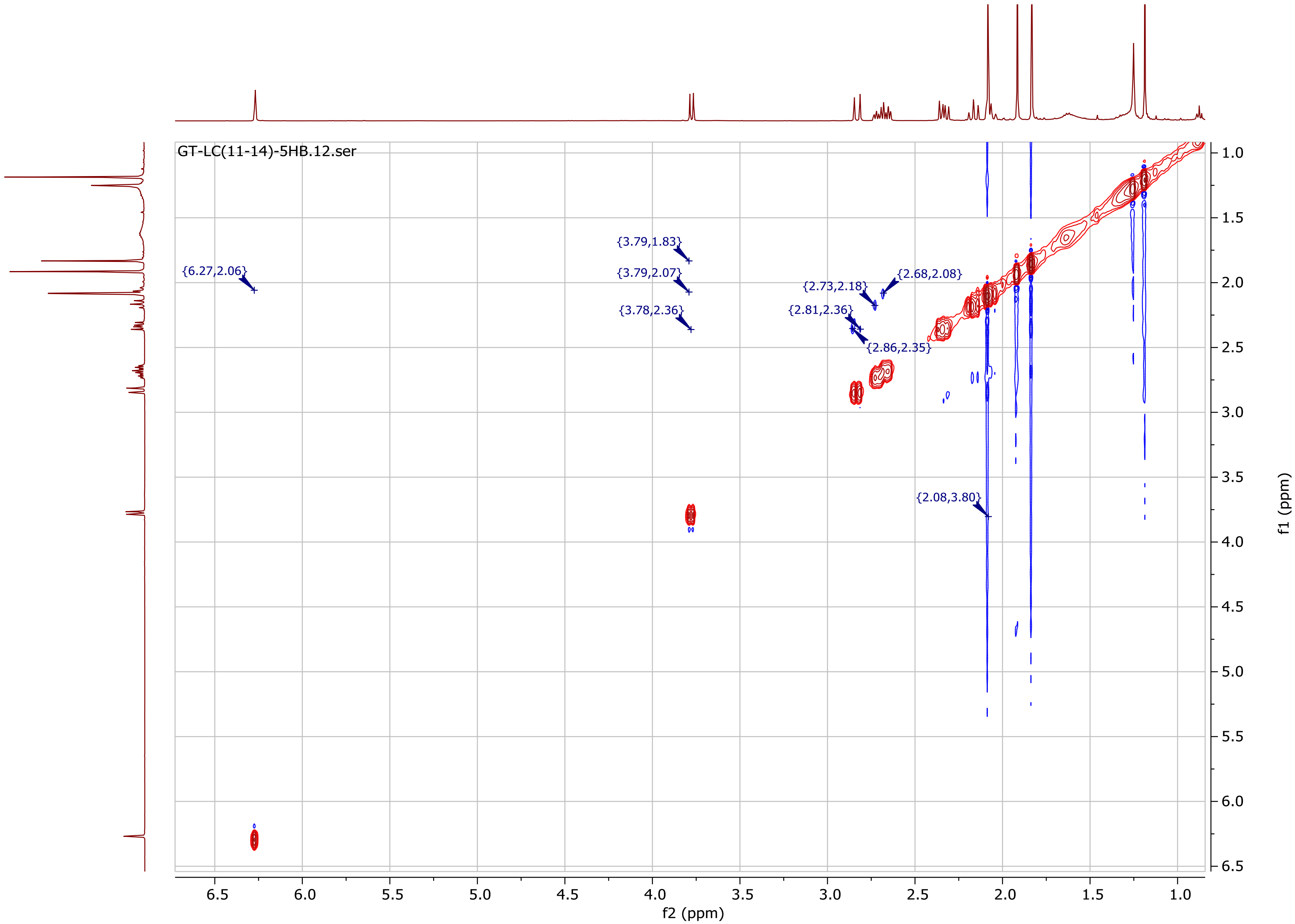

Figure S6. NOESY of Compound **1**

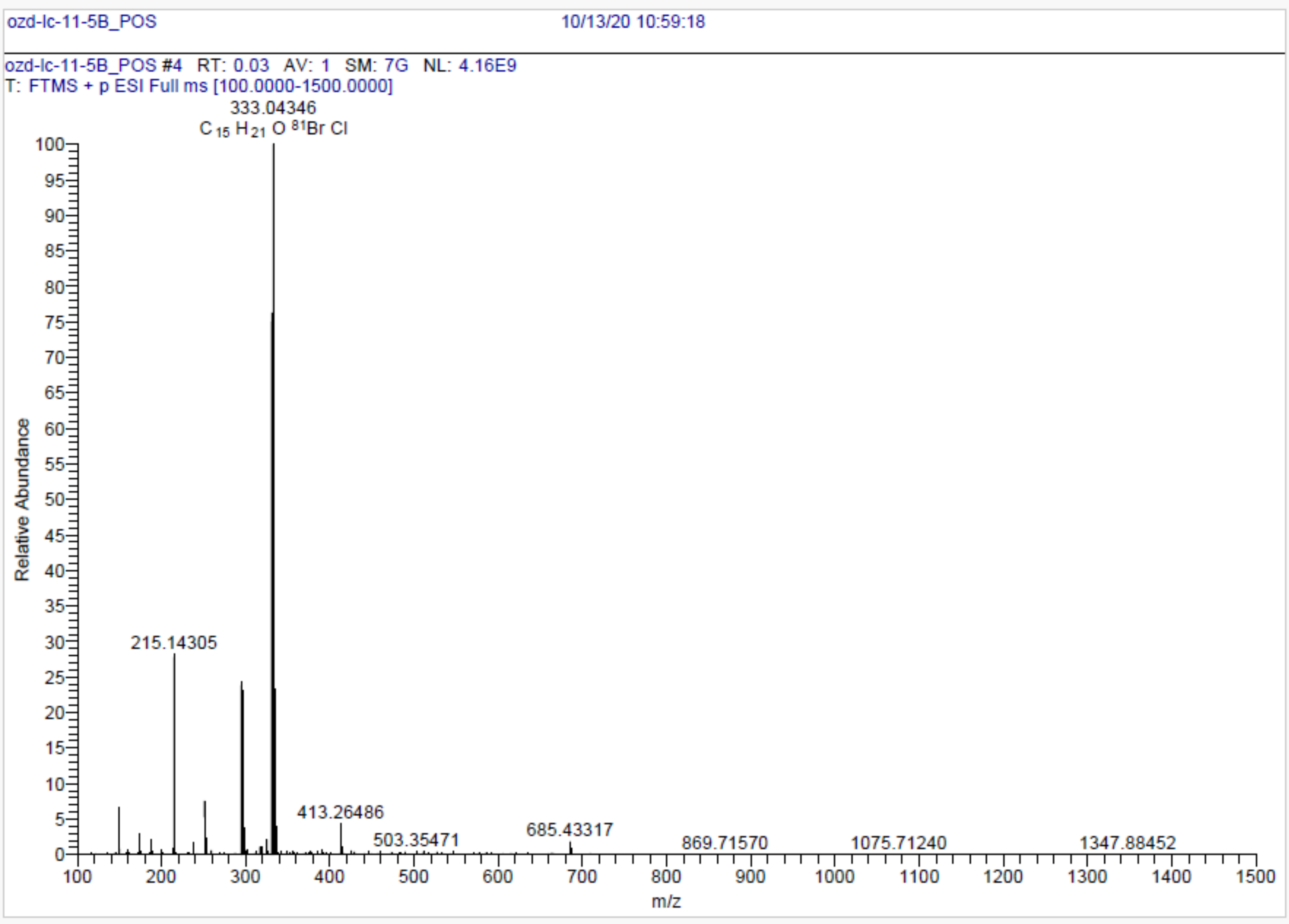

Figure S7. HRESIMS of Compound **1**

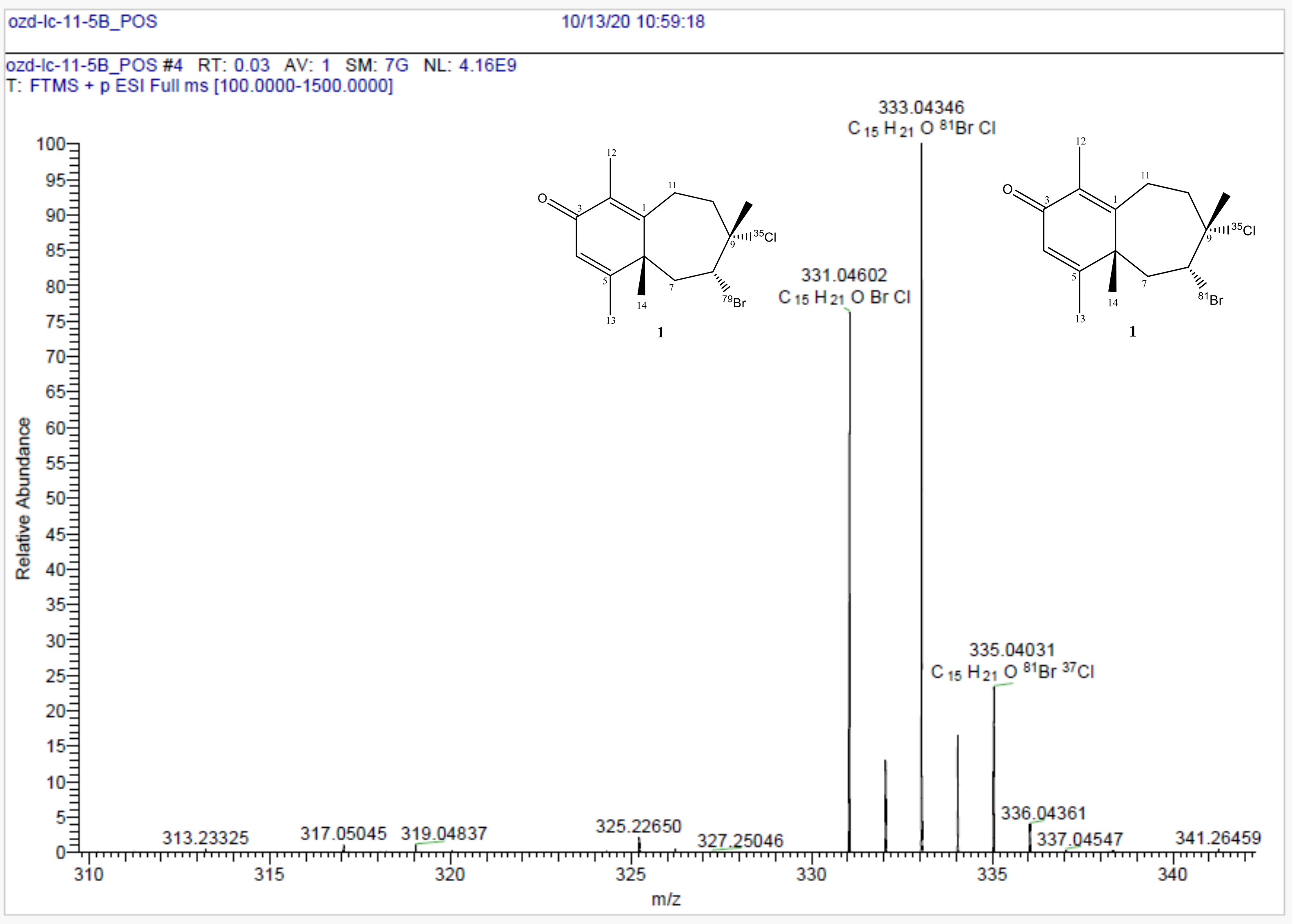

Figure S8. Expanded HRESIMS of Compound **1**, 310–342 *m/z*

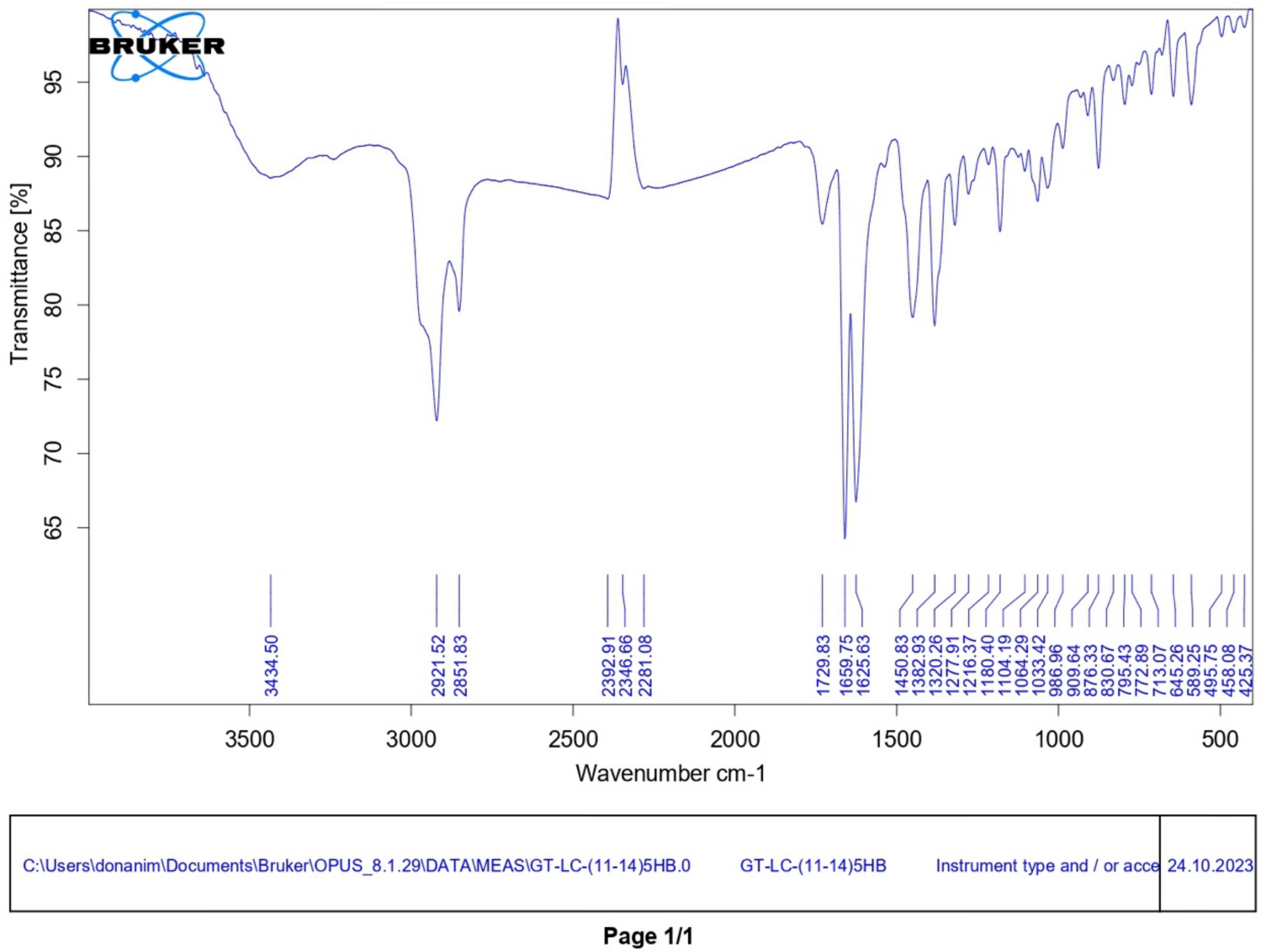

Figure S9. IR spectrum of Compound **1**

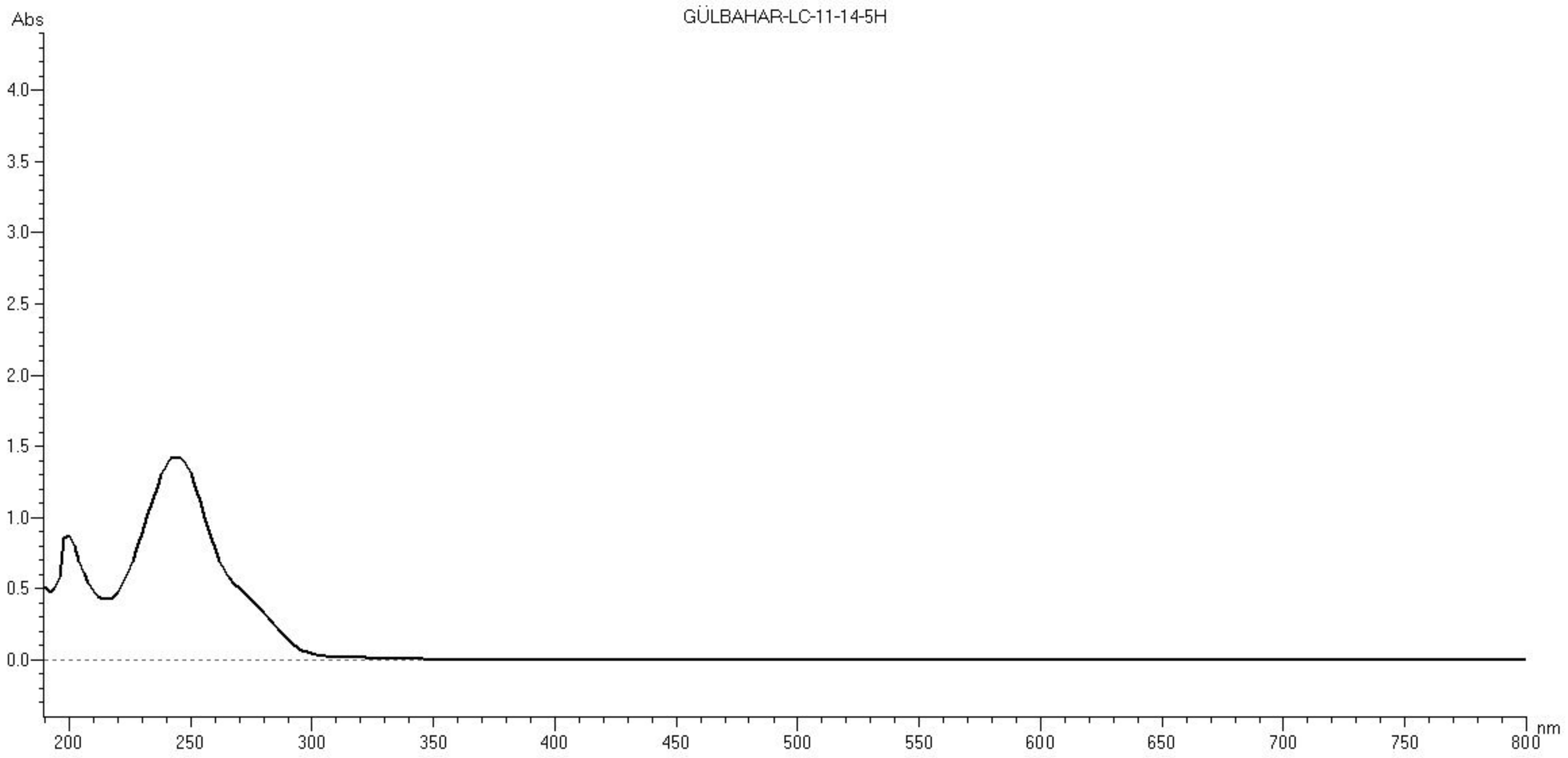

Figure S10. UV spectrum of Compound **1**

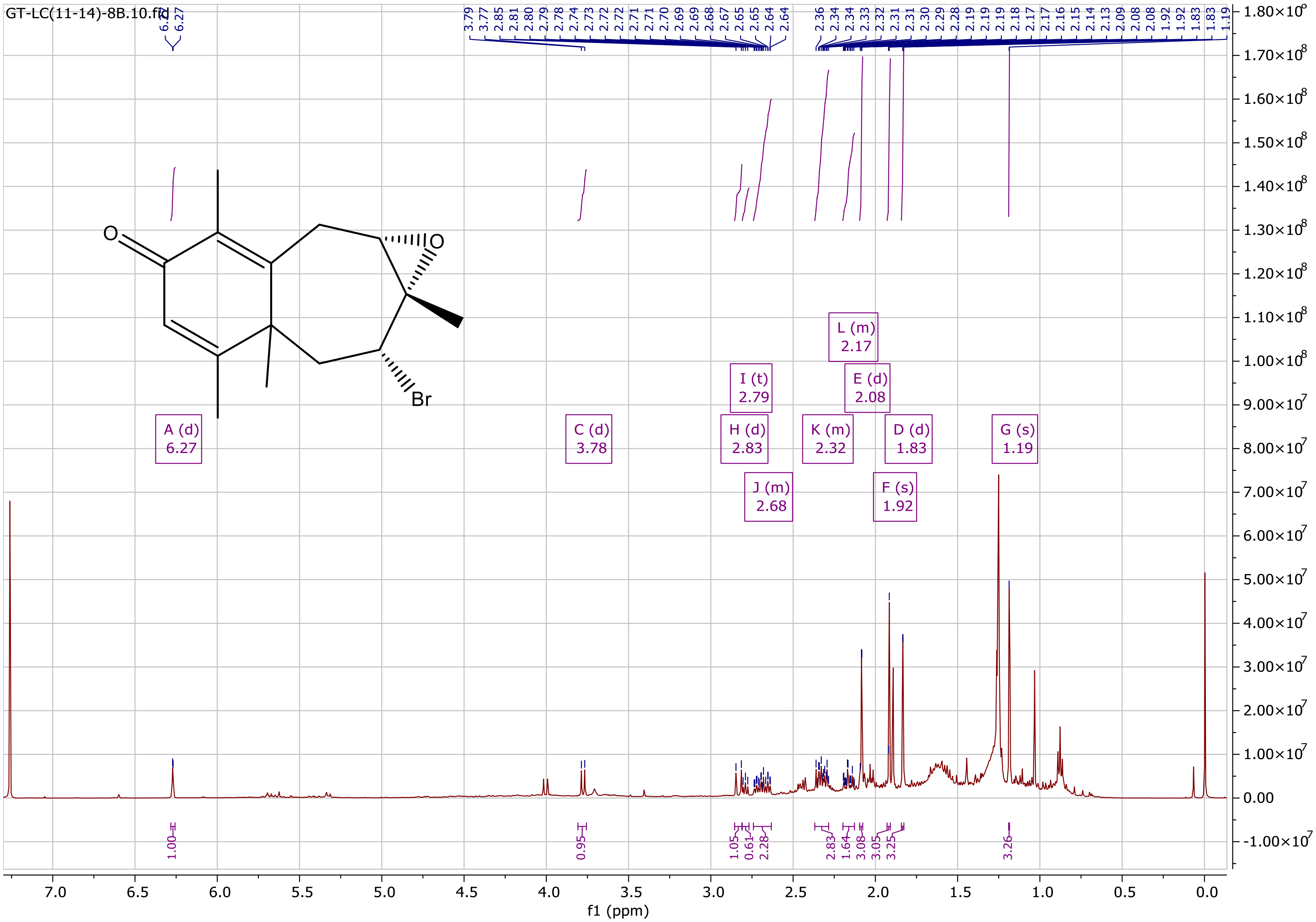

Figure S11. ^{1}H-NMR of Compound **2** (500 Hz, $CDCl_3$)

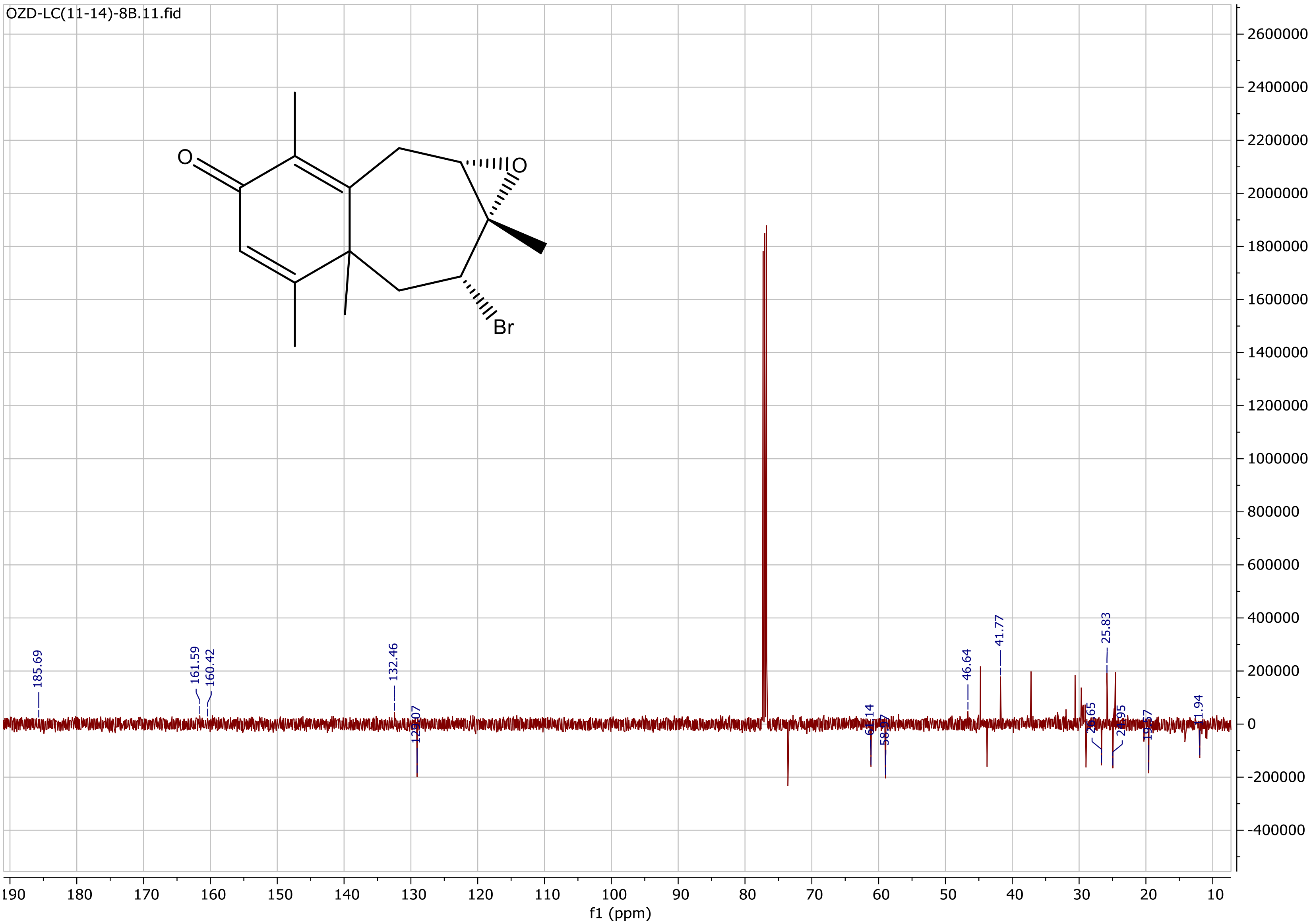

Figure S12. APT of Compound **2** (125 Hz, $CDCl_3$)

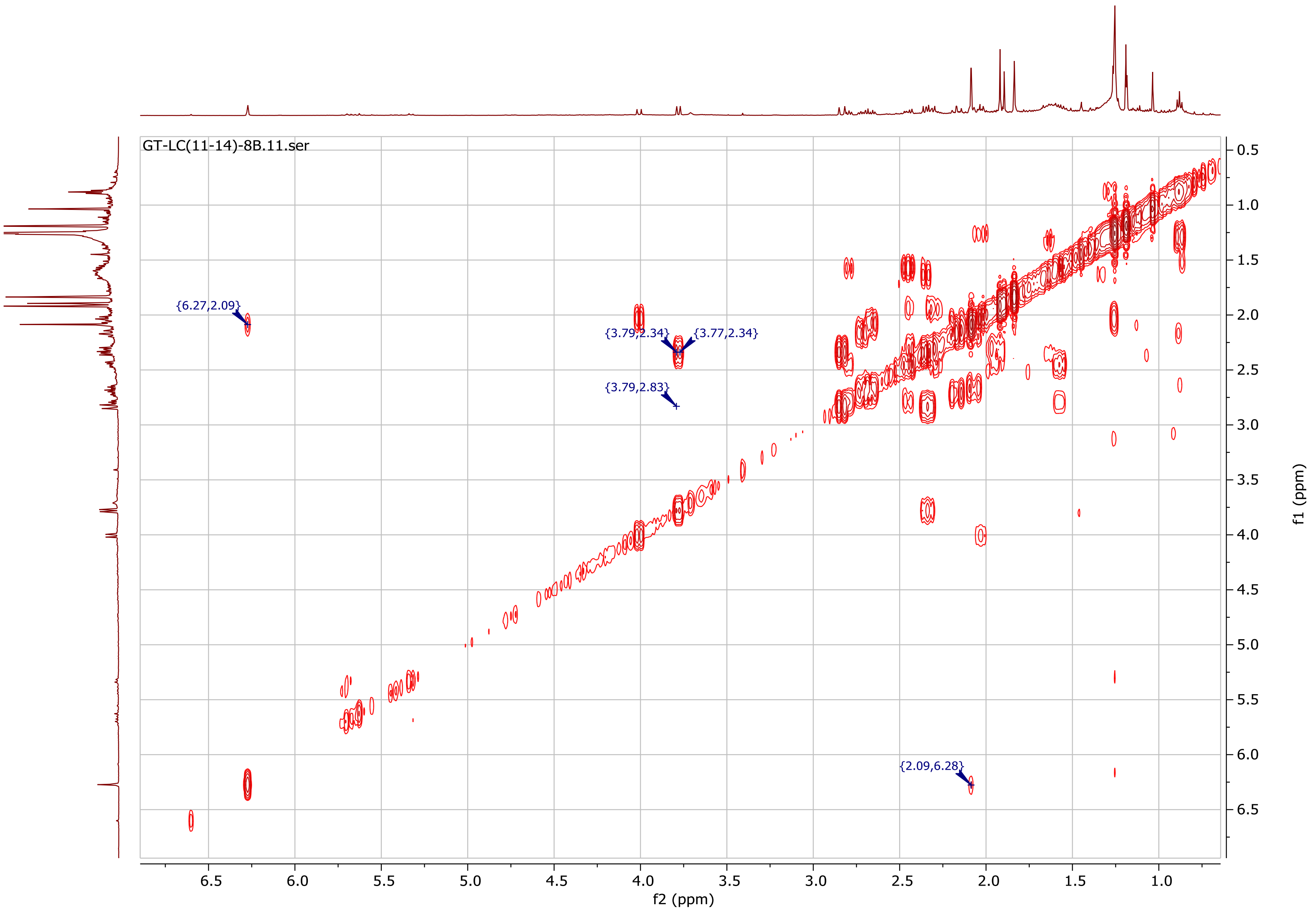

Figure S13. COSY of Compound **2**

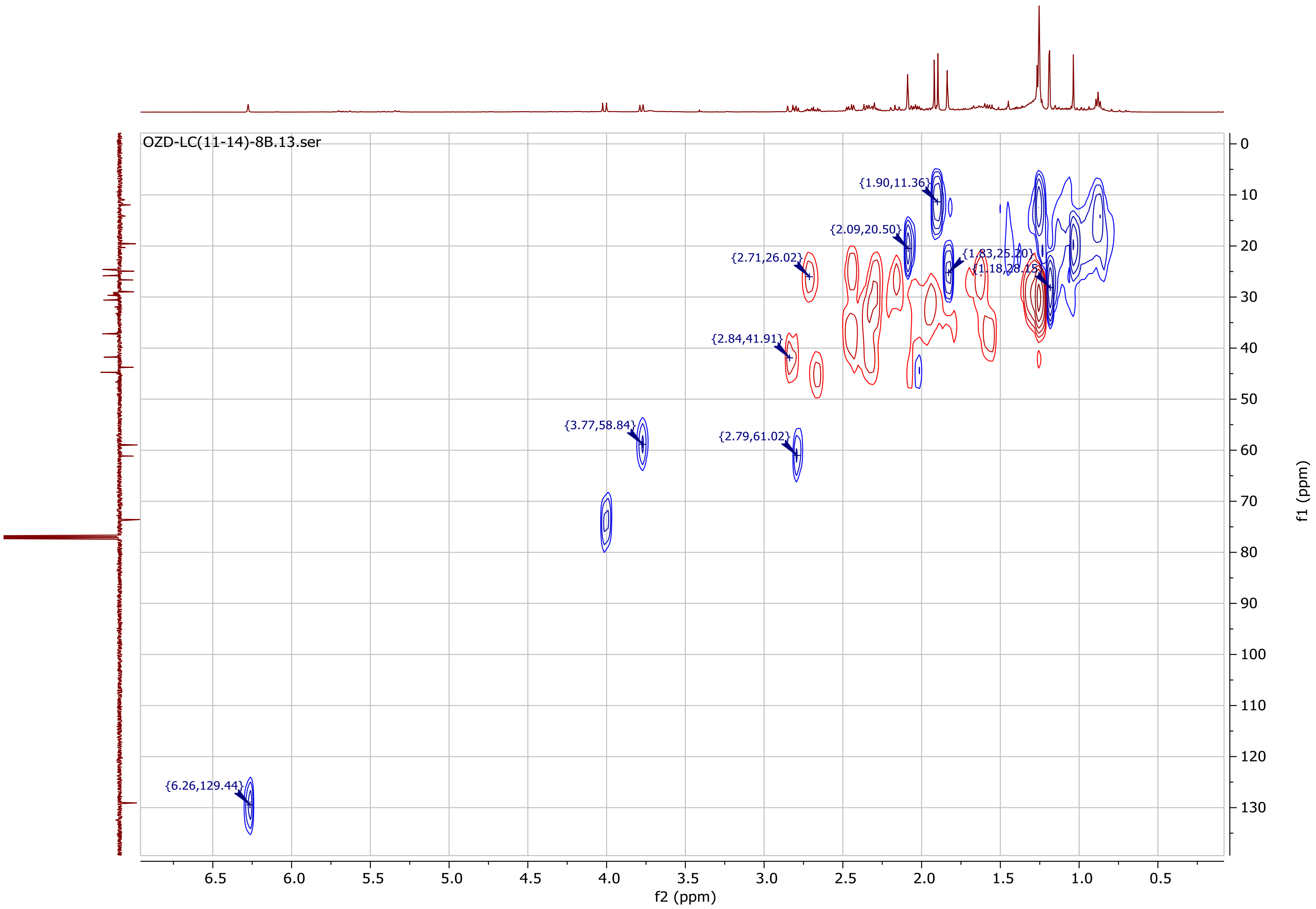

Figure S14. HSQC of Compound **2**

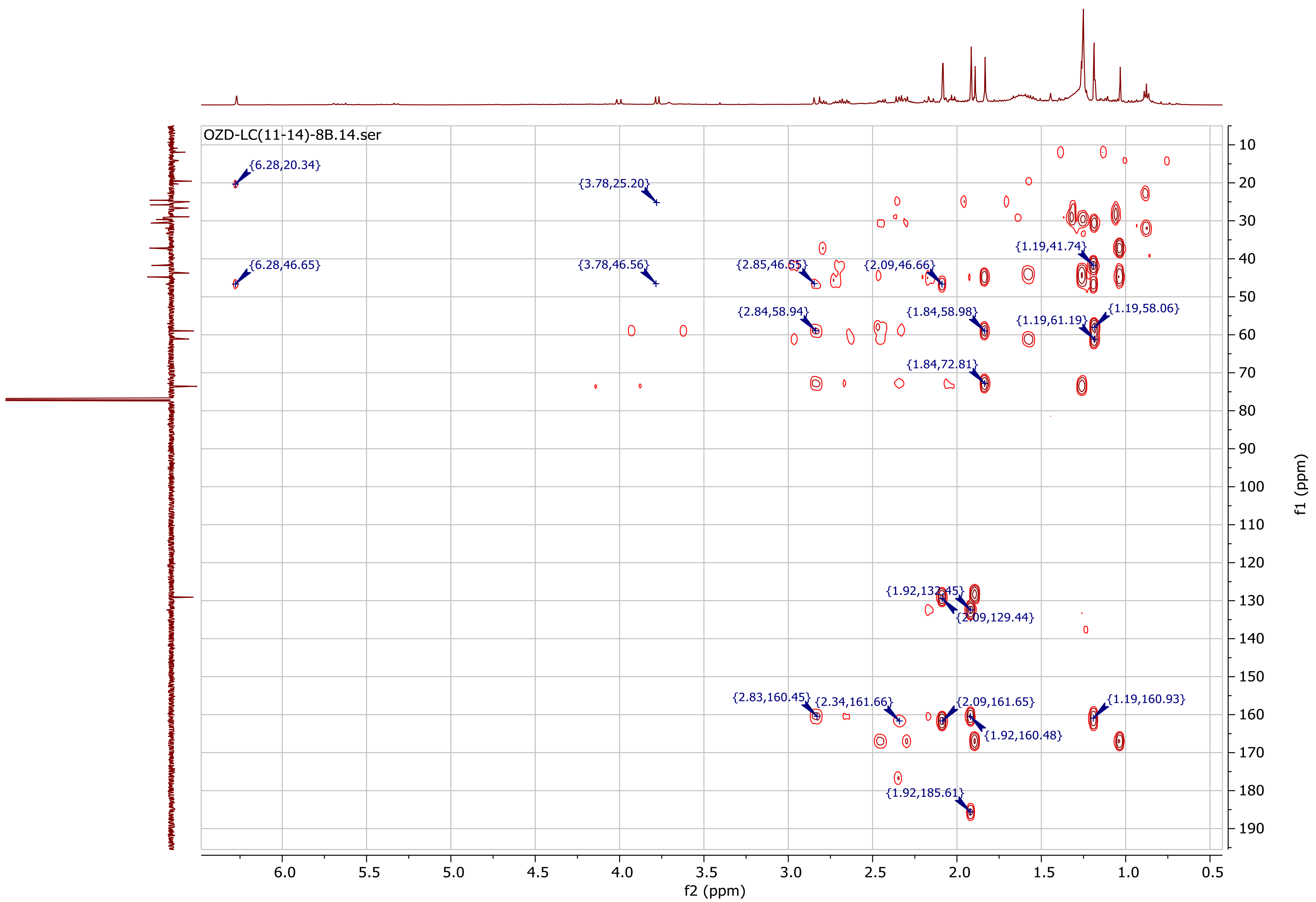

Figure S15. HMBC of Compound **2**

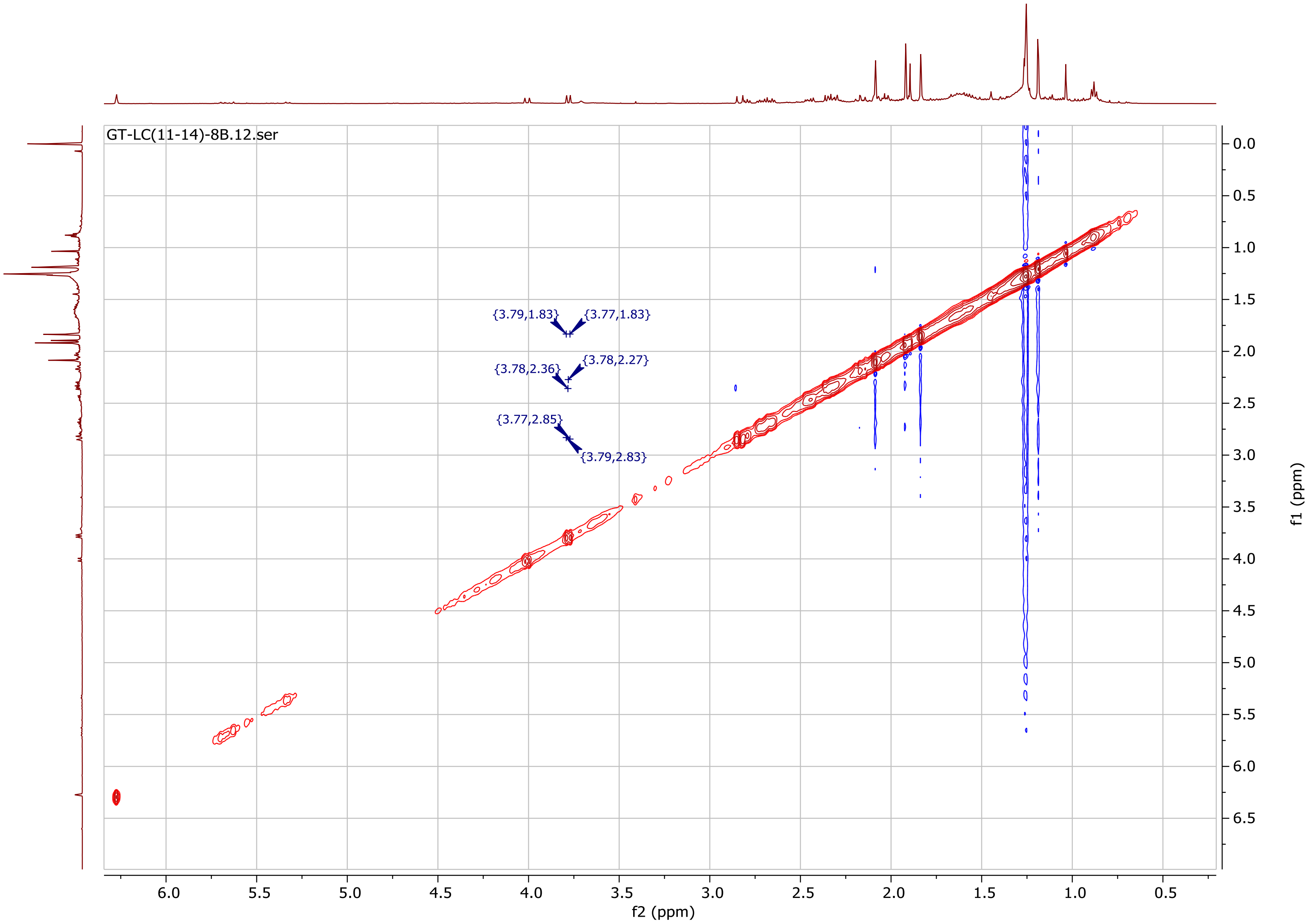

Figure S16. NOESY of Compound **2**

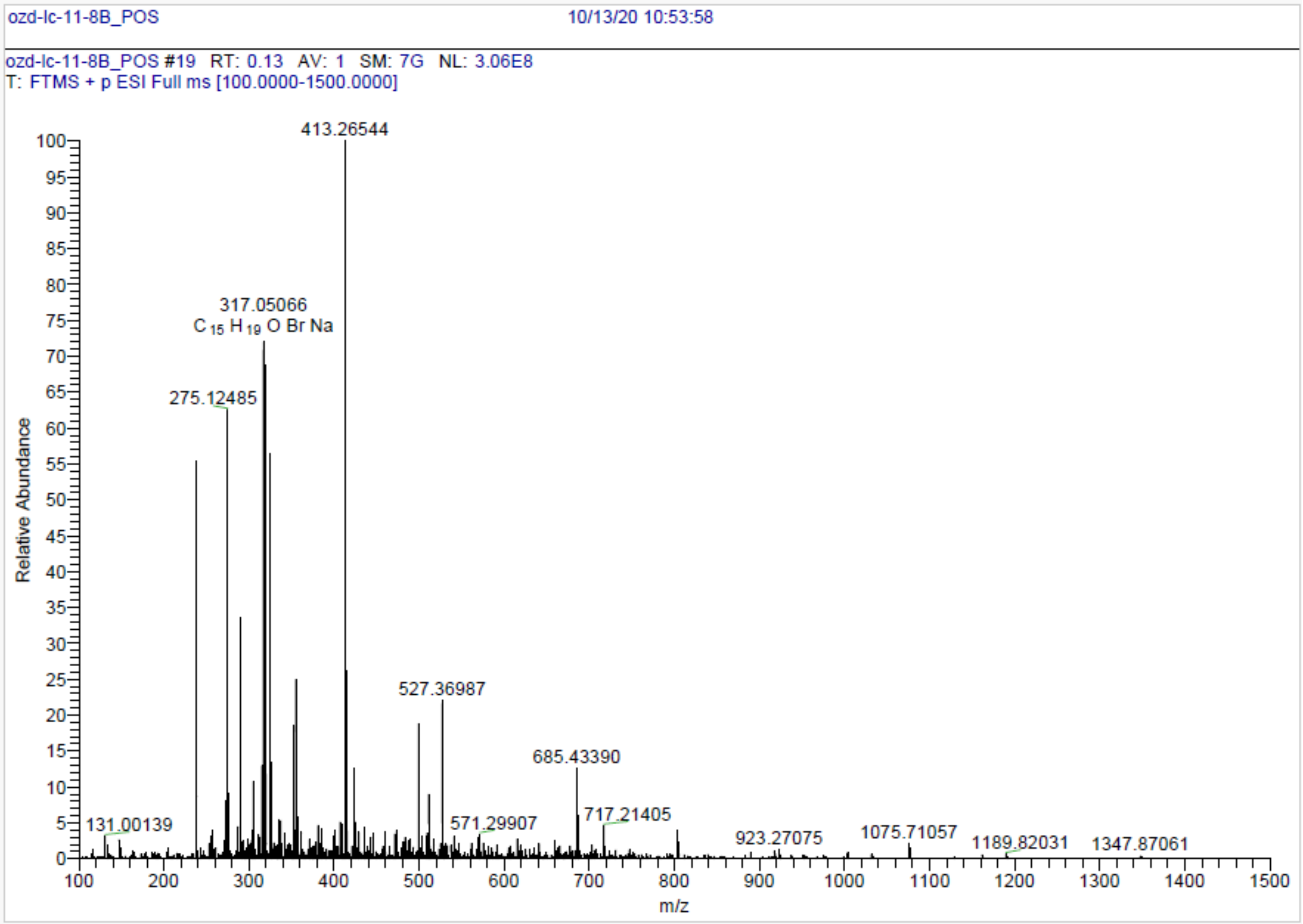

Figure S17. HRESIMS of Compound **2**

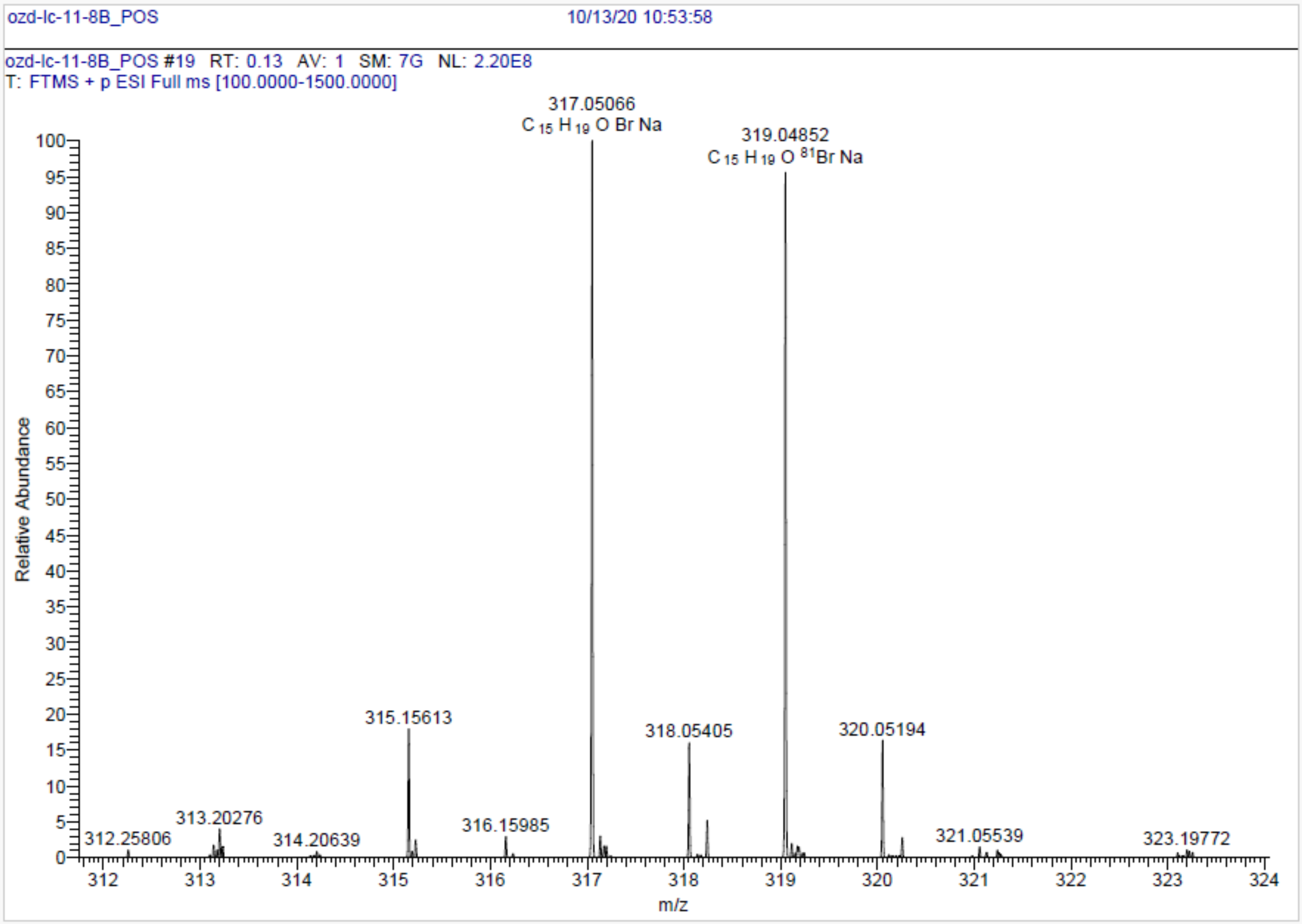

Figure S18. HRESIMS of Compound **2**, 312–324 *m/z*

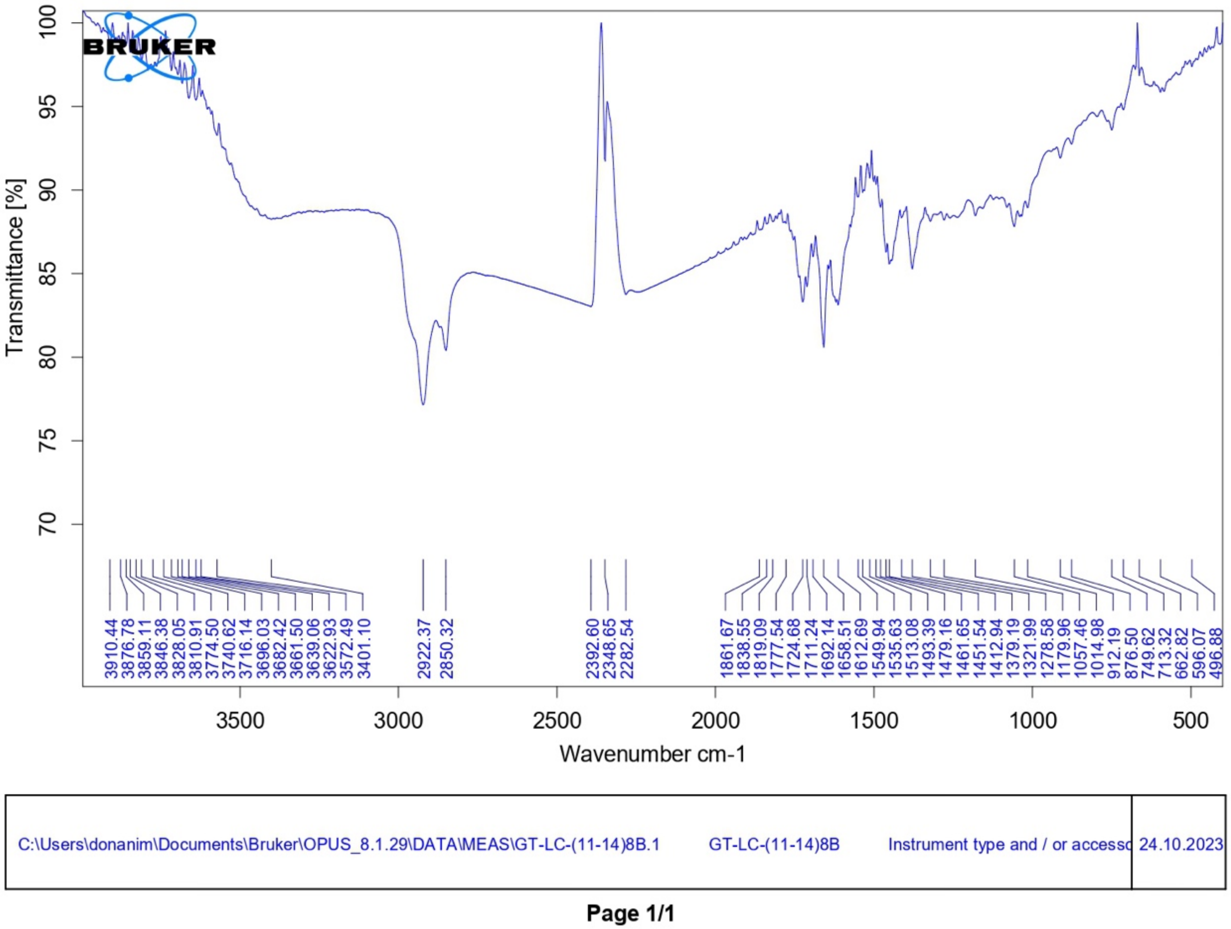

Figure S19. IR spectrum of Compound **2**

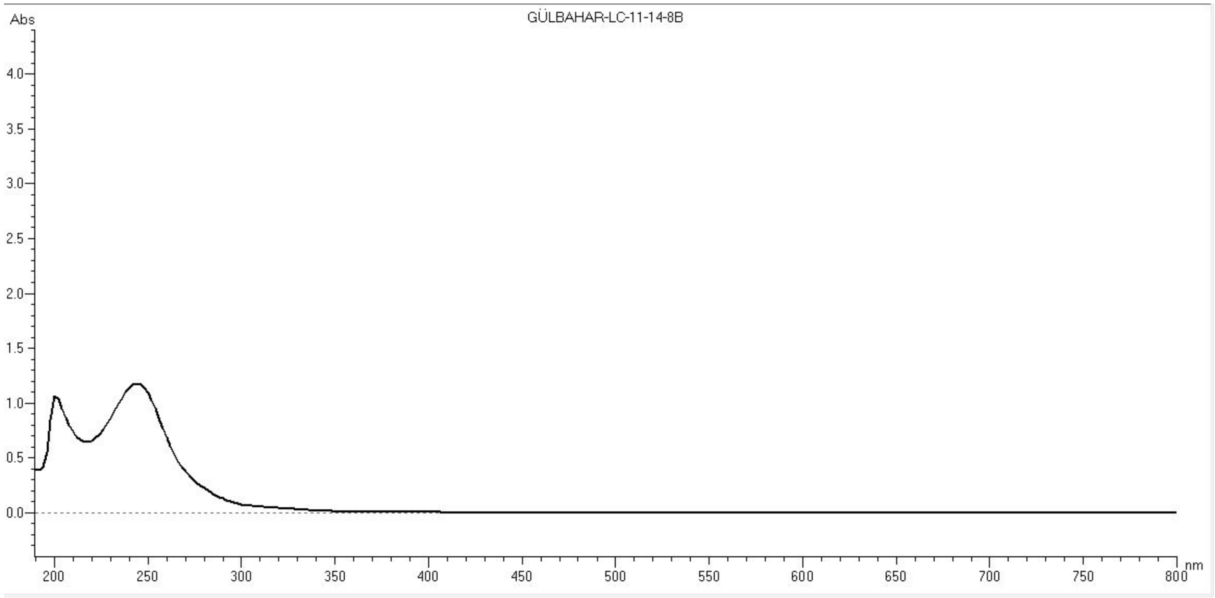

Figure S20. UV spectrum of Compound **2**

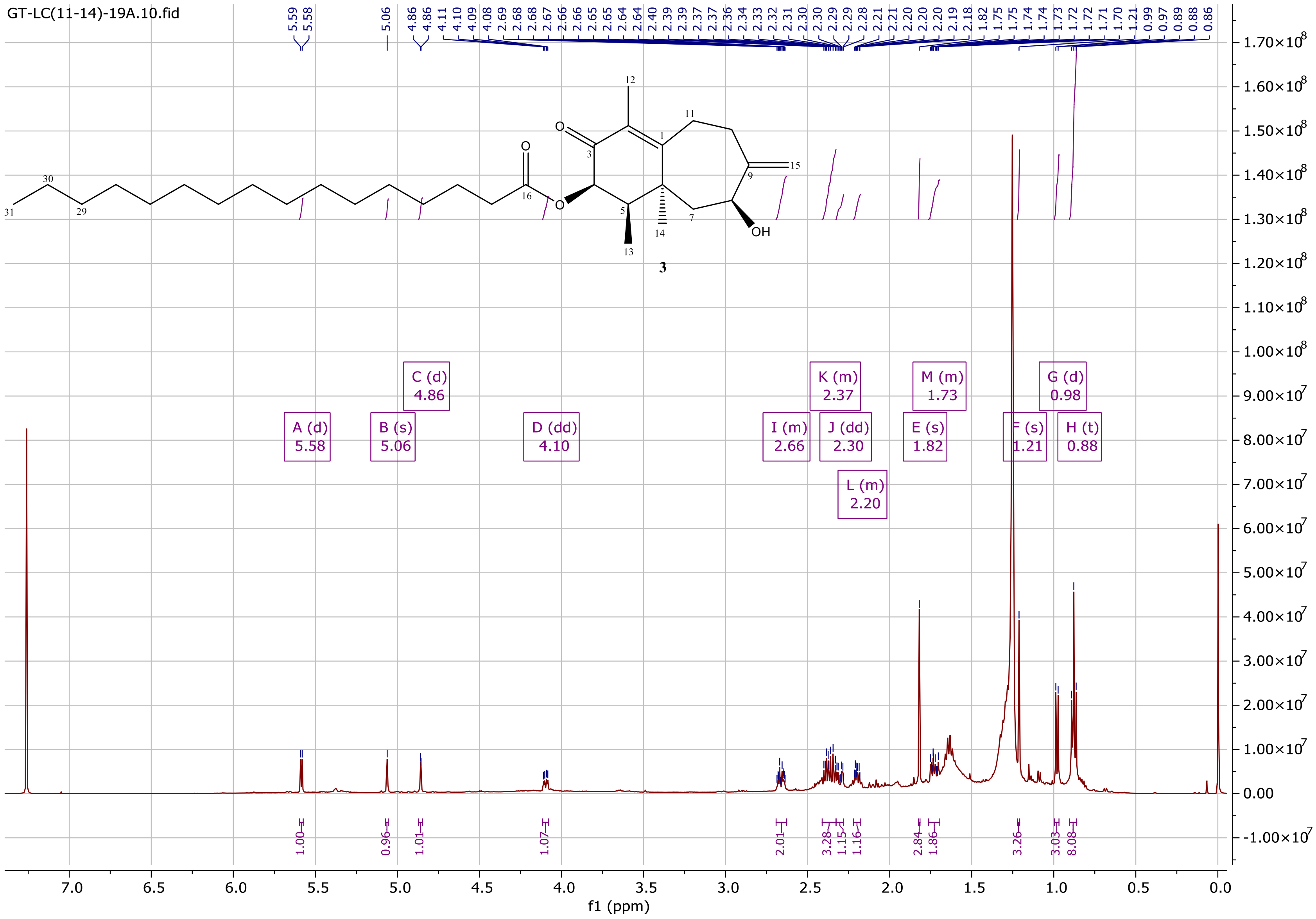

Figure S21. ^{1}H-NMR of Compound **3** (500 Hz, $CDCl_3$)

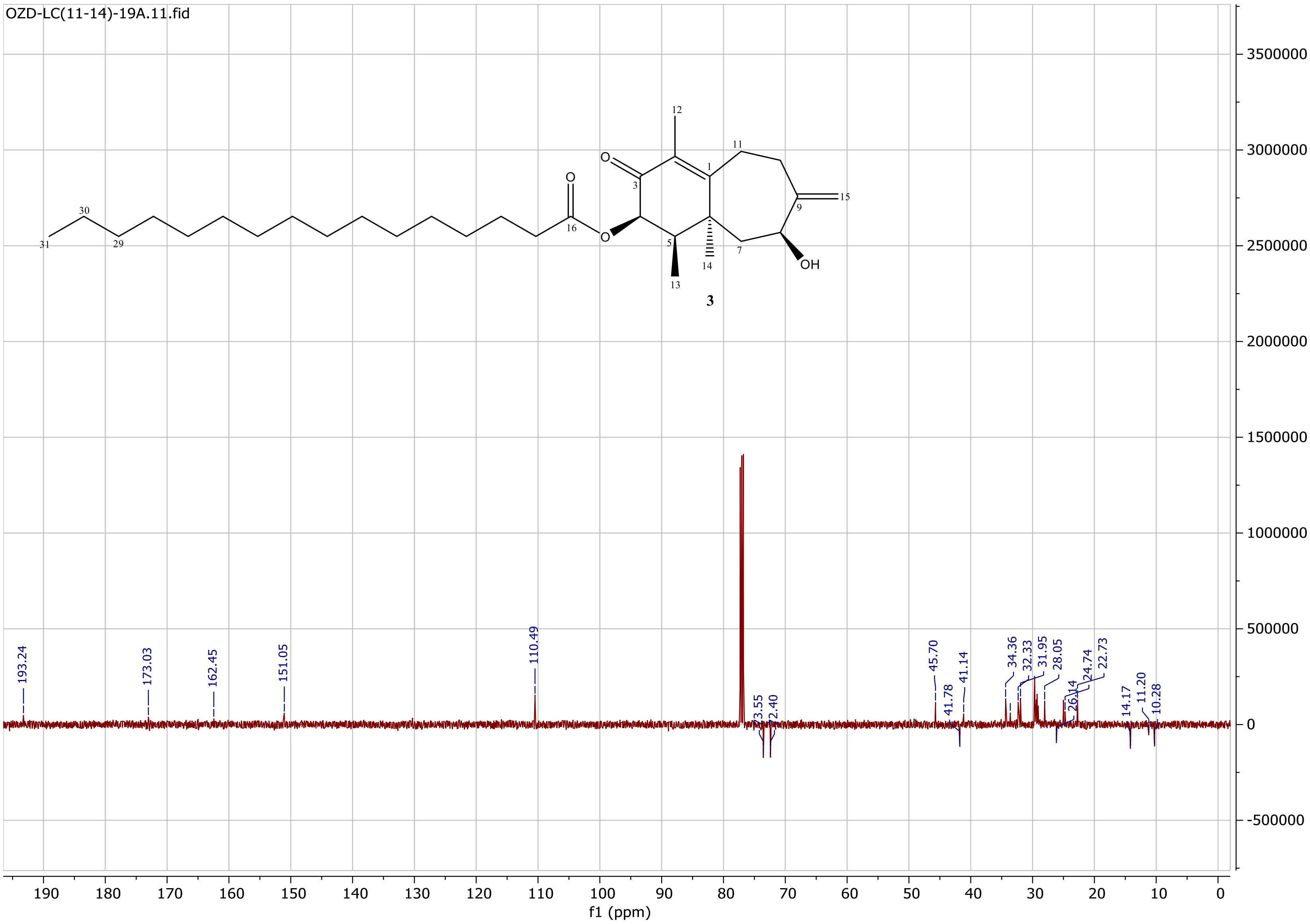

Figure S22. APT of Compound **3** (125 Hz, $CDCl_3$)

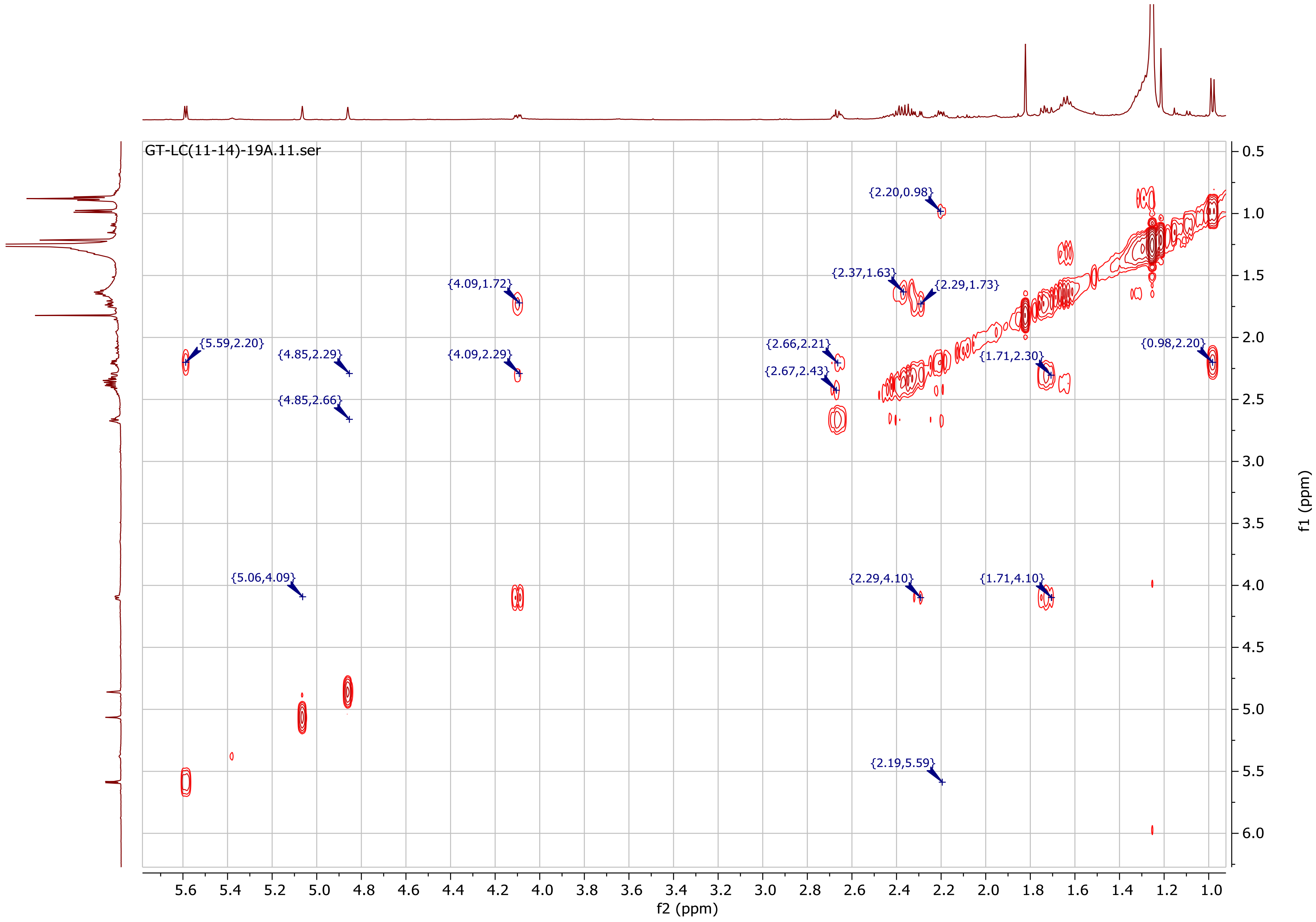

Figure S23. COSY of Compound **3**

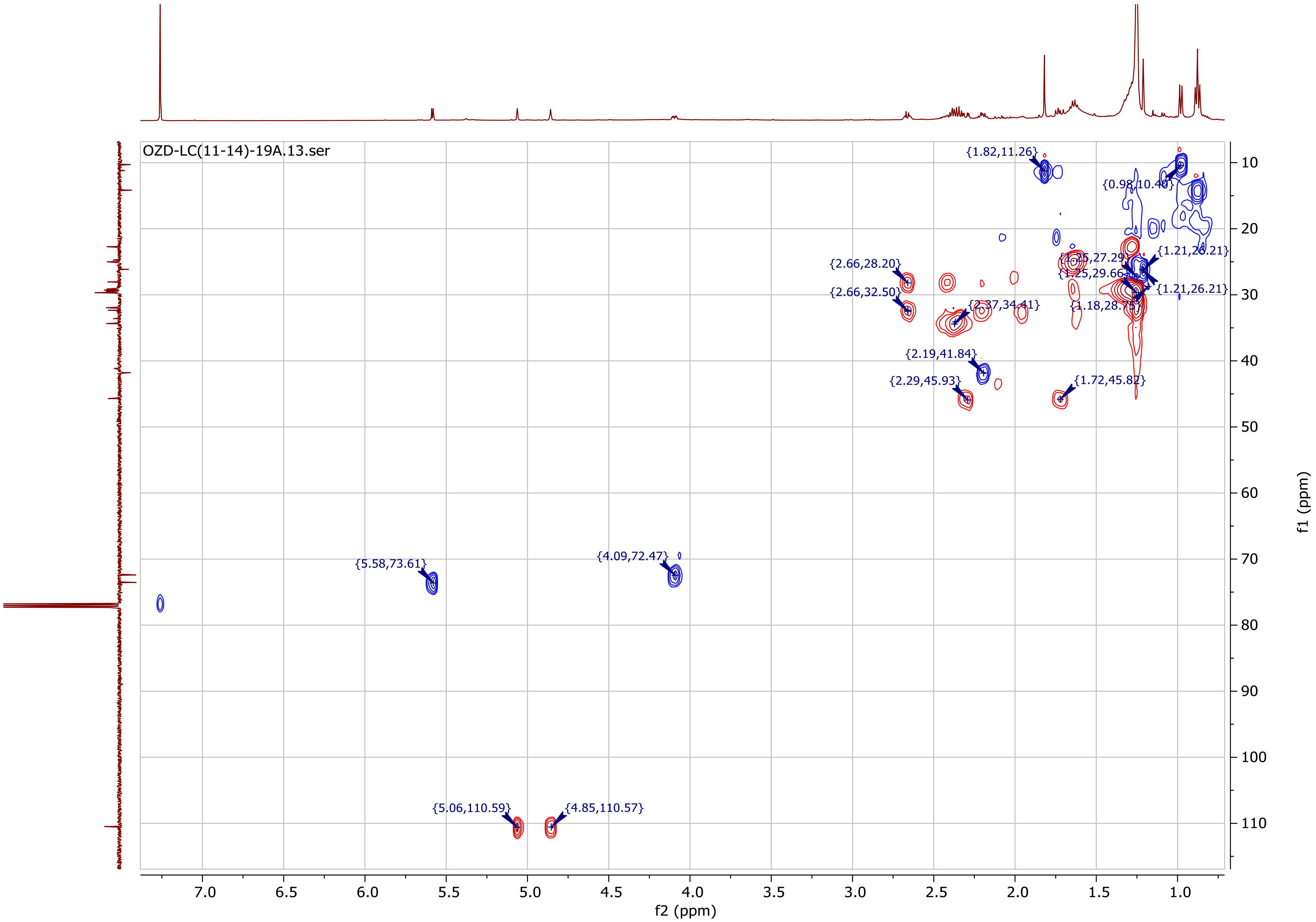

Figure S24. HSQC of Compound **3**

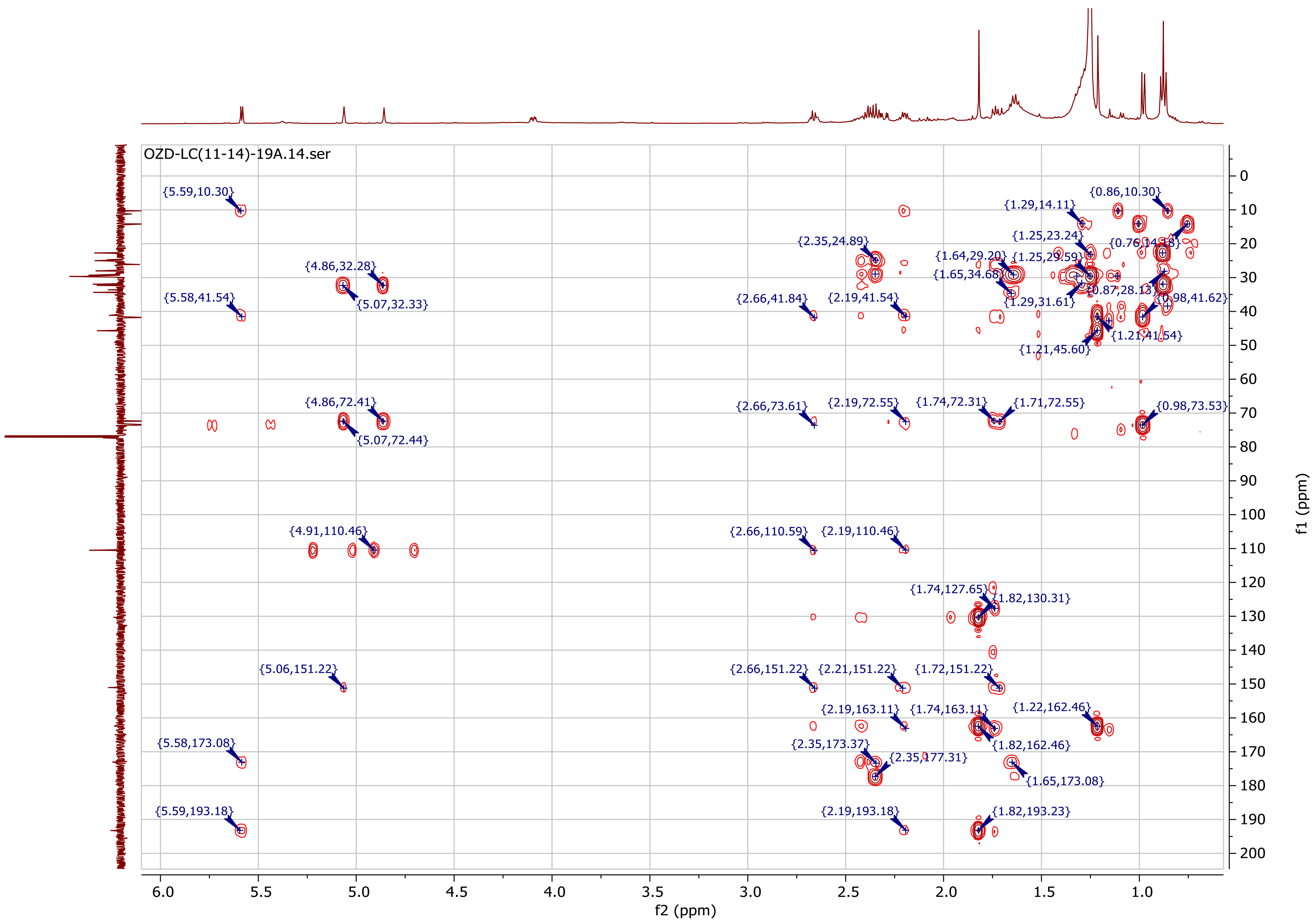

Figure S25. HMBC of Compound **3**

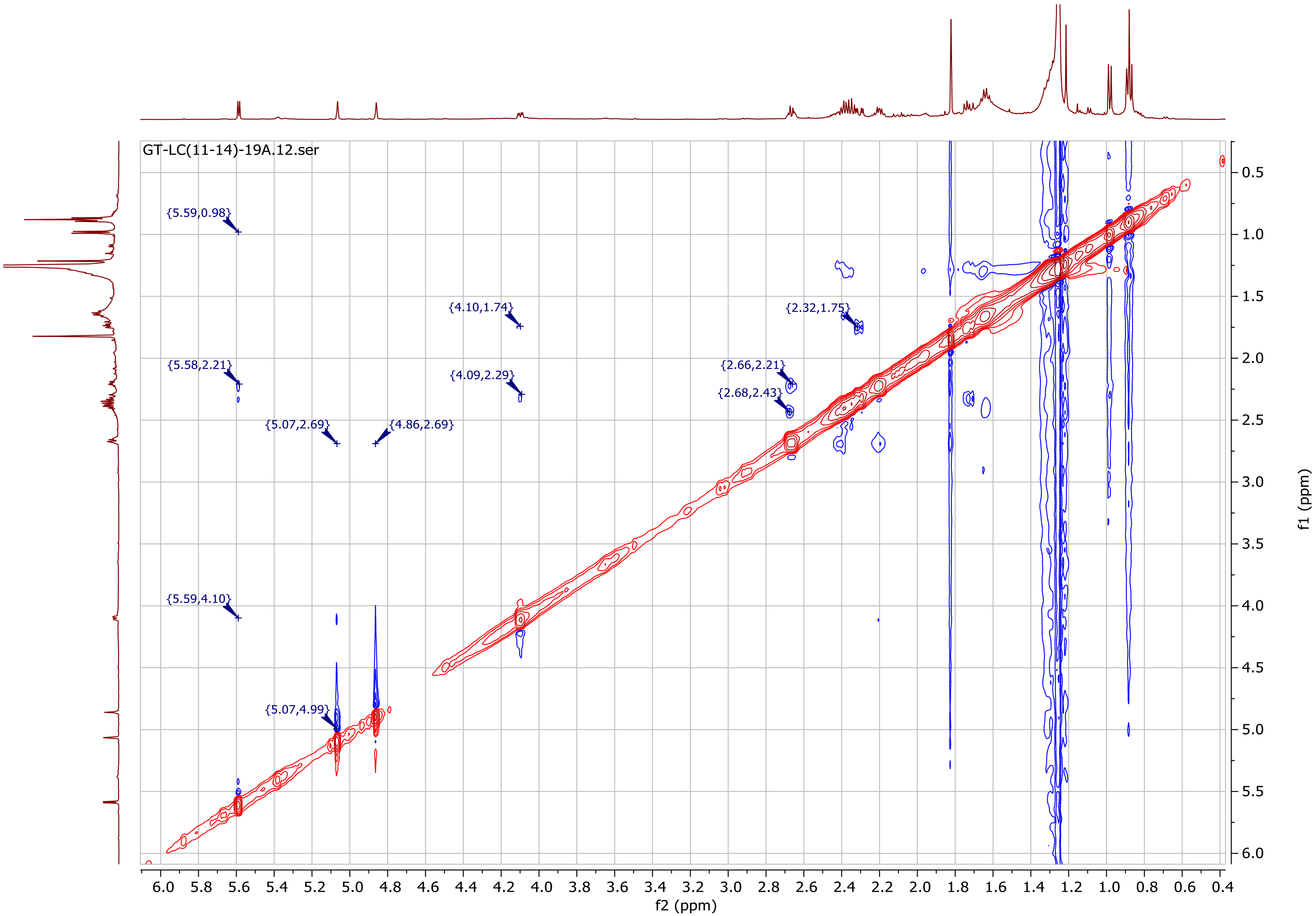

Figure S26. NOESY of Compound **3**

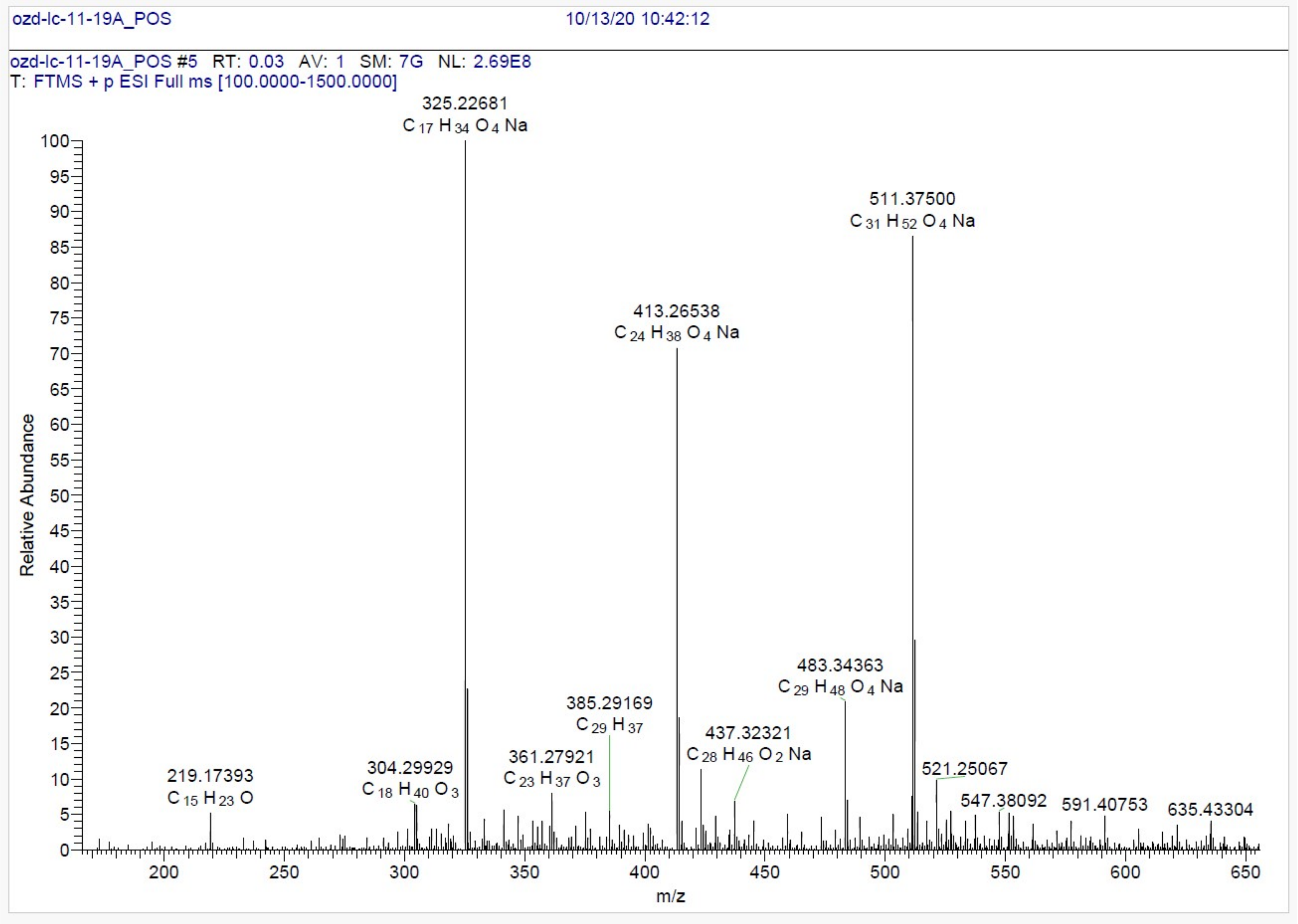

Figure S27. HRESIMS of Compound **3**

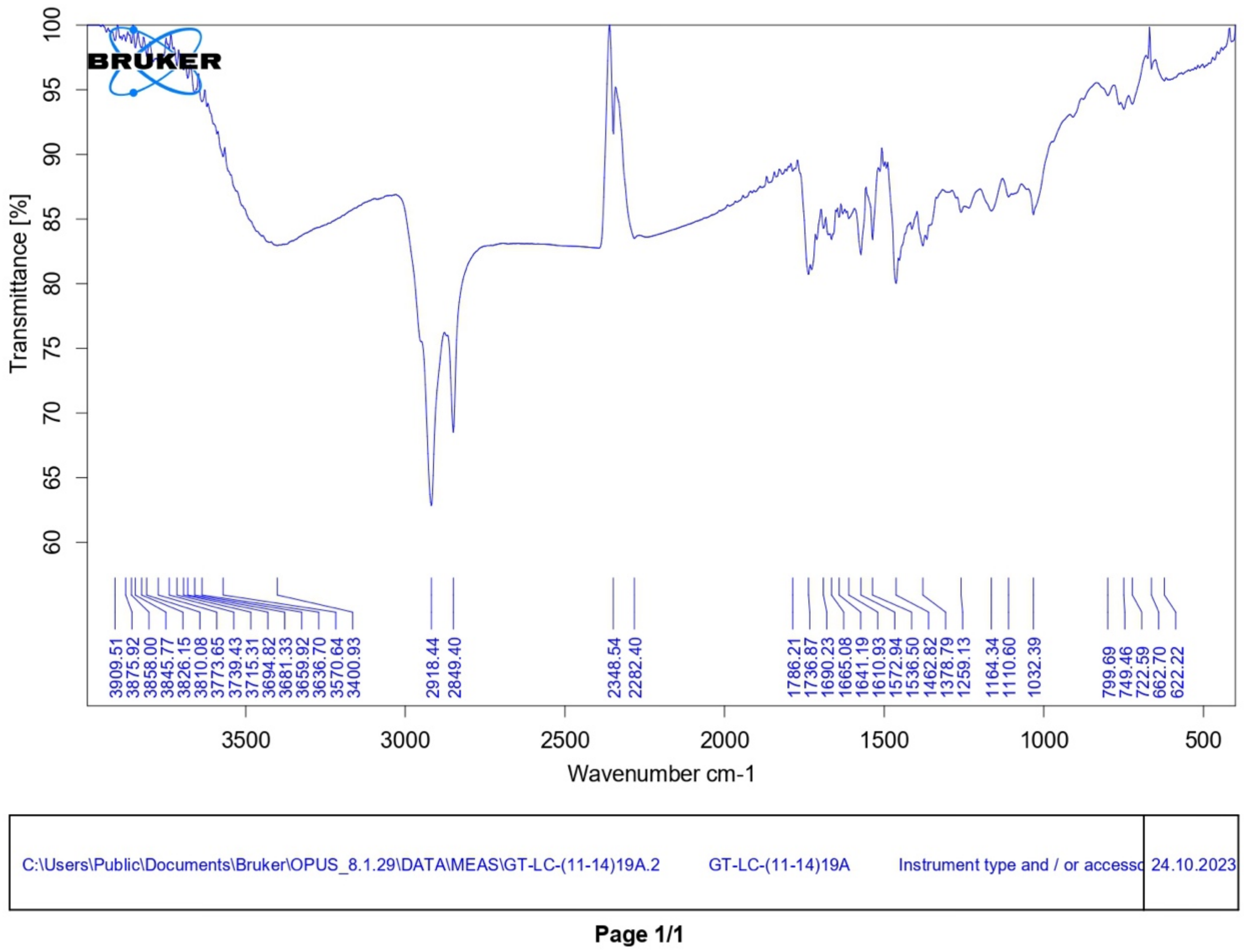

Figure S28. IR spectrum of Compound **3**

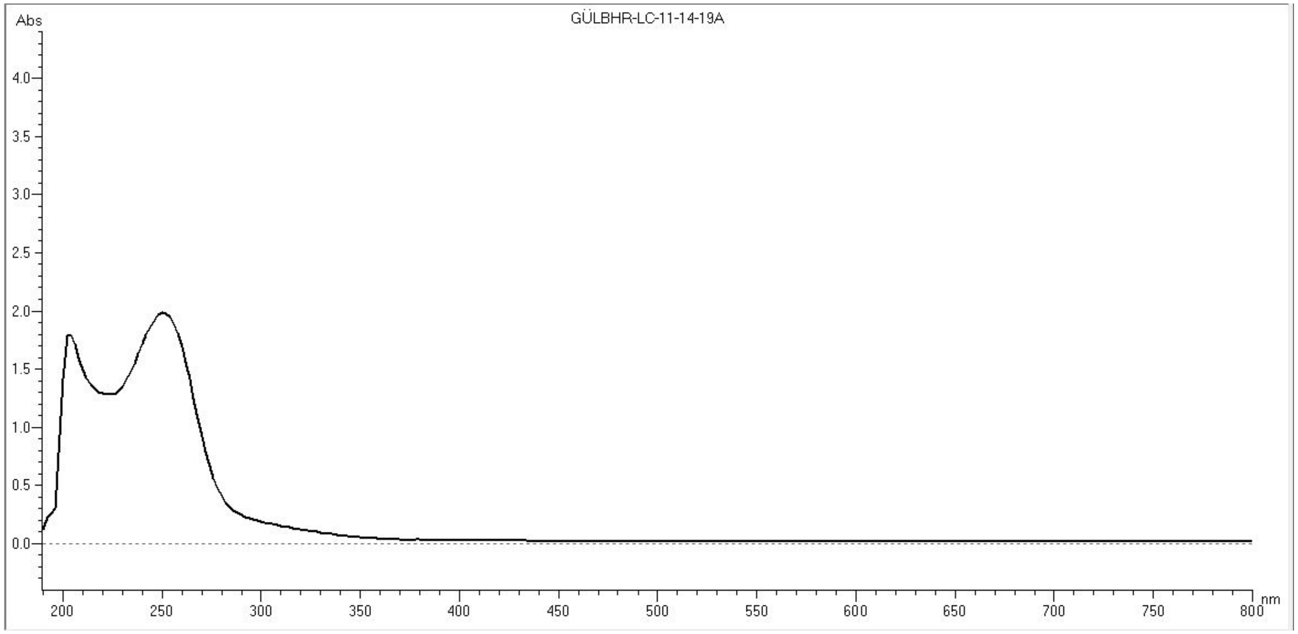

Figure S29. UV spectrum of Compound **3**

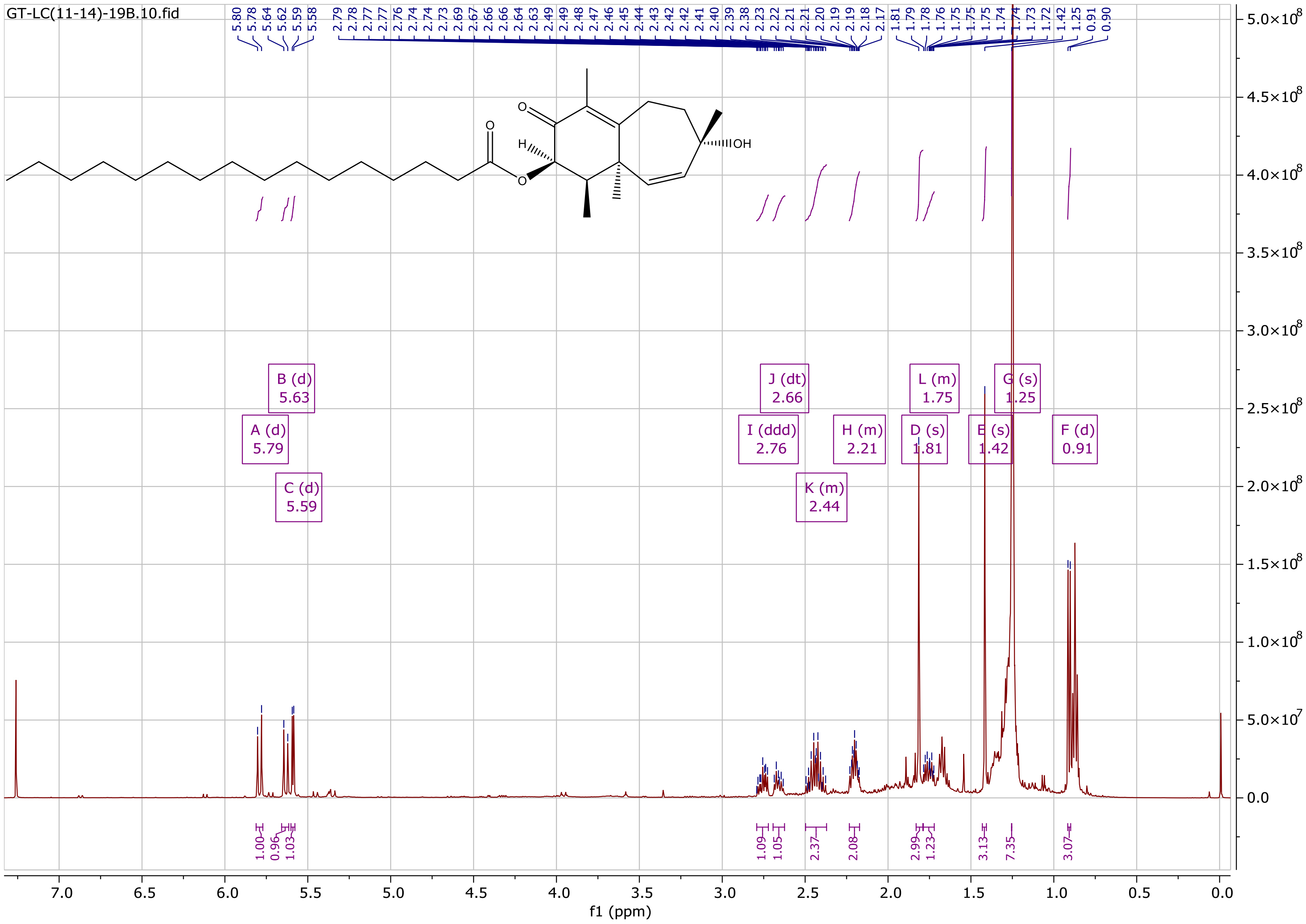

Figure S30. ^{1}H-NMR of Compound **4** (500 Hz, $CDCl_3$)

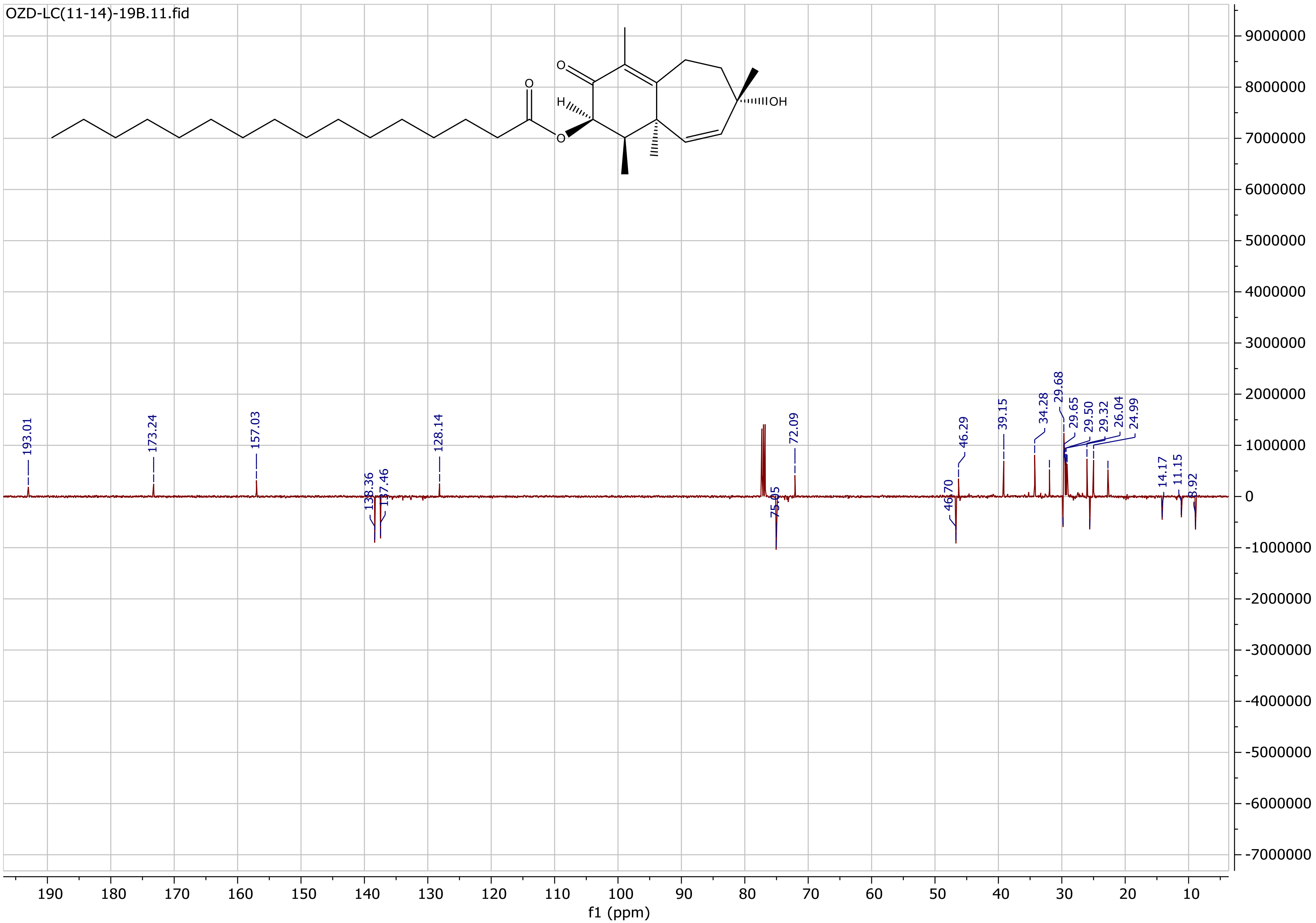

Figure S31. APT of Compound **4** (125 Hz, $CDCl_3$)

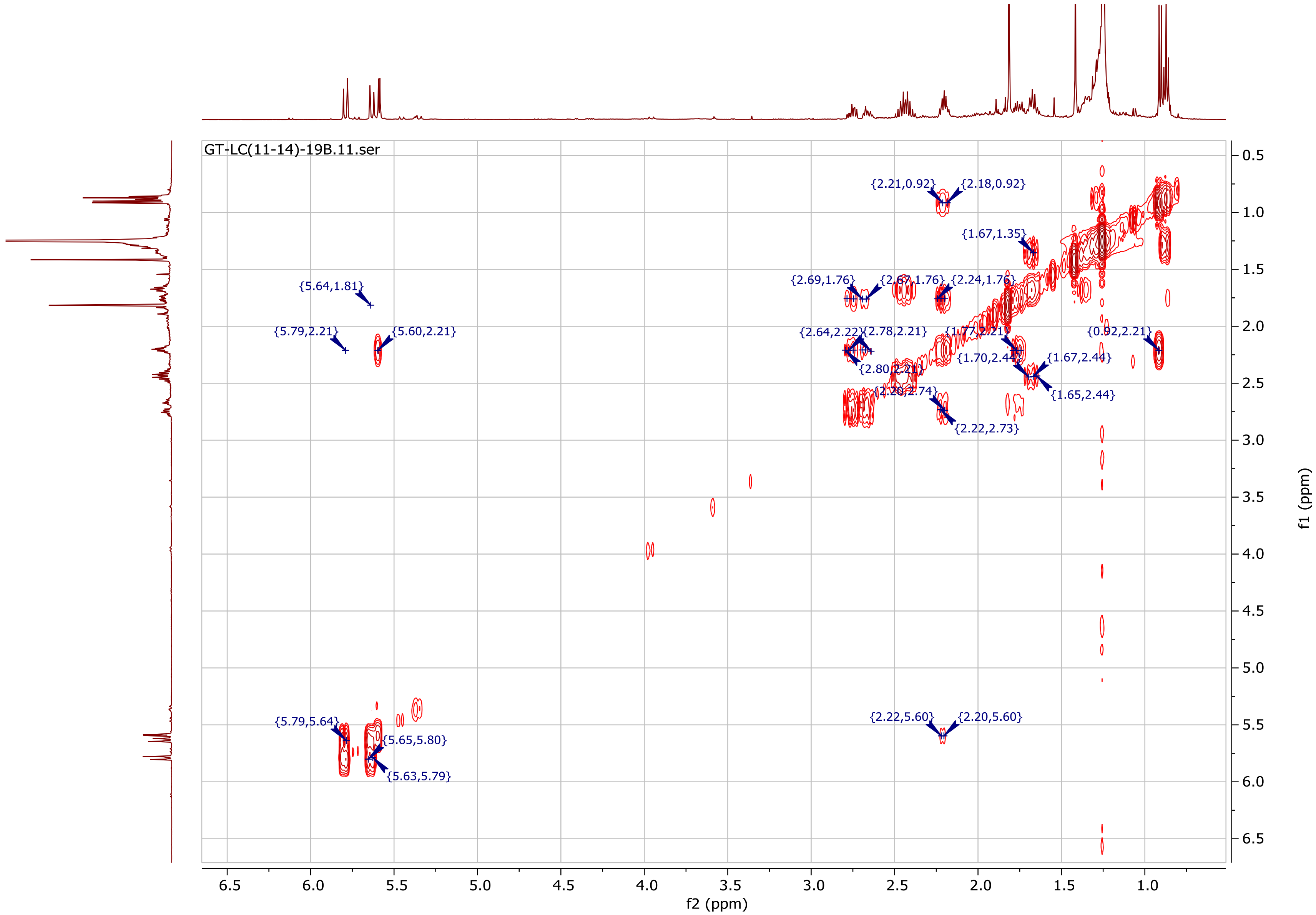

Figure S32. COSY of Compound **4**

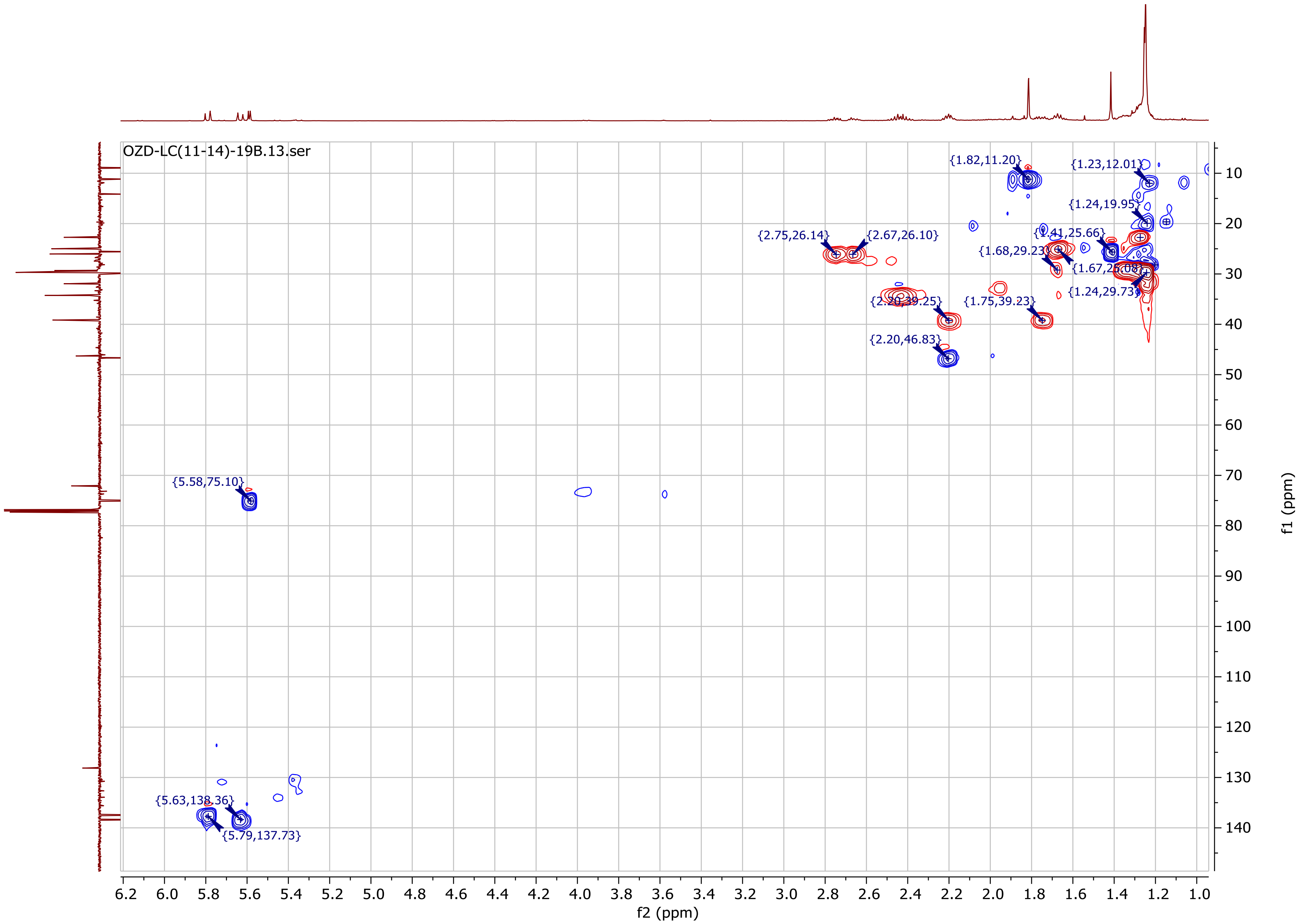

Figure S33. HSQC of Compound **4**

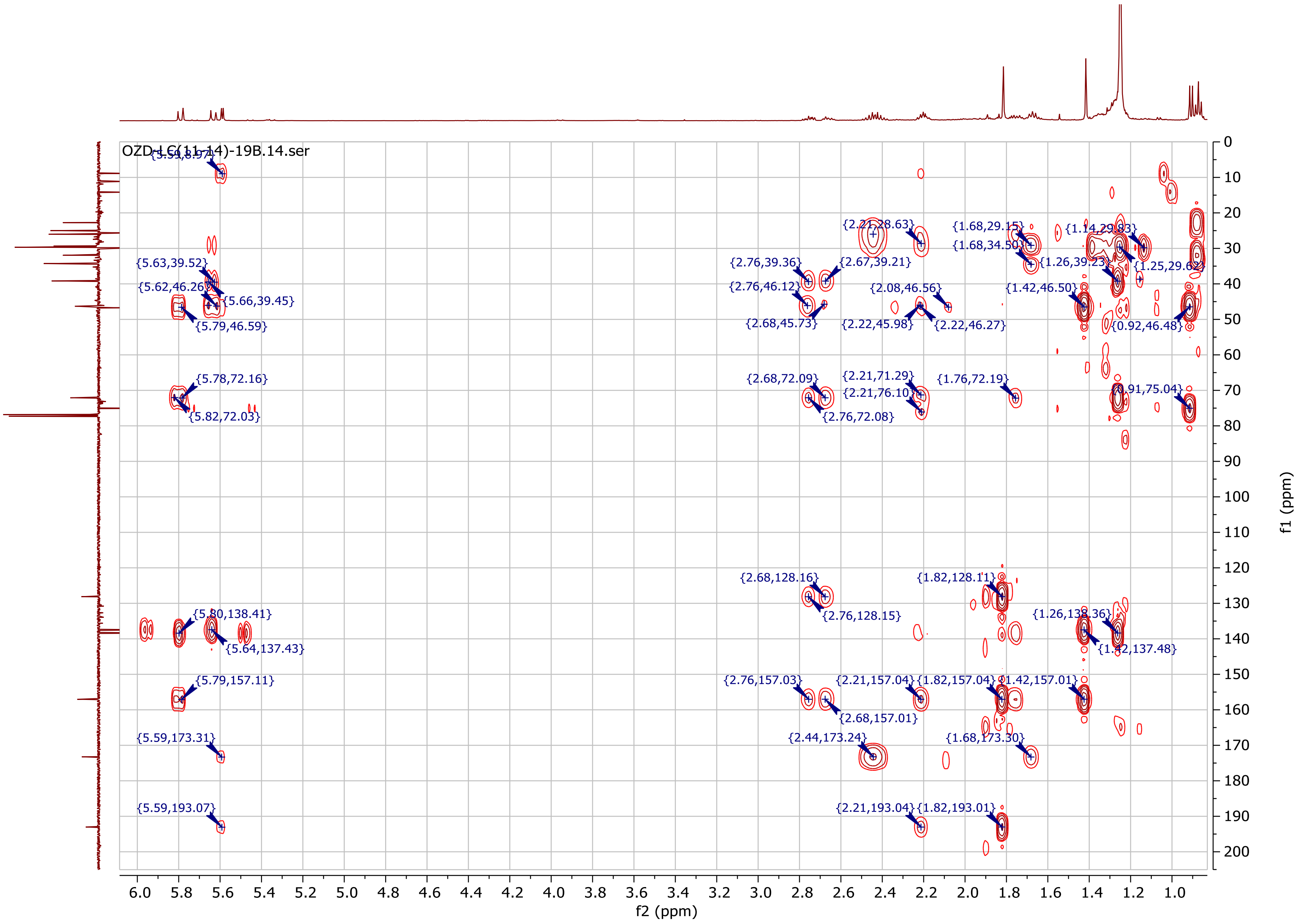

Figure S34. HMBC of Compound **4**

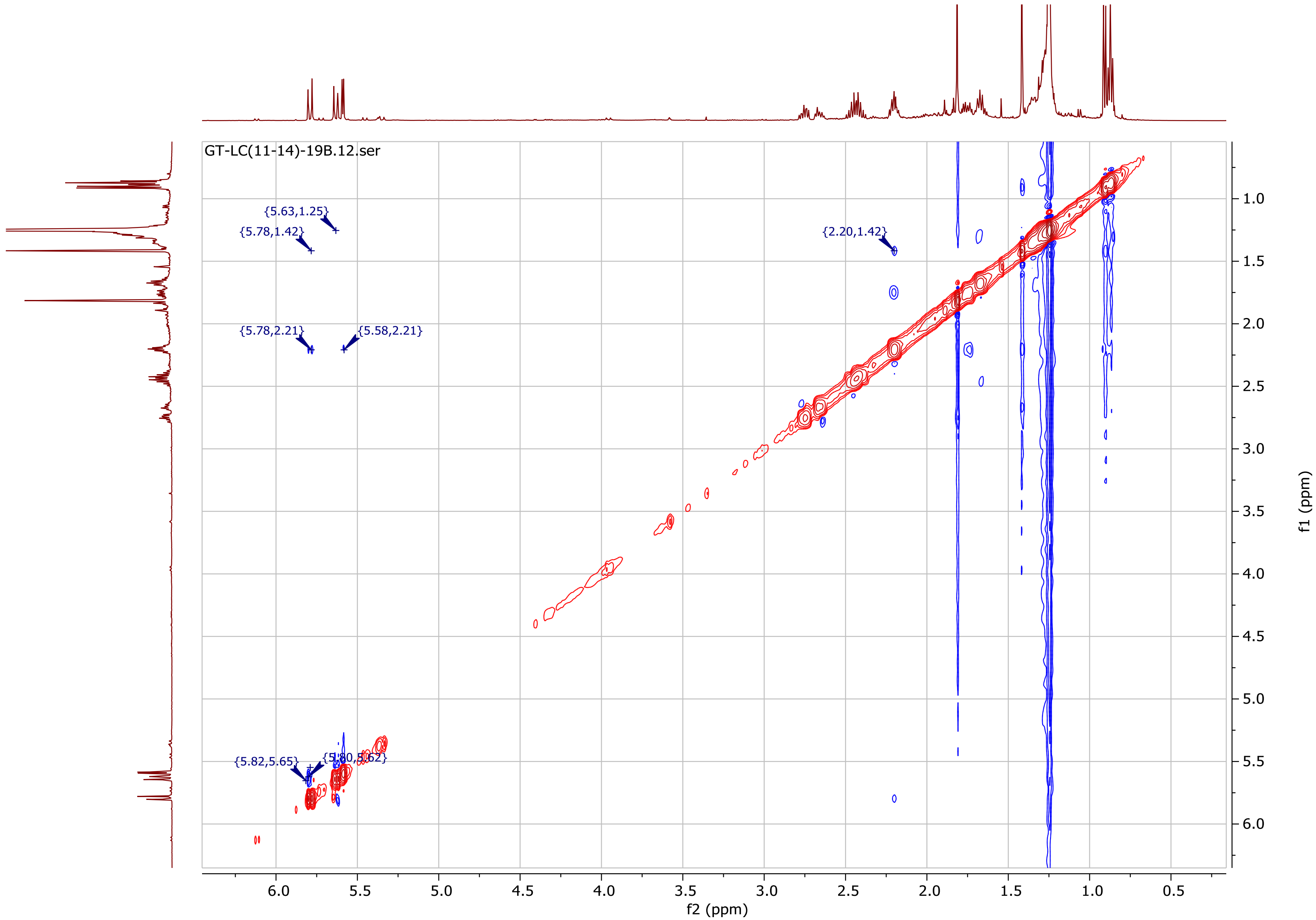

Figure S35. NOESY of Compound **4**

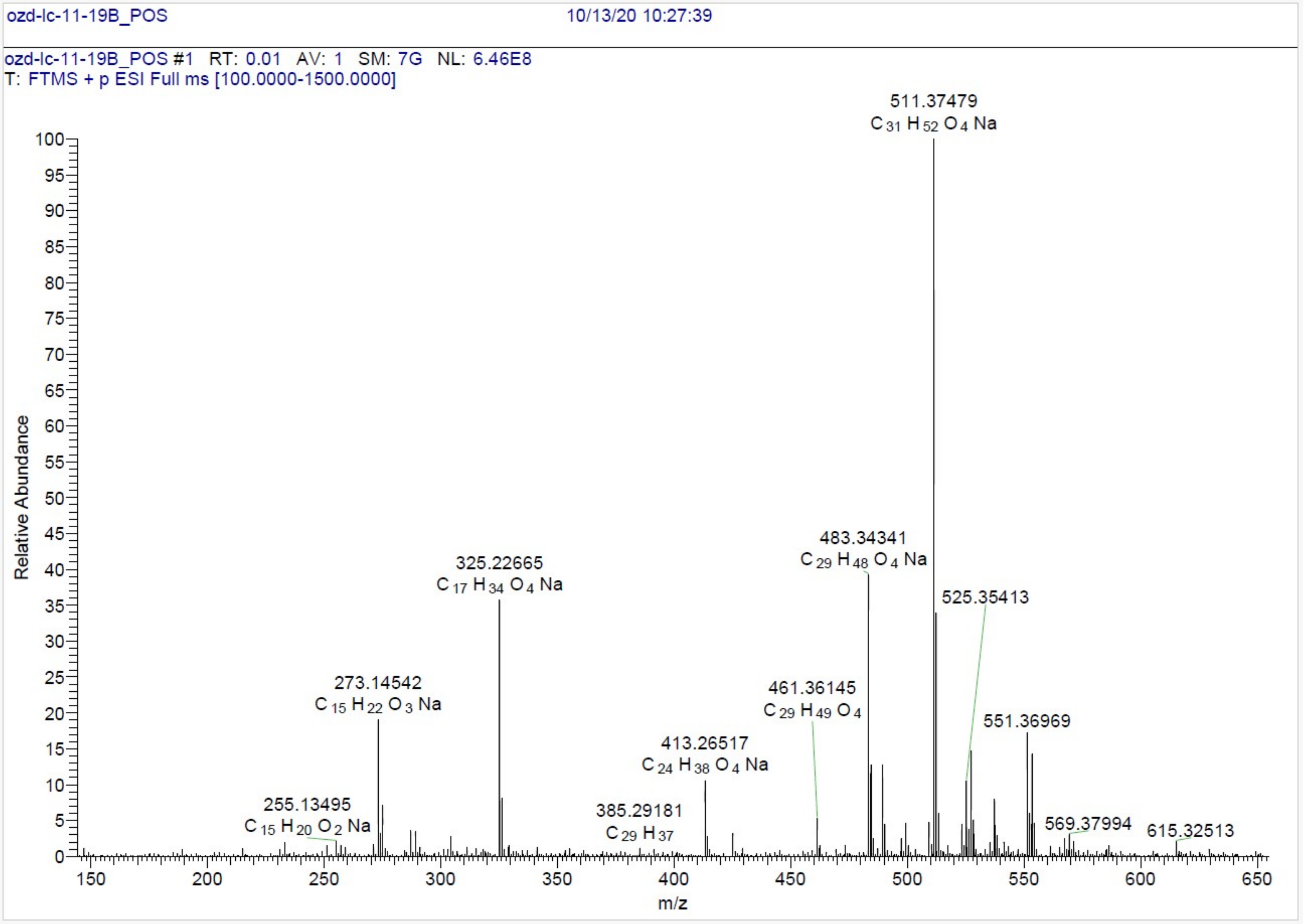

Figure S36. HRESIMS of Compound **4**

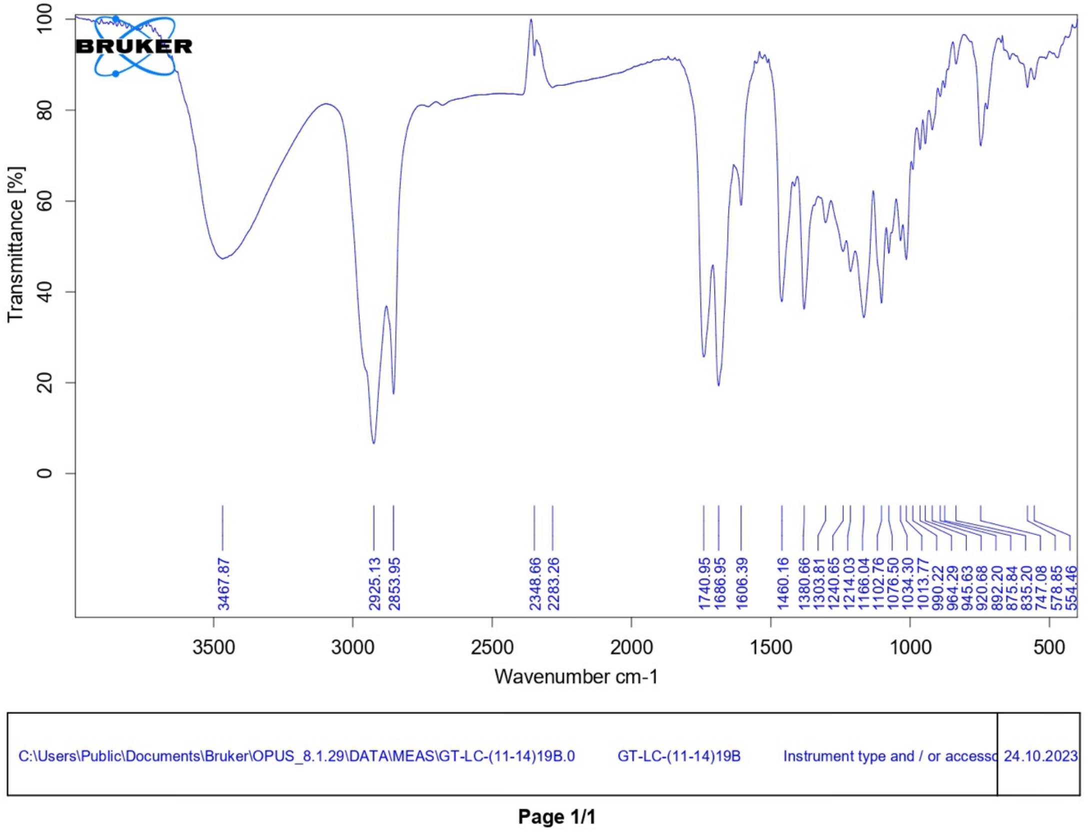

Figure S37. IR spectrum of Compound **4**

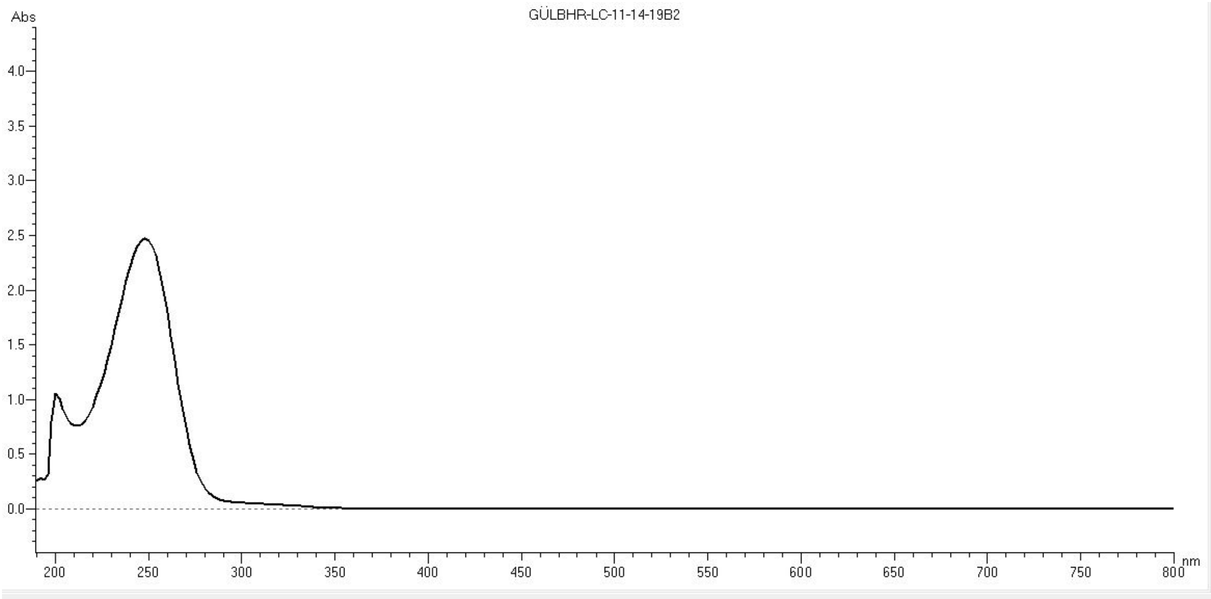

Figure S38. UV spectrum of Compound **4**

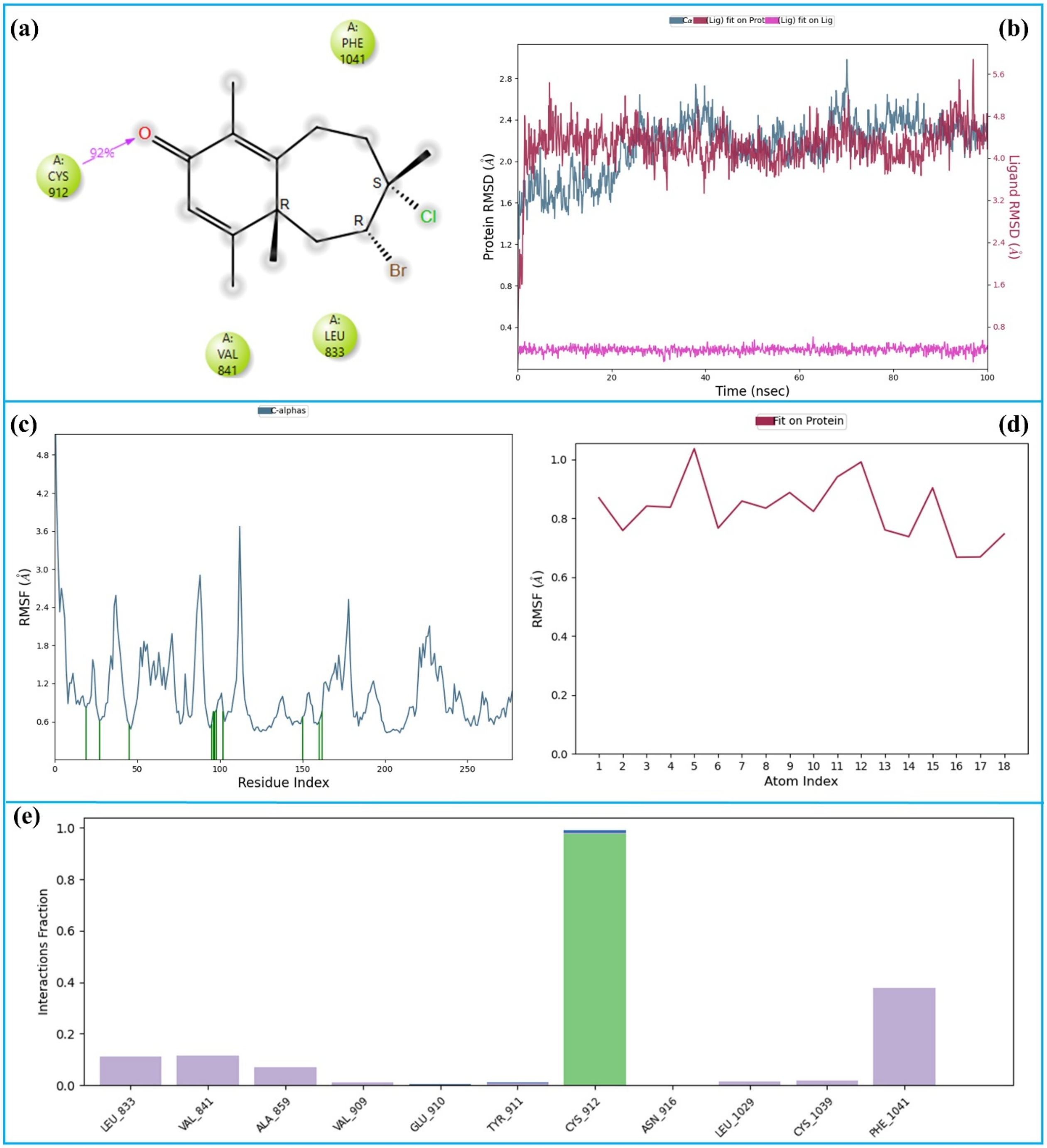

Figure S39. The 100 ns MD simulation analysis of **1-VEGFR1** complex.

(a) 2D key LPI (b) RMSD graphics (c) RMSF of Protein Cα (d) RMSF of Ligand (e) Fractional interaction histogram.

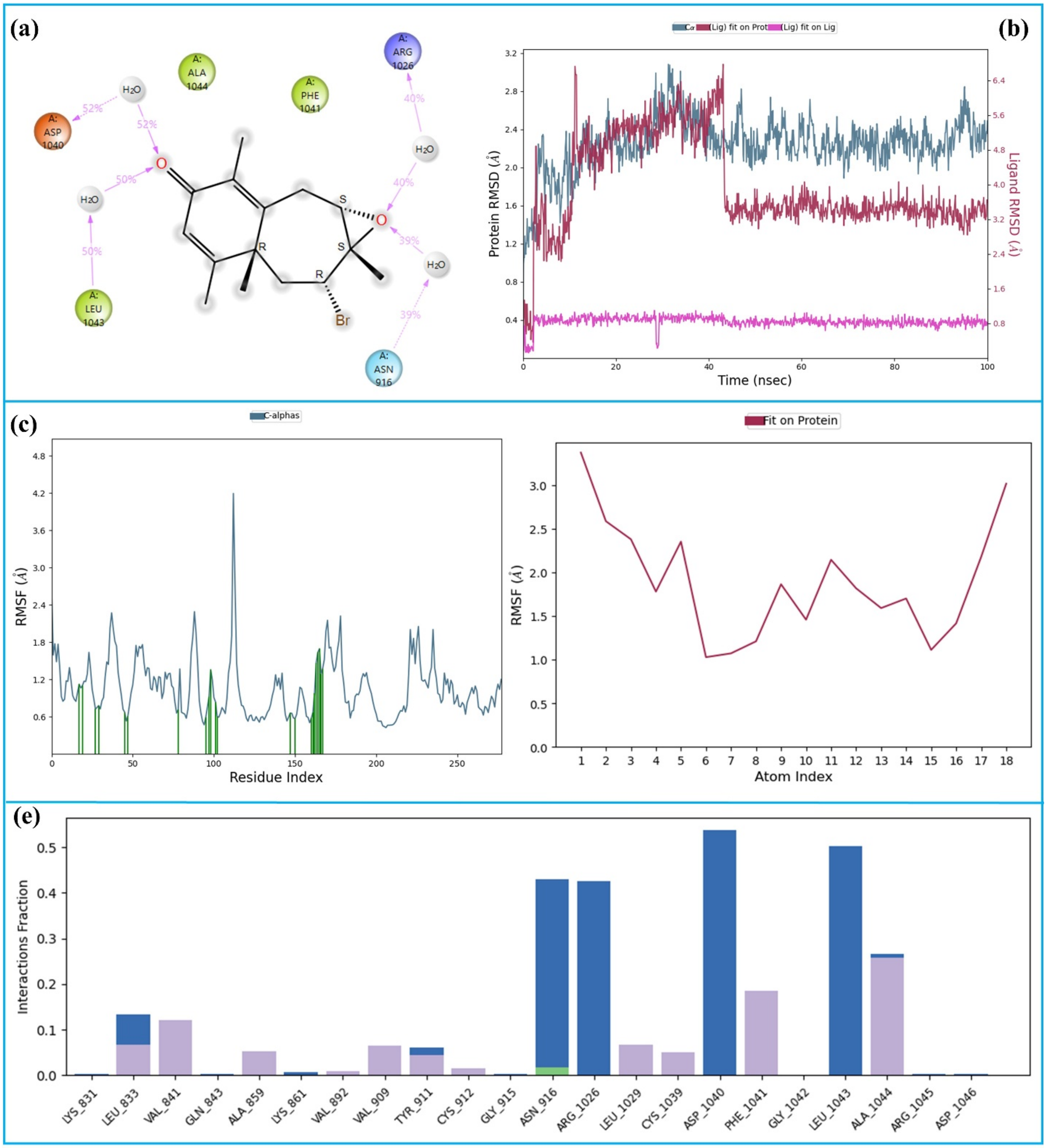

Figure S40. The 100 ns MD simulation analysis of **2-VEGFR1** complex.

(a) 2D key LPI (b) RMSD graphics (c) RMSF of Protein Cα (d) RMSF of Ligand (e) Fractional interaction histogram.

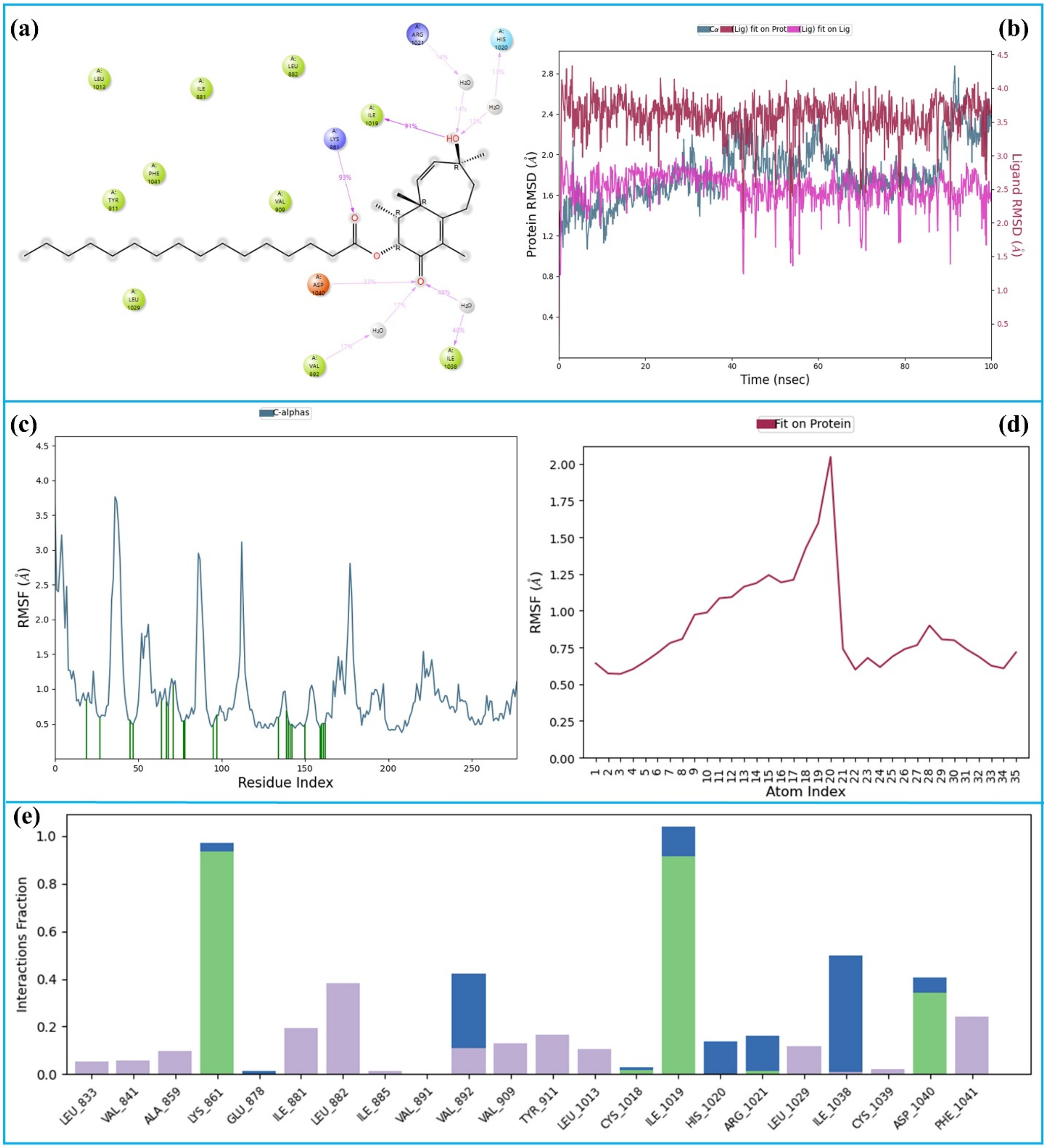

Figure S41. The 100 ns MD simulation analysis of **4-VEGFR1** complex.

(a) 2D key LPI (b) RMSD graphics (c) RMSF of Protein Cα (d) RMSF of Ligand (e) Fractional interaction histogram.

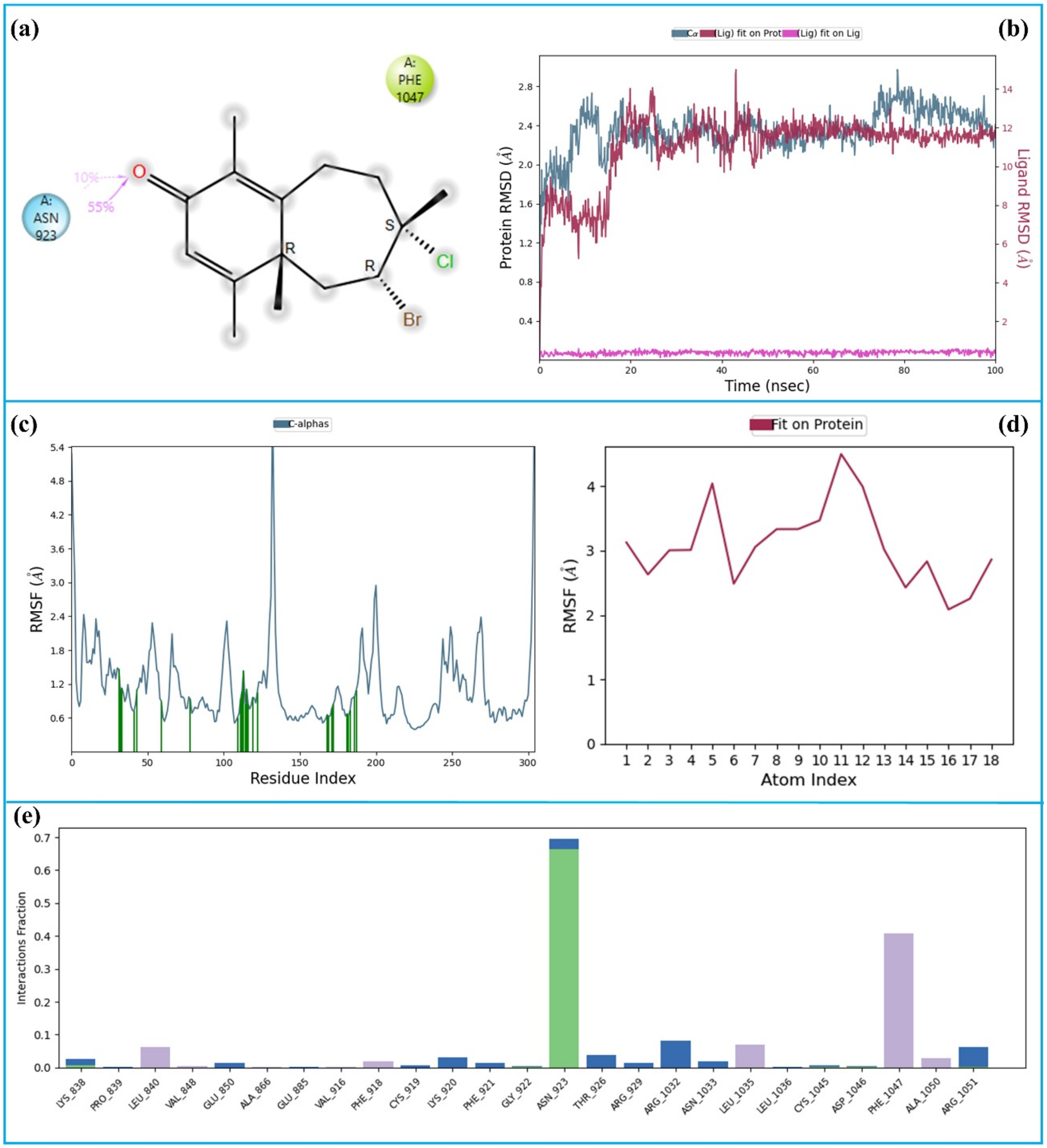

Figure S42. The 100 ns MD simulation analysis of **1-VEGFR2** complex.

(a) 2D key LPI (b) RMSD graphics (c) RMSF of Protein Cα (d) RMSF of Ligand (e) Fractional interaction histogram.

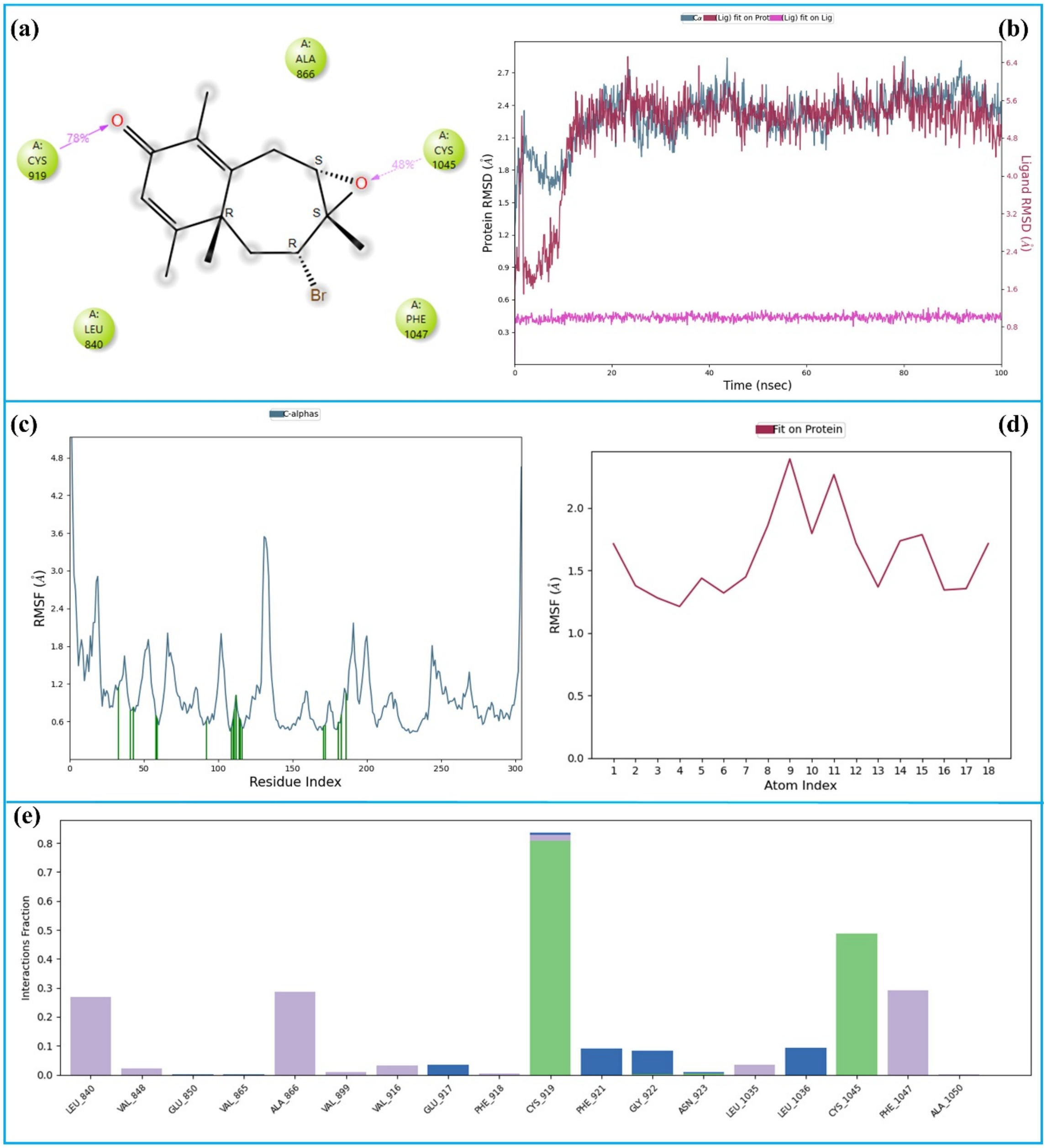

Figure S43. The 100 ns MD simulation analysis of **2-VEGFR2** complex.

(a) 2D key LPI (b) RMSD graphics (c) RMSF of Protein Cα (d) RMSF of Ligand (e) Fractional interaction histogram.

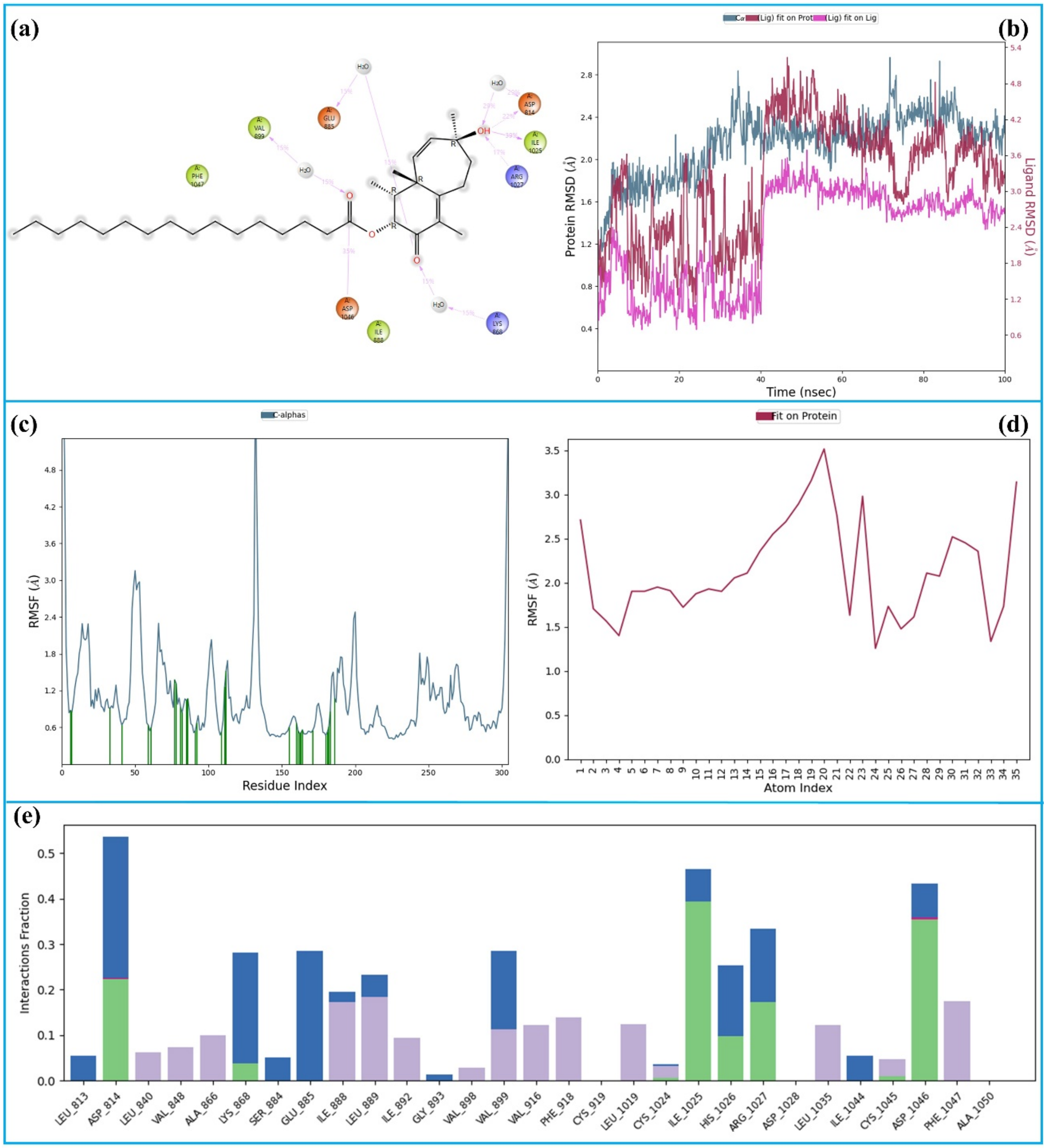

Figure S44. The 100 ns MD simulation analysis of **4-VEGFR2** complex.

(a) 2D key LPI (b) RMSD graphics (c) RMSF of Protein Cα (d) RMSF of Ligand (e) Fractional interaction histogram.

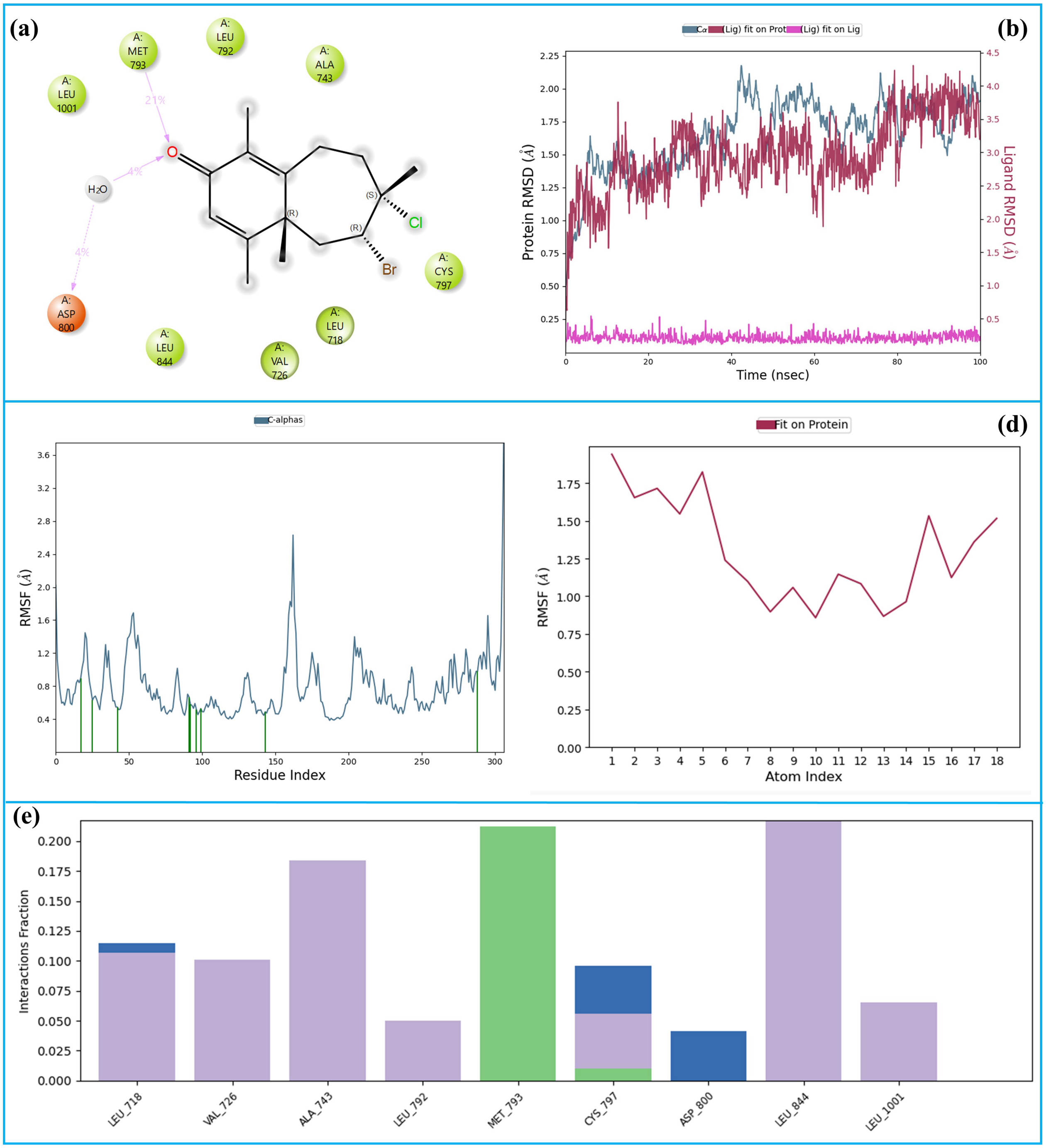

Figure S45. The 100 ns MD simulation analysis of **1-EGFR** complex.

(a) 2D key LPI (b) RMSD graphics (c) RMSF of Protein Cα (d) RMSF of Ligand (e) Fractional interaction histogram.

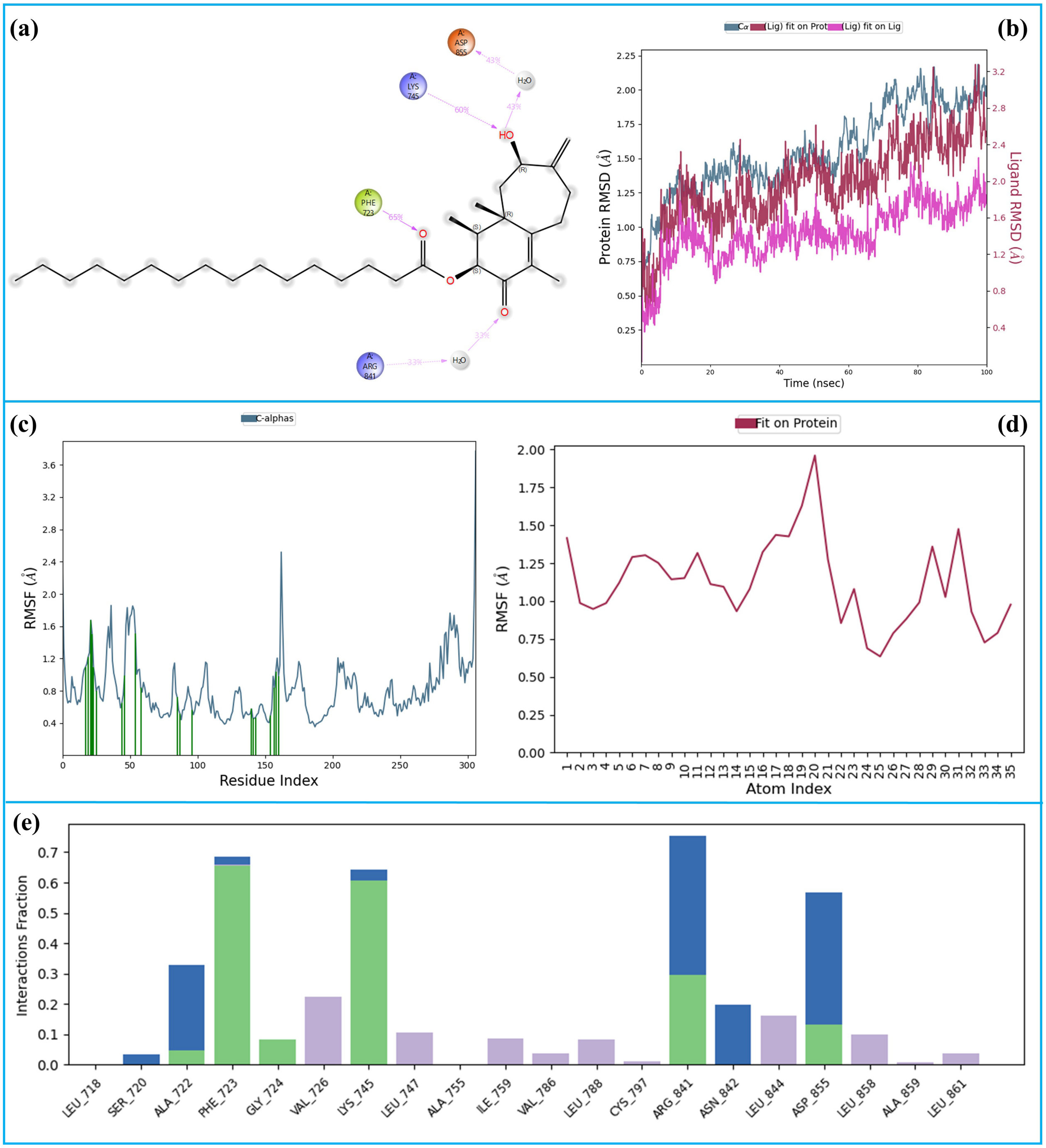

Figure S46. The 100 ns MD simulation analysis of **3-EGFR** complex.

(a) 2D key LPI (b) RMSD graphics (c) RMSF of Protein Cα (d) RMSF of Ligand (e) Fractional interaction histogram.

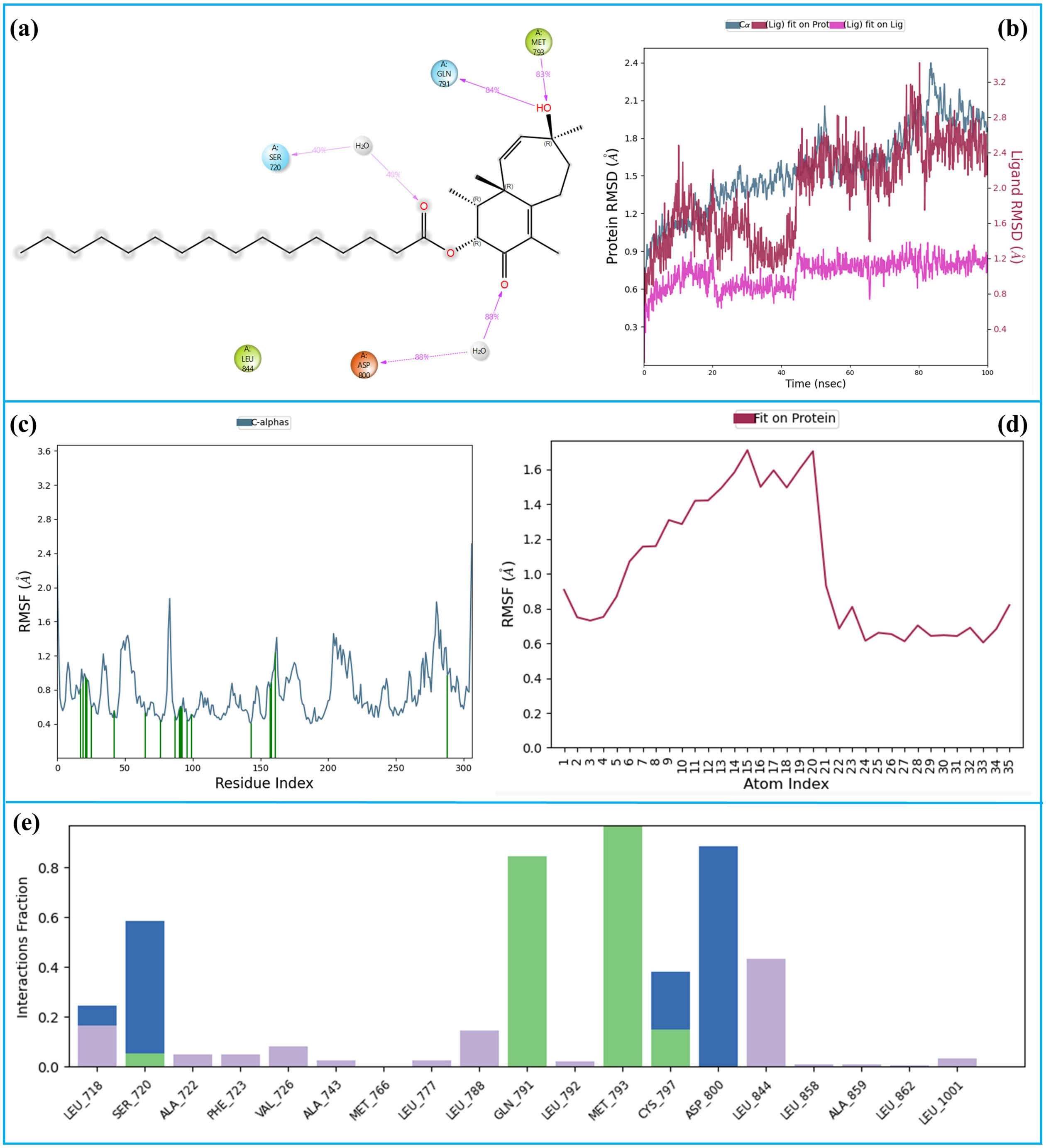

Figure S47. The 100 ns MD simulation analysis of **4-EGFR** complex.

(a) 2D key LPI (b) RMSD graphics (c) RMSF of Protein Cα (d) RMSF of Ligand (e) Fractional interaction histogram.

Table S1. NMR data of compound **1**

Position	δ_C, type	δ_H (J in Hz)	HMBC	COSY
**1**	160.5	-		
**2**	132.4	-		
**3**	185.6	-		
**4**	129.0 (CH)	6.28 (brs)	C-2, C-6, C-13	-
**5**	161.6	-	-	-
**6**	46.6	-	-	-
**7**	41.7 (CH_2)	2.84 (d, J=16.3 Hz)	C-1, C-6, C-8, C-9	H-8
		2.35 (dd, J=16.4, 10.3 Hz)		
**8**	58.9 (CH)	3.78 (d, J=10.1 Hz)	C-9, C-6, C-15	H-7
**9**	72.8	-		
**10**	44.7 (CH_2)	2.09 (ddd, J=13.5, 6.8, 1.3)	C-9, C-1	H-11
		2.68 (ddd, J=13.5, 6.8, 1.3)		
**11**	25.8 (CH_2)	2.18 (ddd, J= 13.8, 6.8, 1.8)	C-1, C-2, C-9, C-10	H-10, H-12
		2.73(ddd, J= 13.8, 6.8, 1.8)		
**12**	10.9 (CH_3)	1.94 (s)	C-1, C-2, C-3	
**13**	20.3 (CH_3)	2.11 (s)	C-4, C-5, C-6	H-14
**14**	29.7 (CH_3)	1.21 (s)	C-1, C-5, C-6, C-7	H-13, H-15
**15**	24.9 (CH_3)	1.86 (s)	C-8, C-9, C-10	H-14

Table S2. NMR data of compound **2**

Position	δ_C	δ_H (*J* in Hz)	HMBC	COSY
**1**	160.4	-	-	-
**2**	132.2	-	-	-
**3**	185.7	-	-	-
**4**	128.9	6.28 (brs)	C-2, C-6, C-13	-
**5**	161.6	-	-	-
**6**	46.7	-	-	-
**7**	41.7	2.85 (d, *J*=16.3)	C-5, C-6, C-8, C-9	H-8
		2.35 (dd, *J*=16.4, 10.3 Hz)		
**8**	58.9	3.80 (d, *J*=10.2 Hz)	C-6, C-9, C-11	H-7
**9**	72.9	-	-	-
**10**	61.1	2.82 (dd, *J*=6.5, 1.0 Hz)	C-1, C-6, C-8, C-9	H-11
**11**	25.8	2.19 (dd, *J*=13.1, 1.0 Hz)	C-6, C-9	H-10
		2.75 (dd, *J*=13.1, 6.5 Hz)		
**12**	11.9	1.92 (s)	C-1, C-2, C-3	-
**13**	19.5	2.10 (s)	C-4, C-5, C-6	-
**14**	26.6	1.20 (s)	C-5, C-6, C-7	-
**15**	24.9	1.85 (s)	C-8, C-9	H-10

Table S3. NMR data of compound **3**

Position	δ_C	δ_H (*J* in Hz)	HMBC	COSY
**1**	162.4	-	-	
**2**	130.2	-	-	
**3**	193.2	-	-	
**4**	73.5	5.58 (d, *J*=4.7 Hz)	C-3, C-5, C-13, C-16	H-5
**5**	41.7	2.20, m	C-3, C-4, C-6, C-13	H-4
**6**	41.1	-	-	-
**7**	45.7	1.73 (dd, *J*=14.3, 9.2 Hz)	C-1, C-6, C-8	H-7b, H-8
		2.31 (dd, *J*=14.3, 3.8 Hz)		H-7a
**8**	72.4	4.10 (dd, *J*=9.2, 3.8 Hz)	C-5, C-6	H-7
**9**	151.0	-	-	
**10**	32.2	2.23, m	C-1, C-8, C-9, C-15	H-11
		2.68, m, ovl.		
**11**	28.1	2.42,m	C-1, C-2, C-6	H-10
		2.68, m		
**12**	11.1	1.22 (s)	C-1, C-2, C-3	-
**13**	10.2	1.00 (d, 6.9 Hz)	C-4, C-6	-
**14**	26.2	1.82 (s)	C-1, C-6, C-7	-
**15**	110.6	4.86 (d, *J*=1.5 Hz)	C-8, C-10	H-15b
		5.06 (d, *J*=1.5 Hz)		H-15a
**16**	173.1	-	-	-
**17**	34.0	2.37, m	C-16	H-18
**18**	24.7	1.66, m	C-16, C-17, C-18	H-17
**19-28**	29.2	1.28, m, ovl.	-	H-17, H-29
**29**	32.1	1.25, m	C-28	-
**30**	22.7	1.29, m, ovl.	C-31	H-31
**31**	14.2	0.88 (t, *J*=6.6 Hz)	C-29, C-30	H-30

Table S4. NMR data of compound **4**

Position	δ_C	δ_H (J in Hz)	HMBC	COSY
**1**	157.0	-	-	-
**2**	128.1	-	-	-
**3**	192.9	-	-	-
**4**	75.0	5.59 (d, J=4.5 Hz)	C-13	H-5
**5**	46.7	2.23, m	C-3, C-4, C-6	H-4
**6**	46.2	-	-	-
**7**	137.4	5.79 (d, J=12.0 Hz)	C-1, C-5, C-9	H-8
**8**	138.3	5.63 (d, J=12.0 Hz)	C-6, C-10, C-15	H-7
**9**	72.0	-	-	-
**10**	39.1	1.76, m	C-1, C-9, C-11	H-10b, H-11
		2.23, m		H-10a
**11**	26.0	2.75, m	C-1, C-2, C-9, C-10	H-10
		2.67, m		
**12**	11.1	1.83 (s)	C-1, C-2, C-3	-
**13**	8.9	0.93 (s)	C-4, C-6	-
**14**	25.6	1.44 (s)	C-1, C-5, C-7,	-
**15**	29.3	1.27 (s)	C-9, C-10	-
**16**	173.2	-	-	-
**17**	34.2	2.44, m	C-16	H-18
**18**	24.9	1.69, m	C-16, C-17, C-18	H-17
**19-28**	29.2	1.28, m, ovlp.	-	-
**29**	32.1	1.25, m	C-28	-
**30**	22.7	1.29, m, ovlp.	C-31	H-31
**31**	14.1	0.88, t, 6.7 Hz	C-29, C-30	H-30

Table S5. Impurity of the Compound **1** (LC(11-14)-5HB) by qHNMR

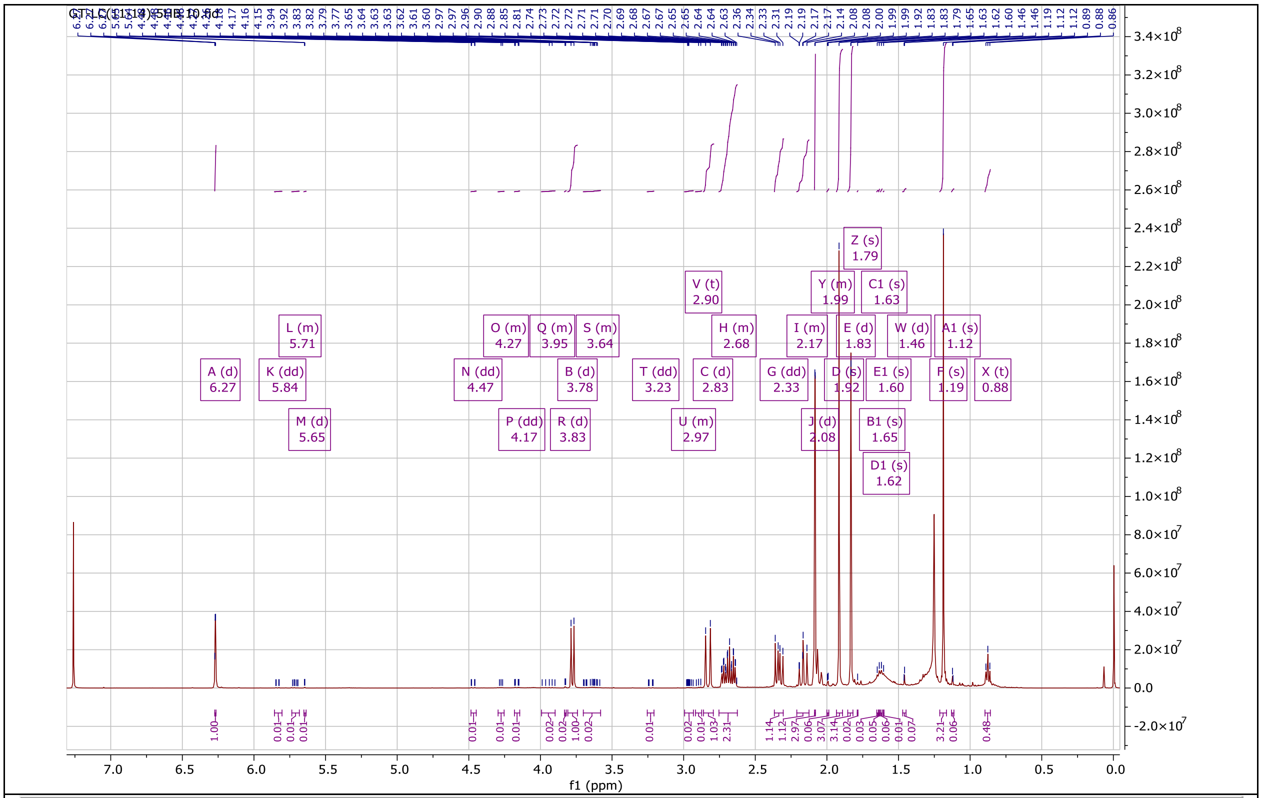

**Multiplets** (Compound **1**)							
	Shift	Range	Name	H's	Integral	Class	J's
**1**	6.269	6.27 .. 6.27	A (d)	1	1.000	d	1.53
**2**	5.836	5.86 .. 5.80	K (dd)	0	0.009	dd	1.25, 10.36
**3**	5.713	5.74 .. 5.68	L (m)	0	0.011	m	
**4**	5.646	5.65 .. 5.64	M (d)	0	0.009	d	1.42
**5**	4.470	4.48 .. 4.45	N (dd)	0	0.012	dd	1.34, 11.70
**6**	4.275	4.30 .. 4.25	O (m)	0	0.008	m	
**7**	4.165	4.18 .. 4.14	P (dd)	0	0.013	dd	2.52, 12.46
**8**	3.952	3.99 .. 3.90	Q (m)	0	0.019	m	
**9**	3.826	3.83 .. 3.82	R (d)	0	0.020	d	2.36
**10**	3.776	3.81 .. 3.74	B (d)	1	1.005	d	10.14
**11**	3.635	3.70 .. 3.58	S (m)	0	0.025	m	
**12**	3.230	3.25 .. 3.21	T (dd)	0	0.011	dd	2.46, 13.98
**13**	2.966	2.99 .. 2.93	U (m)	0	0.020	m	
**14**	2.896	2.92 .. 2.87	V (t)	0	0.013	t	7.77, 7.77
**15**	2.830	2.86 .. 2.79	C (d)	1	1.029	d	16.35
**16**	2.683	2.75 .. 2.63	H (m)	2	2.309	m	
**17**	2.334	2.37 .. 2.30	G (dd)	1	1.141	dd	10.26, 16.43
**18**	2.173	2.21 .. 2.12	I (m)	1	1.116	m	
**19**	2.083	2.09 .. 2.08	J (d)	3	2.968	d	1.31

**20**	1.994	2.00 .. 1.99	Y (m)	0	0.063	m	
**21**	1.915	1.93 .. 1.89	D (s)	3	3.072	s	
**17**	2.334	2.37 .. 2.30	G (dd)	1	1.141	dd	10.26, 16.43
**18**	2.173	2.21 .. 2.12	I (m)	1	1.116	m	
**19**	2.083	2.09 .. 2.08	J (d)	3	2.968	d	1.31
**20**	1.994	2.00 .. 1.99	Y (m)	0	0.063	m	
**21**	1.915	1.93 .. 1.89	D (s)	3	3.072	s	
**22**	1.832	1.85 .. 1.82	E (d)	3	3.138	d	0.95
**23**	1.774	1.79 .. 1.76	Z (d)	0	0.022	d	11.34
**24**	1.649	1.65 .. 1.64	B1 (s)	0	0.029	s	
**25**	1.634	1.64 .. 1.63	C1 (s)	0	0.055	s	
**26**	1.619	1.63 .. 1.61	D1 (s)	0	0.060	s	
**27**	1.604	1.61 .. 1.60	E1 (s)	0	0.013	s	
**28**	1.459	1.47 .. 1.45	W (d)	0	0.066	d	1.77
**29**	1.187	1.21 .. 1.17	F (s)	3	3.211	s	
**30**	1.123	1.13 .. 1.11	A1 (s)	0	0.061	s	
**31**	0.876	0.90 .. 0.86	X (t)	0	0.479	t	6.92, 6.92
				**total**	**21,007**		
				**analyte signals**	**19,989**		
				**%**	**95,154**		

Table S6. Impurity of the Compound **2** (LC(11-14)-8B) by qHNMR

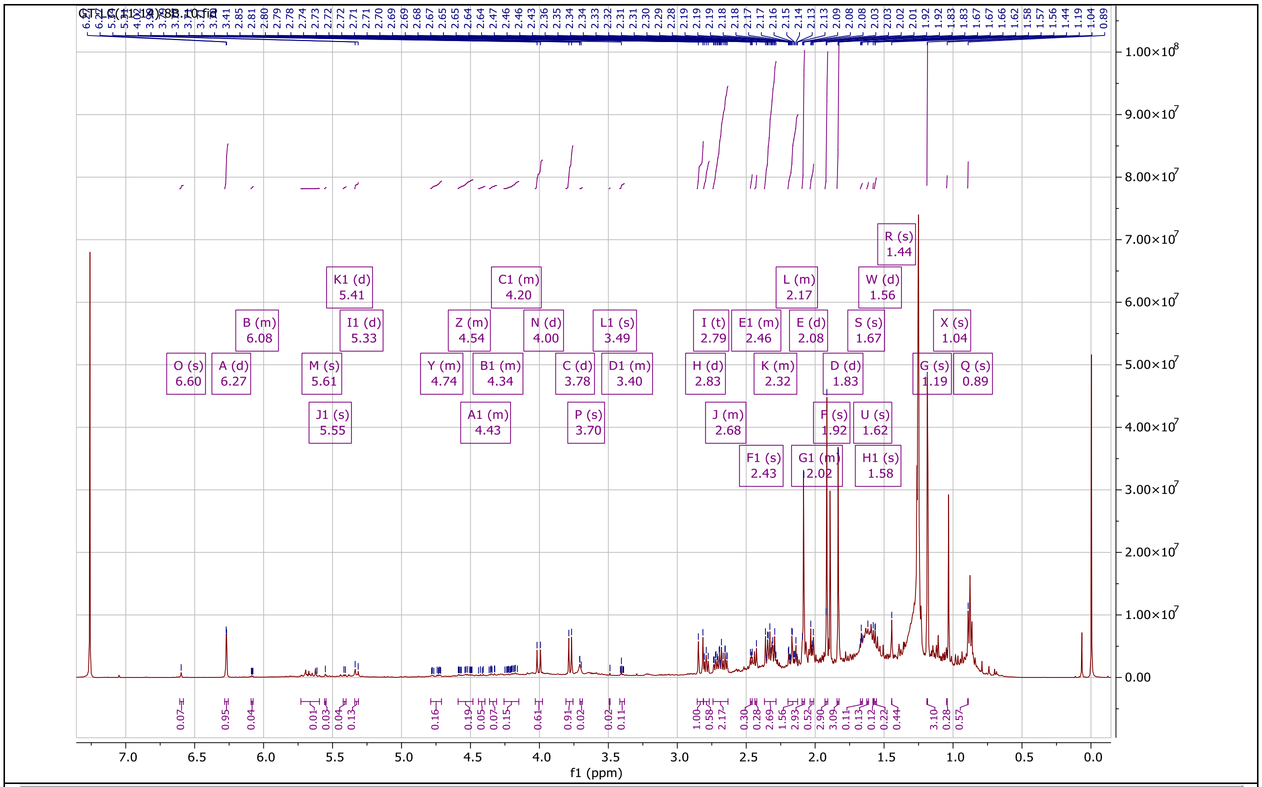

**Multiplets** (Compound **2**)							
	Shift	Range	Name	H's	Integral	Class	J's
1	6.598	6.61 .. 6.58	O (s)	0	**0.070**	s	
2	6.270	6.28 .. 6.26	A (d)	1	0.949	d	1.46
3	6.083	6.09 .. 6.08	B (m)	0	**0.045**	m	
4	5.613	5.73 .. 5.60	M (s)	0	**0.006**	s	
5	5.552	5.56 .. 5.55	J1 (s)	0	**0.025**	s	
6	5.412	5.42 .. 5.40	K1 (d)	0	**0.043**	d	5.39
7	5.325	5.34 .. 5.31	I1 (d)	0	**0.131**	d	10.99
9	4.425	4.44 .. 4.40	A1 (m)	0	**0.049**	m	
10	4.336	4.36 .. 4.31	B1 (m)	0	**0.068**	m	
11	4.203	4.26 .. 4.15	C1 (m)	0	**0.154**	m	
12	4.004	4.03 .. 3.98	N (d)	1	0.605	d	12.43
13	3.777	3.81 .. 3.76	C (d)	1	0.907	d	10.14
14	3.696	3.71 .. 3.69	P (s)	0	**0.021**	s	
15	3.488	3.49 .. 3.49	L1 (s)	0	**0.018**	s	
16	3.399	3.42 .. 3.38	D1 (m)	0	**0.106**	m	
17	2.831	2.86 .. 2.81	H (d)	1	1.000	d	16.40
18	2.790	2.81 .. 2.77	I (t)	1	0.580	t	7.08, 7.08
19	2.684	2.74 .. 2.63	J (m)	2	2.169	m	
20	2.324	2.37 .. 2.28	K (m)	3	2.690	m	

21	2.168	2.20 .. 2.13	L (m)	2	1.562	m	
22	2.083	2.10 .. 2.08	E (d)	3	2.928	d	1.32
23	1.916	1.93 .. 1.91	F (s)	3	2.899	s	
24	1.833	1.84 .. 1.83	D (d)	3	3.085	d	0.81
25	1.666	1.67 .. 1.66	S (s)	0	**0.106**	s	
26	1.618	1.63 .. 1.61	U (s)	0	**0.134**	s	
27	1.579	1.58 .. 1.58	H1 (s)	0	**0.118**	s	
28	1.187	1.19 .. 1.18	G (s)	3	3.099	s	
				**total**	**23,567**		
				**analyte signals**	**22,473**		
				**%**	**95,358**		

Table S7. Impurity of the compound **3** (LC(11-14)-19A) by Qhnmr

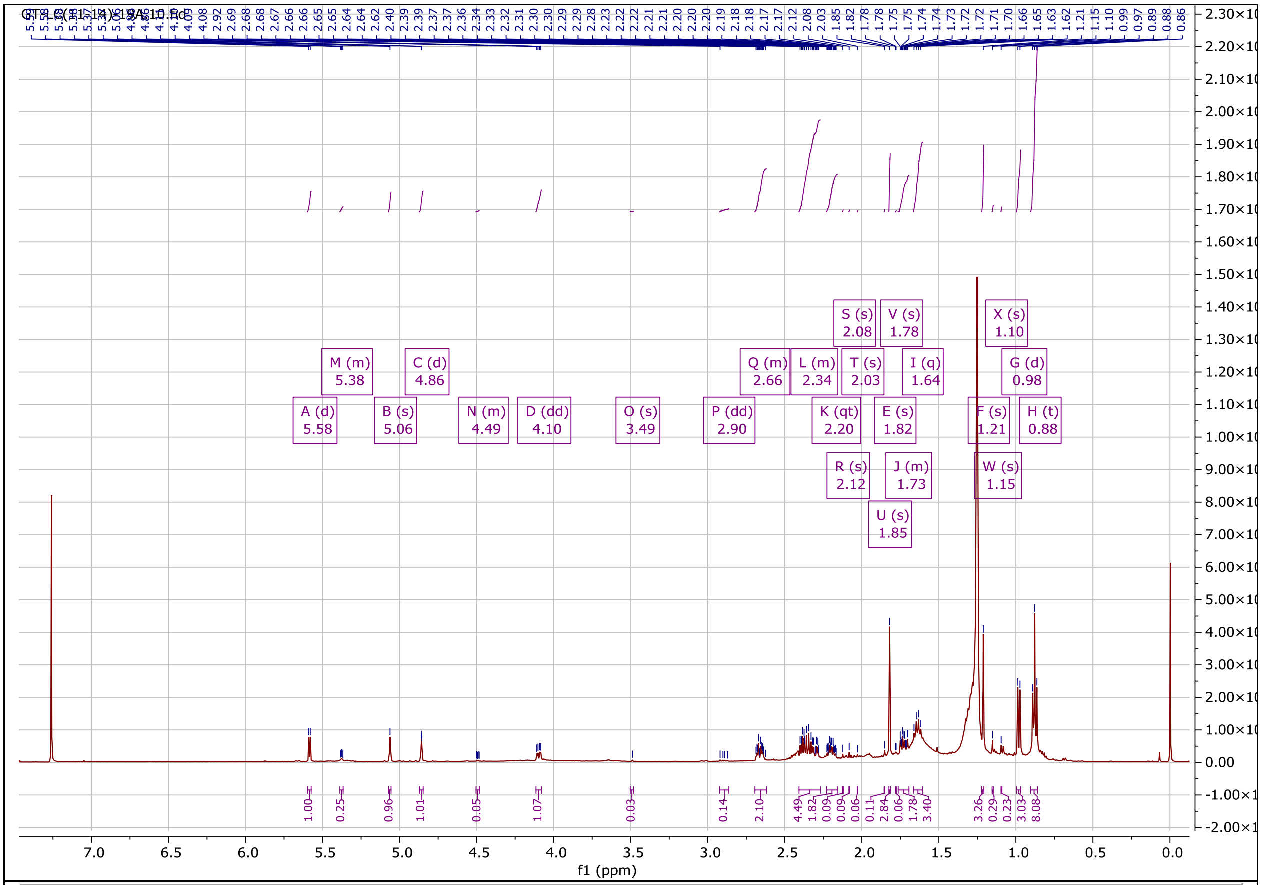

	Shift	Range	Name	H's	Integral	Class	J's
1	5.585	5.60 .. 5.57	A (d)	1	1.000	d	4.66
2	5.376	5.39 .. 5.37	M (m)	0	**0.253**	m	
3	5.062	5.07 .. 5.06	B (s)	1	0.964	s	
4	4.857	4.87 .. 4.85	C (d)	1	1.012	d	1.54
5	4.491	4.50 .. 4.48	N (m)	0	**0.051**	m	
6	4.096	4.11 .. 4.08	D (dd)	1	1.069	dd	3.72, 9.20
7	3.489	3.50 .. 3.48	O (s)	0	**0.029**	s	
8	2.896	2.92 .. 2.86	P (dd)	0	**0.143**	dd	9.26, 14.91
9	2.664	2.69 .. 2.62	Q (m)	2	2.102	m	
10	2.343	2.41 .. 2.27	L (m)	4	4.494	m	
11	2.201	2.23 .. 2.16	K (qt)	2	1.822	qt	3.23, 3.23, 7.36, 7.36, 7.36
12	2.123	2.13 .. 2.12	R (s)	0	**0.093**	s	
13	2.082	2.08 .. 2.08	S (s)	0	**0.089**	s	
14	2.028	2.03 .. 2.03	T (s)	0	**0.057**	s	
15	1.852	1.86 .. 1.85	U (s)	0	**0.114**	s	
16	1.819	1.82 .. 1.81	E (s)	3	2.843	s	
17	1.777	1.78 .. 1.78	V (s)	0	**0.057**	s	

18	1.731	1.76 .. 1.69	J (m)	2	1.778	m	
19	1.639	1.66 .. 1.61	I (q)	3	3.403	q	7.39, 7.39, 7.50
20	1.212	1.22 .. 1.21	F (s)	3	3.260	s	
21	1.151	1.15 .. 1.15	W (s)	0	**0.289**	s	
22	1.095	1.10 .. 1.09	X (s)	0	**0.234**	s	
23	0.980	1.00 .. 0.97	G (d)	3	3.025	d	6.94
24	0.877	0.90 .. 0.86	H (t)	8	8.076	t	6.89, 6.89
				**total**	**36,257**		
				**analyte signals**	**34,848**		
				**%**	**96,114**		

Table S8. Impurity of the Compound **4** (LC(11-14)-19B) by qHNMR

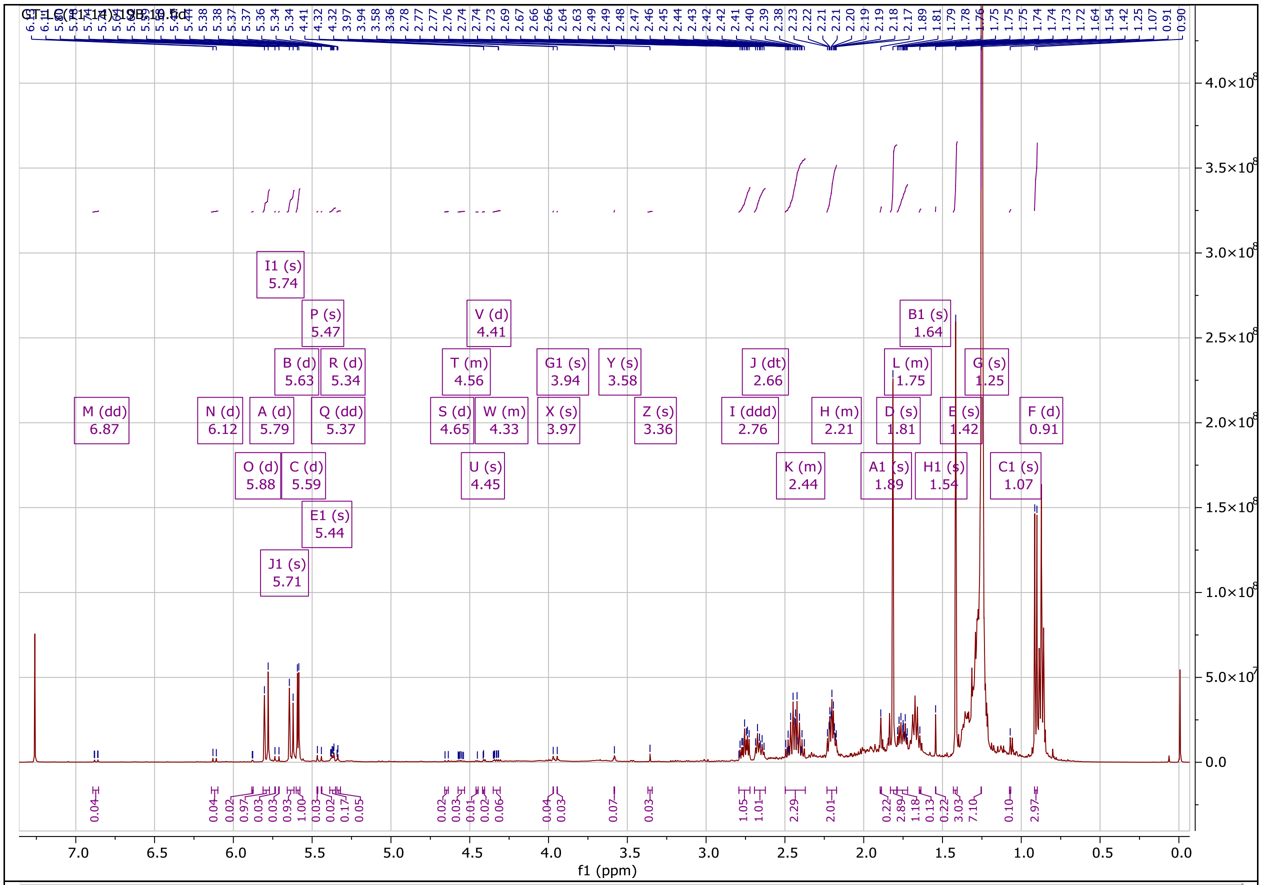

Multiplets (Compound **4**)

	Shift	Range	Name	H's	Integral	Class	J's
1	6.871	6.89 .. 6.86	M (dd)	0	**0.040**	dd	1.16, 10.72
2	6.119	6.14 .. 6.10	N (d)	0	**0.040**	d	10.79
3	5.879	5.88 .. 5.87	O (d)	0	**0.023**	d	1.53
4	5.791	5.81 .. 5.77	A (d)	1	0.966	d	12.02
5	5.736	5.74 .. 5.73	I1 (s)	0	**0.028**	s	
6	5.710	5.71 .. 5.71	J1 (s)	0	**0.027**	s	
7	5.633	5.66 .. 5.61	B (d)	1	0.930	d	11.96
8	5.589	5.60 .. 5.58	C (d)	1	1.000	d	4.51
9	5.467	5.47 .. 5.46	P (s)	0	**0.029**	s	
10	5.441	5.44 .. 5.44	E1 (s)	0	**0.023**	s	
11	5.370	5.39 .. 5.35	Q (dd)	0	**0.171**	dd	2.68, 5.65
12	5.339	5.34 .. 5.32	R (d)	0	**0.049**	d	2.59
13	4.646	4.66 .. 4.64	S (d)	0	**0.023**	d	10.42
14	4.557	4.57 .. 4.53	T (m)	0	**0.028**	m	
15	4.452	4.46 .. 4.45	U (s)	0	**0.007**	s	
16	4.413	4.42 .. 4.41	V (d)	0	**0.015**	d	1.43
17	4.333	4.35 .. 4.31	W (m)	0	**0.059**	m	

18	3.970	3.97 .. 3.97	X (s)	0	**0.039**	s	
19	3.945	3.95 .. 3.94	G1 (s)	0	**0.028**	s	
20	3.582	3.58 .. 3.58	Y (s)	0	**0.070**	s	
21	3.356	3.37 .. 3.34	Z (s)	0	**0.028**	s	
22	2.756	2.79 .. 2.72	I (ddd)	1	1.050	ddd	5.56, 8.81, 14.44
23	2.659	2.69 .. 2.62	J (dt)	1	1.011	dt	6.12, 6.12, 14.44
24	2.436	2.50 .. 2.37	K (m)	2	2.287	m	
25	2.207	2.23 .. 2.17	H (m)	2	2.008	m	
26	1.892	1.90 .. 1.89	A1 (s)	0	**0.216**	s	
27	1.815	1.83 .. 1.79	D (s)	3	2.887	s	
28	1.755	1.79 .. 1.72	L (m)	1	1.184	m	
29	1.644	1.65 .. 1.64	B1 (s)	0	**0.127**	s	
30	1.544	1.54 .. 1.54	H1 (s)	0	**0.219**	s	
31	1.416	1.43 .. 1.41	E (s)	3	3.027	s	
32	1.254	1.26 .. 1.25	G (s)	7	7.100	s	
33	1.070	1.08 .. 1.07	C1 (s)	0	**0.098**	s	
34	0.908	0.92 .. 0.90	F (d)	3	2.967	d	6.87
				**total**	**27,804**		
				**analyte signals**	**26,417**		
				**%**	**95,012**		
